# Metabolic buffering restricts phenotype switching in melanoma

**DOI:** 10.1038/s44318-026-00857-2

**Published:** 2026-07-15

**Authors:** Ana Ramírez-Sánchez, Miguel Jociles-Ortega, José Manuel García-Martínez, Irene Torrens-Martínez, Javier Martínez-Useros, Pakavarin Louphrasitthiphol, Mollie I Sweeney, Ana Morente-Carrasco, Sergio Ruiz-Reyes, Nerea Redondo-Díaz, Teresa Olmos-De Blas, María Jesús Fernández-Aceñero, José N Rodriguez-López, Tomoyoshi Soga, Richard M White, Colin R Goding, Luis Sanchez-Del-Campo, Custodia García-Jiménez, Ana Chocarro-Calvo

**Affiliations:** 1 Area of Physiology, Faculty Health Sciences, University Rey Juan Carlos, 28922 Alcorcón, Madrid Spain; 2 Translational Oncology Division, OncoHealth Institute, Health Research Institute-University Hospital Fundación Jiménez Díaz-Universidad Autónoma de Madrid, 28040 Madrid, Spain; 3 Ludwig Institute for Cancer Research, Nuffield Department of Clinical Medicine, University of Oxford, Old Road Campus Research Building, Old Road Campus, Headington, Oxford, OX3 7DQ UK; 4 Department of Surgical Pathology, Hospital Clínico San Carlos, 28040 Madrid, Spain; 5 Department of Biochemistry and Molecular Biology A, School of Biology, University of Murcia, Instituto Murciano de Investigación Biosanitaria (IMIB), 30120 Murcia, Spain; 6 Human Biology-Microbiome-Quantum Research Center (WPI-Bio2Q), Keio University, Tsuruoka, Yamagata 997-0052 Japan

**Keywords:** Cancer, Metabolism

## Abstract

The impact of the metabolic microenvironment on epigenetically plastic cancer cells underpins phenotypic heterogeneity, a major cause of metastasis and therapy resistance. Nutrient limitation is a key microenvironmental stress, and can cause cells to transition from proliferative to invasive phenotypes, however, whether cancer cells have the capacity to delay phenotype switching remains unknown. Here, using melanoma as a model, we reveal that the ability to buffer glucose availability by accumulating and mobilizing glycogen can determine cancer cell phenotypic transitions. While proliferating cells contain high levels of glycogen, invasive cells are marked by depleted glycogen stores. Accordingly, the inability to store and metabolize glycogen leads to phenotype instability and a switch from proliferation to invasion. The amount of stored glycogen inversely correlates with tissue invasion depth in primary melanomas, and reduced expression of the glycogen phosphorylases PYGB/L and phosphoglucomutase 1 (PGM1) is associated with worse patient survival. Together, we identify metabolic glucose buffering as a determinant of invasive phenotype transitions in skin cancer, suggesting similar paradigms in other cancer types.

## Synopsis

Cancer cells adapt their metabolism to extracellular nutrient fluctuations, yet how intracellular energy reserves influence cell-state transitions remains unclear. This study identifies glycogen as a metabolic buffer in dividing melanoma cells, sustaining carbon supply under starvation, and controlling invasive state transitions.

- Melanoma phenotypes can be distinguished by their ability to store and mobilise glycogen.
- Glycogen fuels glycolysis in melanoma cells with high expression of the transcription factor MITF, suppressing invasiveness under low-glucose stress.
- MITF targets expression of glycogen-converting enzymes PGM1 and PYGB.
- Reduced expression of PGM1 and PYGB correlates with advanced melanoma disease stages and worse patient survival.
- Inhibition of glycogen mobilisation favours invasion and metastasis in mice and fish.

## Abstract (web summary)

Storing sugar in glycogen depots protects proliferative skin cancer cells from transient glucose deprivation.

## Graphical abstract

**Figure:**
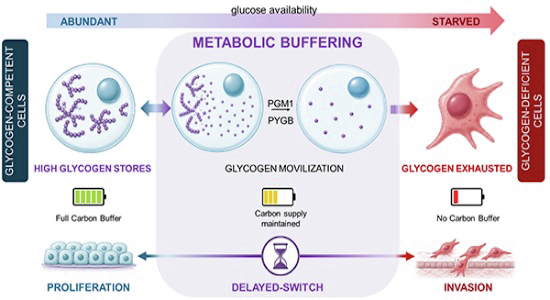

**Figure:**
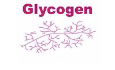

## Introduction

Cancer cell phenotypic heterogeneity underpins metastasis, contributes to therapy resistance, and poses a major therapeutic challenge. In addition to changes in metabolism and gene expression driven by oncogene activation, in response to a changing microenvironment, cancer cells undergo dynamic and reversible phenotypic transitions. These are accompanied by both metabolic rewiring (Falletta et al, 2022) and remodeling of the epigenetic landscape, a hallmark of malignant transformation across all cancer types (Marusyk et al, 2012). Different phenotypic states within a tumor can therefore exhibit fundamentally different metabolic profiles (Jose et al, 2011). Changes in metabolism and any associated phenotypic transition may, in principle, be suppressed by the presence of “metabolic reservoirs” that might buffer against perturbations in nutrient availability, one of the primary causes of phenotype switching in cancer (García-Jiménez and Goding, 2019). The concept of metabolic reservoirs in cancer is relatively new (Zhang et al, 2022) and envisages that energy-producing molecules can be stored by cancer cells for later use. One key metabolic reservoir is glycogen that contributes to glucose homeostasis in cancer cells by supplying glucose for glycolysis and carbons for the pentose phosphate pathway and the tricarboxylic acid cycle (Zois et al, 2014). However, whether the amount of glycogen stored in a cell or glycogen metabolism in general plays a critical role in phenotype-switching and contributes to the generation of intra-tumoral heterogeneity is poorly understood.

Melanoma represents an excellent model for studying phenotype-switching. Multiple phenotypic states are well-defined by the expression of key markers, including the microphthalmia-associated transcription factor (MITF). MITF controls many features of melanoma biology (Goding and Arnheiter, 2019) and promotes either differentiation or proliferation, regulates metabolism and DNA damage repair, and suppresses invasion. Cell intrinsic events, such as activation of BRAF, or microenvironmental factors, such as glutamine (Falletta et al, 2017) or glucose limitation (Ferguson et al, 2017), downregulate MITF mRNA and protein expression and lead to a reversible switch from proliferation to invasion. Significantly, the switch from an MITF^High^ to MITF^Low^ state is accompanied by altered metabolism; proliferative phenotype melanoma cells depend primarily on glycolysis (Parmenter et al, 2014), whereas invasive melanoma cells, that are also slow-cycling and therapy resistant, obtain their energy primarily through oxidative phosphorylation (Zhang et al, 2016).

Here, using melanoma as a model, we explore whether metabolic buffering, via the accumulation of glucose stores in the form of glycogen, impacts the ability of melanoma to undergo phenotypic transitions. The results reveal that accumulation of glycogen is a hallmark of proliferative, but not invasive phenotype cells, and that low glycogen stores lead to phenotypic instability and a rapid transition to invasion.

## Results

### Proliferative, MITF^High^, melanoma cells accumulate glycogen and are highly glycolytic

**Figure 1:**
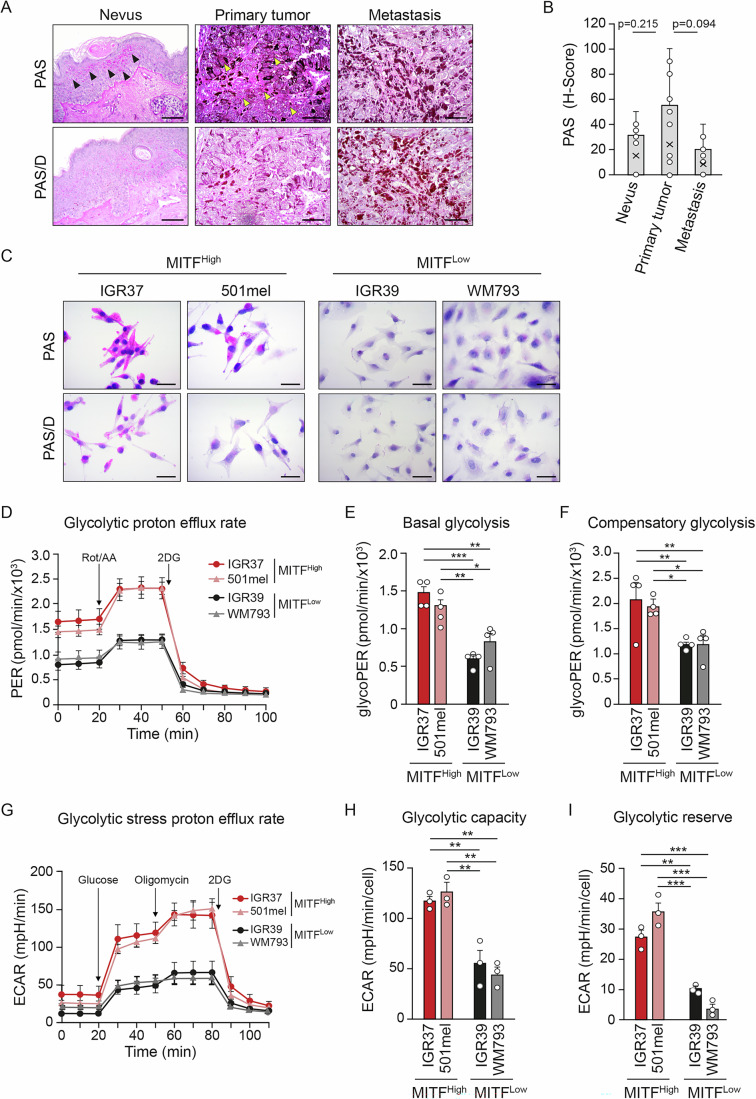
Glycogen stores distinguish melanoma phenotypes. (**A**) Immunohistochemical analysis of glycogen content in human melanoma biopsies of nevus (left), melanoma primary tumor (middle) and metastasis (right) stained with Periodic Acid-Shiff (PAS) (upper) or digested with diastase (PAS/D) (lower) to assess the specific glycogen staining in consecutive sections. Arrows show glycogen stores localization in purple; brown color represents tissue pigmentation (melanin). Scale bars: 50 μm. (**B**) Quantification of PAS staining from human samples in (**A**) *n* = 14 for nevus, *n* = 27 for primary melanoma, and *n* = 28 for metastasis. Staining intensity was quantified by Histoscore (H-Score). Box plots show median with lower (25%) and upper (75%) quartiles; whiskers represent minimum and maximum values. Statistical analysis by the Mann–Whitney *U*-test. *P* value *p < 0.05. (Nevus vs. primary tumor: *P* = 0.215; Primary tumor vs. metastasis: *P* = 0.094). (**C**) Glycogen stores in proliferative IGR37 and 501mel (MITF^High^) and invasive IGR39 and WM793 (MITF^Low^) melanoma cells were revealed by PAS staining as above. Scale bars: 25 μm. (**D**) Glycolytic proton efflux rate (PER) measured by Seahorse assay in MITF^High^ (IGR37, *n* = 4; 501mel, *n* = 4) and MITF^Low^ (IGR39, *n* = 4; WM793, *n* = 5) melanoma cells. Data represent mean ± SEM. (**E**, **F**) Quantification of glycolytic parameters derived from (**D**). Bar graphs represent Basal (**E**) and Compensatory (**F**) glycolysis rates. *n* = 4. (**G**) Extracellular acidification rate (ECAR) of IGR37 and 501mel (MITF^High^) and IGR39 and WM793 (MITF^Low^) melanoma cells. *n* = 3 independent experiments. Data represent mean ± SEM. (**H**, **I**) Quantification of glycolytic capacity (**H**) and glycolytic reserve (**I**) derived from (**G**). *n* = 3 independent experiments. Panels (**E**, **F**, **H**, **I**) show mean ± SEM. Statistical analysis by one-way ANOVA. *P < 0.05; **P < 0.01; ***P < 0.001. (**E**) IGR37 vs IGR39 P = 0.0001; IGR37 vs WM793 *P* = 0.0028; 501mel vs IGR39 *P* = 0.0015; 501mel vs WM793 *P* = 0.0424. (**F**) IGR37 vs IGR39 *P* = 0.0068; IGR37 vs WM793 *P* = 0.0084; 501mel vs IGR39 *P* = 0.0262; 501mel vs WM793 *P* = 0.0211. (**H**) IGR37 vs. IGR39 *P* = 0.0076; IGR37 vs WM793 *P* = 0.0024; 501mel vs IGR39 *P* = 0.0032; 501mel vs WM793 *P* = 0.0011. (**I**) IGR37 vs IGR39 *P* = 0.0022; IGR37 vs WM793 *P* = 0.0002; 501mel vs IGR39 *P* = 0.0001; 501mel vs. WM793 *P* = 0.0001. Source data are available online for this figure.

Since proliferating cancer cells have increased demands for glucose and intracellular glycogen stores have been observed in various tumors (Khan et al, 2020), we set out to determine the role and importance of glycogen storage in melanoma by first characterizing the glycogen content in human tumors. Periodic acid–Schiff stain (PAS) with or without the glycogen-degrading enzyme amylase (PAS-D), as a control for the specificity of the staining, was performed in patient melanoma samples as well as in nevi. About 67 samples were included in total, of which 14 were nevi (20.9%), 26 primary melanomas (38.8%) and 27 metastasis (40.3%) (see Table 1 for patient and tumor characteristics). Nevi were characterized by small glycogen accumulations in the upper layers of the epidermis, evidenced by intense magenta staining that disappeared when pre-digested with diastase (PAS/D) (Fig. 1A, left panels). In contrast, primary melanomas were either PAS-negative or positive with glycogen-specific staining localized throughout the tissue in PAS-positive melanomas (Figs. 1A, middle panels and EV1A). Consistently, only a few metastases showed glycogen accumulation and in those metastases with glycogen there was only a minor staining (Figs. 1A right panels and EV1A), that was quantified to show much less glycogen than primary melanoma samples (Fig. 1B) with a trend towards significance (*p* = 0.094).

**Figure EV1:**
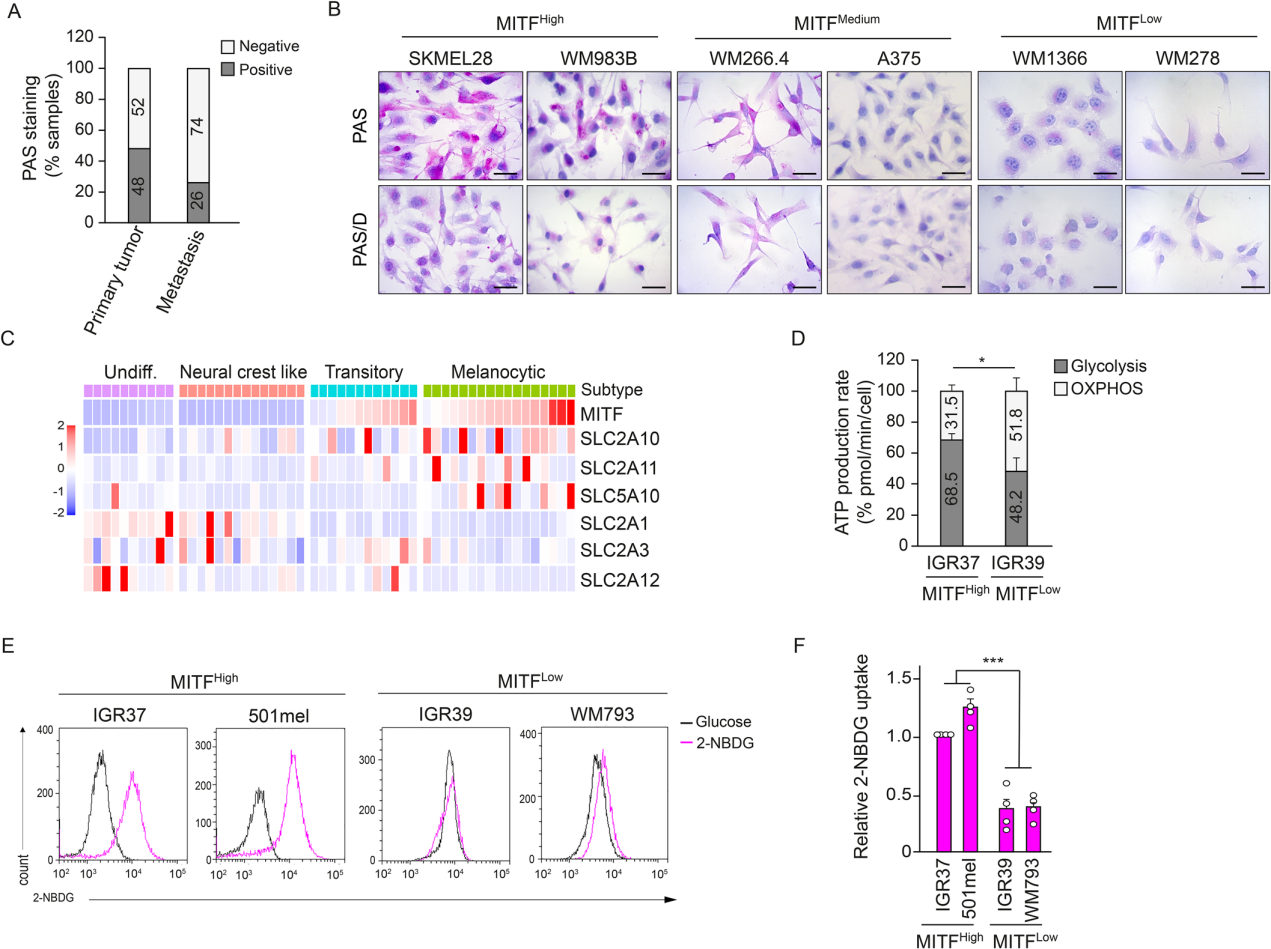
Glucose uptake and storage in melanoma. (**A**) Graphic representation of the percentage (%) of samples that store (PAS-Positive) and that do not store glycogen (PAS-Negative) in our collection of melanoma and metastasis biopsies. (**B**) Glycogen stores in SKMEL28 and WM983B (MITF^High^), WM266.4 and A375 (MITF^Medium^), and WM1366 and WM278 (MITF^Low^) melanoma cells were revealed by PAS/PAS-D staining. Representative images, scale bars: 25 μm. (**C**) Heatmap showing relative expression of MITF and glucose transporters: SLC2A10, SLC2A11, SLC5A10, SLC2A1, SLC2A3, and SLC2A12 in the CCLE collection of cell lines classified by phenotype (Tsoi et al, 2018). (**D**) Glycolytic vs mitochondrial oxidative phosphorylation (OXPHOS) production of ATP expressed as percentages, and comparing MITF^High^ (IGR37) and MITF^Low^ (IGR39) melanoma cells. *n* = 4. Data represent mean ± SEM. Statistical analysis by paired *t*-test. *P < 0.05. IGR37 vs. IGR39, *P* = 0.022. (**E**,** F**) Glucose uptake evaluated by flow cytometry in live MITF^High^ (IGR37 and 501mel) and MITF^Low^ (IGR39 and WM793) melanoma cells incubated with 10 µM of the fluorescent glucose analog 2-NBDG for 30 min in the absence of glucose. Representative flow cytometry histograms (**E**) and quantification of mean fluorescence intensity (**F**). *n *= 4. Data represent mean ± SEM. Statistical analysis by one-way ANOVA. *P < 0.05; **P < 0.01; ***P < 0.001. Exact *P* values: IGR37 vs. IGR39, *P* = 0.0001; IGR37 vs. WM793, *P* = 0.0002; 501mel vs. IGR39, *P* = 0.0001; 501mel vs. WM793, *P* = 0.0001.

**Table 1:** Clinicopathological characteristics of patients.

Patients Characteristic	*N* (%)		
	Nevus	Primary melanoma	Metastasis
Gender			
Male	4 (28,6%)	11 (40,7%)	15 (53,6%)
Female	10 (71,4%)	15 (55,6%)	12 (42,8%)
N/A	0	1 (3,7%)	1 (3,6%)
Median age (years)			
Melanoma classification			
MES		4 (28.6%)	
MN		6 (42.9%)	
LMM		3 (21.4%)	
MLA		1 (7.1%)	
Breslow Index			
In situ		4 (13.3%)	
Intermediate		11 (36.7%)	
High		15 (50%)	
Clark level			
I		2 (7,7%)	
II		2 (7,7%)	
III		2 (7,7%)	
IV		15 (57,7%)	
V		4 (15,4%)	
N/A		0	
Development of metastasis			
No		13 (50%)	
Yes		13 (50%)	
Metastasis localization			
Skin (local)		12 (70.6%)	7 (31.8%)
Distant		5 (29.4%)	15 (68.2%)

The variations in glycogen storage in different melanoma stages led us next to investigate whether different melanoma phenotypes exhibit distinct capacities to synthesize or store glycogen. While melanoma cells exhibit plasticity in vivo, once cultured in vitro, each line maintains a fixed cell state (Tsoi et al, 2018) that reflects one of the phenotypes found within tumors. With this in mind, we evaluated the glycogen content in vitro using melanoma cell lines. Four cell lines with proliferative phenotypes (IGR37, 501mel, SK-MEL-28, and WM983B) characterized by high levels of MITF (MITF^High^) were compared with four melanoma cell lines with an MITF^Low^ invasive phenotype (IGR39, WM793, WM1366 and WM278). Two cell lines with intermediate phenotype and moderate levels of MITF (MITF^Medium^) (WM266.4 and A375) were also included. The results revealed abundant glycogen stored in MITF^High^ cells and its absence in MITF^Low^ cells (Figs. 1C and EV1B); intermediate phenotypes showed variability, and glycogen was found in only one of them (Fig. EV1B). Seahorse analysis of the glycolytic proton efflux rate (PER) revealed that MITF^High^ cells displayed a distinct and more glycolytic profile compared to invasive MITF^Low^ cells (Fig. 1D). Moreover, when glucose was abundant, MITF^High^ cells displayed significantly higher basal (Fig. 1E) and compensatory (Fig. 1F) glycolysis. One possible explanation for the observed glycolytic differences between MITF^High^ and MITF^Low^ cells is altered glucose uptake. We therefore examined the mRNA expression of a series of passive glucose transporters (of the GLUT family) encoded by the *SLC2A* gene family in a panel of 53 well-characterized melanoma cell lines that fall into four distinct phenotypes based on their gene expression profiles (Tsoi et al, 2018). We also included the *SLC5A10* gene, a secondary active transporter that couples glucose uptake to Na^+^ influx and can concentrate glucose intracellularly against its gradient (Gyimesi et al, 2020) and compared its expression with that of MITF, a marker for the more proliferative/differentiated cells. The results (Fig. EV1C) show that melanocytic melanoma cells express higher levels of SLC2A10 and SLC2A11, members of the GLUT family, and of the active transporter encoded by SLC5A10, that can complement GLUT‑mediated facilitated uptake. The undifferentiated or neural crest-like MITF^Low^ invasive cells express higher levels of SLC2A1 (GLUT1) and SLC2A3 (GLUT3) that are associated with high affinity/high demand for glucose and typically correlate with a more glycolytic, aggressive phenotype (Liu et al, 2024). However, increased GLUT1/3 expression does not necessarily translate into elevated functional glucose uptake and glycolytic flux, particularly in cells that exhibit OXPHOS‑dependent metabolic state. Consistently, ATP production rate assays confirmed that the MITF^Low^ IGR39 cells are more dependent on mitochondrial OXPHOS for ATP production (Fig. EV1D).

The observation that each cell line expressed a distinct GLUT/SGLT mRNA profile, yet only MITF^High^ cells displayed a glycolytic phenotype, prompted us to analyze differences in glucose uptake. Flow cytometry following NBD-2-deoxy-glucose uptake shows a shift to the right in melanoma MITF^High^ IGR37 and 501mel cells that was absent in the MITF^Low^ IGR39 and WM793 cells and corresponds to a 60% lower glucose uptake in MITF^Low^ melanoma cells. (Fig. EV1E, quantified in EV1F). The dependency of MITF^High^ cells on glycolysis as an energy source was determined by measuring the extracellular acidification rate (ECAR) with a glycolytic stress test, where mitochondria are blocked. In line with our previous results, MITF^High^ cells were highly dependent on glycolysis (Fig. 1G) compared to MITF^Low^ (IGR39 and WM793) that lack glycogen. MITF^High^ cells catabolize glucose through the glycolytic pathway with the extrusion of protons into the surrounding medium that causes a rapid increase in ECAR. Inhibition of mitochondrial ATP production using oligomycin shifts energy production towards glycolysis with a subsequent increase in ECAR and reveals the cellular maximum glycolytic capacity. As expected, MITF^High^ IGR37 and 501mel cells show higher glycolytic capacity than MITF^Low^ IGR39 and WM793 cells (Fig. 1H). The glycolytic reserve, defined as the difference between maximal glycolytic capacity and basal glycolytic rate, was dramatically reduced from MITF^High^ (IGR37 and 501mel) to MITF^Low^ (IGR39 and WM793) cells (Fig. 1I), consistent with the presence of glycogen stores.

Together, the data indicates that well-defined melanoma phenotypes (proliferative MITF^High^ and invasive MITF^Low^) differ in their ability to store and mobilize glycogen and suggest that this metabolic adaptation could play an important role in melanoma progression.

### Proliferative, MITF^High^ melanoma cells mobilize glycogen under stress conditions

**Figure 2:**
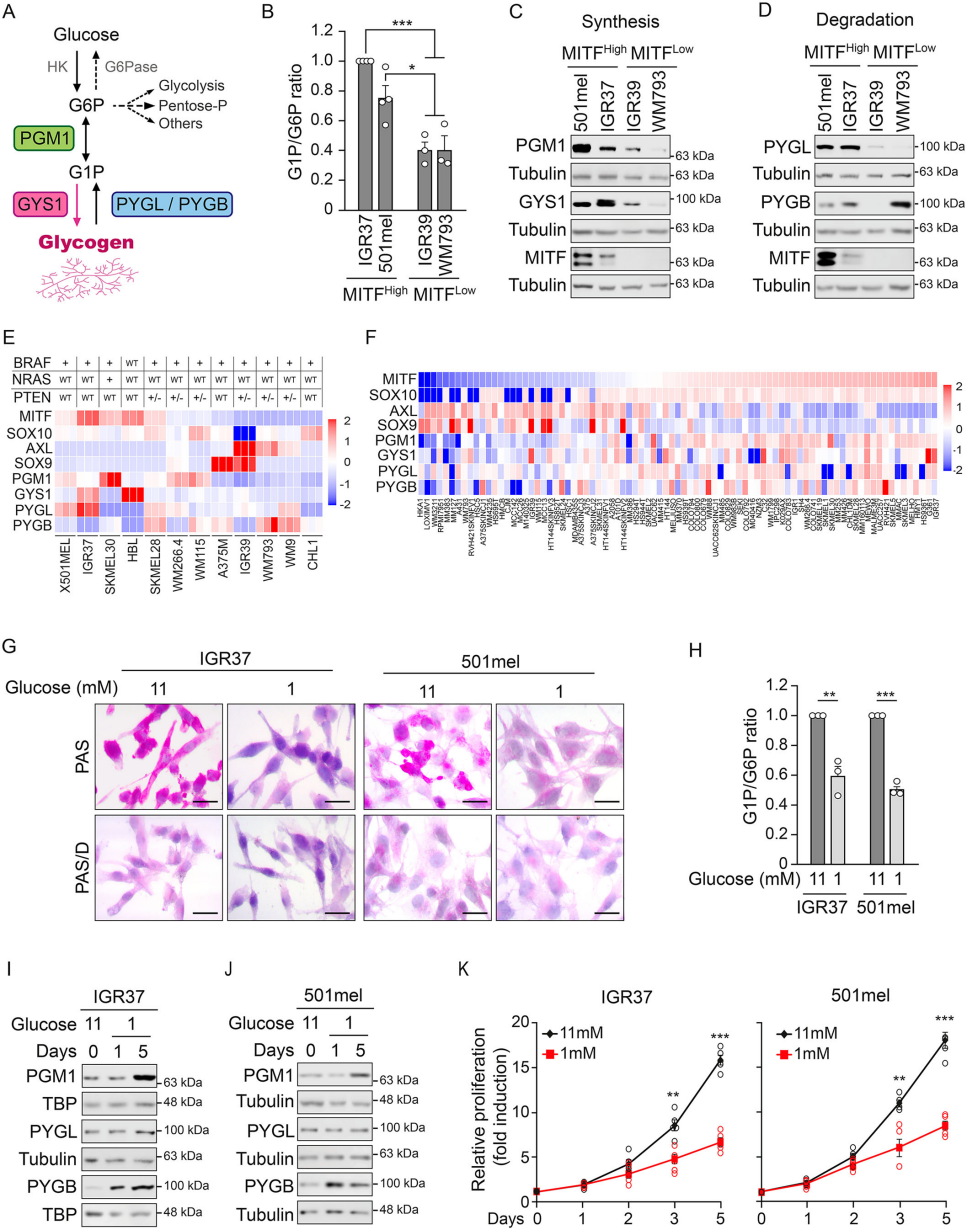
Melanoma cell phenotypes express different levels of glycogen metabolism enzymes. (**A**) Schematic showing the glycogen metabolism pathway with the main synthesis and degradation enzymes. (**B**) Changes in the relative amounts of glucose 1 phosphate (G1P) and glucose 6-phosphate (G6P) in MITF^High^ (IGR37, 501mel) and MITF^Low^ (IGR39 and WM793) melanoma cells, expressed as G1P/G6P ratio. n = 4 (IGR37, 501mel); *n* = 3 (IGR39, WM793). Data represent mean ± SEM. Statistical analysis by one-way ANOVA. *P < 0.05; **P < 0.01; ***P < 0.001. IGR37 vs. IGR39, *P* = 0.0008; IGR37 vs. WM793, *P* = 0.0008; 501mel vs. IGR39, *P* = 0.0331; 501mel vs. WM793, *P* = 0.0331. (**C**, **D**) Western blot showing expression in the same panel of melanoma cells of enzymes that participate in glycogen synthesis, PGM1 and GYS1 (**C**) or glycogen degradation, PYGL and PYGB (**D**). Tubulin as loading control. (**E**) Heatmap showing expression of MITF, SOX10, AXL, SOX9, PGM1, GYS1, PYGL, and PYGB mRNA in an in-house panel of 12 melanoma cell lines measured by triplicate RNA-seq or (**F**) in the panel of CCLE 53 melanoma cell lines ranked by MITF expression. The mutation status of BRAF, NRAS and PTEN is indicated in E, where “+” denotes an activating mutation. (**G**) Representative images of glycogen accumulation in MITF^High^ (IGR37, 501mel) melanoma cells in high glucose (11 mM) or low glucose (1 mM), stained with PAS (upper panels) and treated with diastase (PAS-D) (lower panels) to assess the specific glycogen staining. Scale bars: 25 μm. (**H**) G1P/G6P ratio measurement in IGR37 and 501mel melanoma cells treated as in (**G**). *n* = 3. Data represent mean ± SEM. Statistical analysis by paired *t*-test. *P < 0.05; **P < 0.01; ***P < 0.001. IGR37 11 mM vs. 1 mM, *P* = 0.0023; 501mel 11 mM vs. 1 mM, *P* = 0.0001. (**I**, **J**) Western blot analysis of indicated melanoma cells cultured with 11 mM glucose or washed and starved in medium containing only 1 mM glucose for 1 or 5 days. TBP or tubulin were used as a loading control. At least three independent experiments were performed for each marker. (**K**) Effect of glucose depletion (1 mM) on the proliferation of indicated melanoma cells. Black lines represent cells proliferating in high glucose (11 mM), red lines cells cultured in 1 mM glucose. *n* = 5. Mean ± SEM is displayed. Statistical analysis by one-way ANOVA. *P < 0.05; **P < 0.01; ***P < 0.001 IGR37 11 mM vs 1 mM at 3 days, *P* = 0.0077; and at 5 days, *P* < 0.0001; 501mel 11 mM vs 1 mM at 3 days, *P* = 0.0012; and at 5 days, *P* = 0.0001. Source data are available online for this figure.

**Figure EV2:**
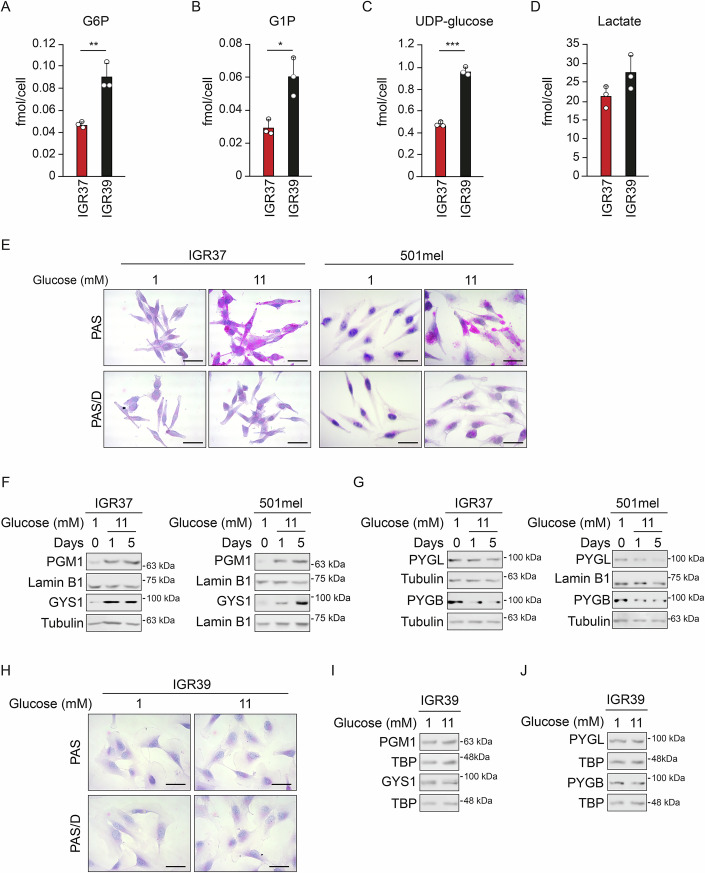
Glycogen intermediaries and glycogen dynamics in melanoma cells. (**A**–**D**) Metabolomic analysis by capillary electrophoresis–mass spectrometry of steady-state levels of G6P (**A**), G1P (**B**), UDP-glucose (**C**), and lactate (**D**) in IGR37 and IGR39 melanoma cells. *n* = 3 biological replicates. Data represent mean ± standard deviation. Statistical analysis by *t*-test. *P < 0.05; **P < 0.01; ***P < 0.001. Exact *P* values (IGR37 vs. IGR39): G6P, *P* = 0.0058; G1P, *P* = 0.0127; UDP-glucose, *P* = 0.0001; Lactate, *P* = 0.1568. (**E**) Glycogen accumulation by IGR37 and 501mel melanoma cells growing in 1 mM glucose for 24 h and incubated with 11 mM glucose for another 24 h, revealed with PAS (purple)/PAS-D staining. Representative images; scale bars: 25 μm. (**F**, **G**) Western blot analysis of whole cell extracts of indicated cells to compare dynamic changes in the levels of the indicated glycogen metabolism enzymes following glucose addition to the culture (from 1 to 11 mM) for 1 or 5 days. Tubulin or Lamin B as loading control. (**H**–**J**) Glycogen dynamics in MITF^Low^ IGR39 melanoma cells growing as in (**E**) for comparison. (**H**) Representative images, scale bars: 25 μm. (**I**, **J**) Western blot analysis of IGR39 treated as in (**D**), whole cell extracts; TBP as loading control. Three independent Western blot experiments were performed for each marker.

Glycogen formation begins with the critical conversion of glucose 6-phosphate (G6P) to glucose 1 phosphate (G1P), a reversible reaction catalyzed by the evolutionarily conserved enzyme Phosphoglucomutase 1 (PGM1) (Fig. 2A); subsequently, G1P is converted to UDP-glucose and used to synthesize glycogen by the enzyme Glycogen Synthase (GYS1), which elongates the chain through alpha 1,4 bonding. Glycogen degradation begins with the activity of the enzyme glycogen phosphorylase (PYG), which removes glucosyl units. Two isoforms are expressed in melanoma cells: PYGL, characteristic of Liver and PYGB, characteristic of Brain and more ubiquitous. The G1P/G6P ratio, regulated by PGM1 activity, reflects the metabolism of glucose (predominance of glycolysis or glycogen storage) in a cell. The G1P/G6P ratio in MITF^High^ and MITF^Low^ melanoma cells was compared under conditions of glucose abundance. Coherent with previous results, MITF^High^ cells showed a higher G1P/G6P ratio, compatible with glycogen storage and increased glycolysis when compared to MITF^Low^ cells (Fig. 2B). To further characterize the metabolic state of MITF^High^ and MITF^Low^ cells under non-limiting glucose conditions, levels of glycolytic and glycogen-related intermediates were measured by metabolomics in MITF^High^ (IGR37) and MITF^Low^ (IGR39) cells (Fig. EV2A–D). IGR37 cells, which harbor the highest intracellular glycogen stores, displayed the lowest levels of G6P, G1P and UDP-glucose (Fig. EV2A–C), consistent with efficient channeling of glucose 6-phosphate into glycogen synthesis and the consequent rapid consumption of upstream intermediates. This pattern is coherent with the elevated G1P/G6P ratio previously observed in MITF^High^ cells (Fig. 2B) and supports the notion that glycolytic flux in these cells is preferentially directed toward glycogen storage rather than toward pyruvate production. In contrast, IGR39 cells, which do not accumulate glycogen and rely predominantly on OXPHOS, exhibited more balanced G6P/G1P ratios; less G6P is committed to pyruvate/mitochondrial ATP production (Fig. EV2A), leaving more G6P available for conversion to G1P and UDP‑glucose despite low glycogen synthesis (Fig. EV2B,C). The hexosamine pathway may also draw on G6P and UDP‑glucose and contribute to metabolite depletion. Although intracellular lactate concentrations were comparable between IGR37 and IGR39 cells (Fig. EV2D), ECAR measurements revealed a marked increase in extracellular acidification in the glycolytic MITF^High^ cells (Fig. 1G), indicative of elevated glycolytic flux. This apparent discrepancy reflects the fundamentally distinct nature of the two measurements: ECAR captures real-time proton extrusion arising from glycolysis, lactate export, CO₂ hydration and proton-handling mechanisms, whereas metabolomic lactate quantification reports steady-state intracellular levels. Accordingly, MITF^High^ cells can sustain higher glycolytic activity and proton release without accumulating measurable differences in intracellular lactate, suggesting efficient lactate export and extracellular buffering.

Furthermore, the levels of the key enzymes for glycogen synthesis (PGM1 and GYS1) were higher in MITF^High^ IGR37 and 501mel cells than in the invasive MITF^Low^ melanoma lines (Fig. 2C). Likewise, the levels of the glycogen degradation enzyme PYGL were increased in MITF^High^ melanoma cells (Fig. 2D). Interestingly, the invasive WM793 cells lost PYGL but increased the alternative isoform, characteristic of brain and tumor tissues, PYGB. Thus, the levels of enzymes for glycogen metabolism correlate with the capacity to store glycogen in melanoma cells. Further, RNAseq was used to compare the mRNA expression of these enzymes in our in-house panel of 12 melanoma cell lines (Fig. 2E), ranked by the expression of MITF as a marker for proliferative/differentiated cells, SOX10, a marker of the differentiated and neural crest-like states, and the AXL receptor tyrosine kinase (RTK) and SOX9 as markers of invasive phenotypes (Müller et al, 2014). To capture the genetic heterogeneity of melanoma, cell lines were additionally annotated for the most prevalent driver mutations in the disease, including BRAF, NRAS and PTEN alterations (Fig. 2E), collectively covering more than 80% of common melanoma genotypes. Notably, IGR37 and IGR39 cells, which are both BRAF^V600E^-mutated and derived from the same patient, differ markedly in their MITF levels and glycogen content, indicating that the relationship between MITF status and glycogen metabolism is independent of this common driver mutation. Remarkably, *PGM1* and *GYS1* expressions correlated with *MITF* expression in melanoma cells (Fig. 2E). Concerning the glycogen degradation enzymes, PYGL mRNA was also expressed predominantly in MITF^High^ cells, and was not found in most MITF^Low^ cell lines (Fig. 2E). Interestingly, PYGB expression appeared high in many MITF^Low^ cell lines, and low in MITF^High^ cells. An alternative panel of well-characterized melanoma cell lines from the Cancer Cell Line Encyclopedia (CCLE) ranked by *MITF* expression was then used to extend our findings (Fig. 2F). The results largely recapitulated those of our in-house cell lines, with the MITF^High^ cells tending to express higher levels of glycogen metabolism genes.

Glycogen mobilization should provide an energetic advantage to melanoma cells under metabolic stress, facilitating tumor progression. Consequently, glycogen stores were depleted in MITF^High^ cells (IGR37 and 501mel) by culture under low glucose (Fig. 2G). This led to a concomitant reduction in the G1P/G6P ratio (Fig. 2H). Under these conditions, PGM1 would be needed to drive G1P from glycogen degradation towards G6P that would enter glycolysis. Consistent with this, 5 days after glucose depletion, PGM1 levels were notably increased (Fig. 2I,J). The key glycogen-degrading enzymes (PYGs) appeared less sensitive to glucose depletion with no changes in PYGL protein levels and increased PYGB levels in cells grown in 1 mM glucose (Fig. 2I,J). Conversely, MITF^High^ cells cultured with high glucose dramatically augmented their glycogen levels (Fig. EV2E) as well as the levels of the enzymes to synthesize glycogen, PGM1 and GYS1 (Fig. EV2F). As with glucose depletion, PYG enzymes were less sensitive to glucose addition; PYGL protein levels remained unchanged, and PYGB levels were reduced (Fig. EV2G). By contrast, IGR39 MITF^Low^ cells were unable to accumulate glycogen despite glucose abundance (Fig. EV2H), and the low protein levels of glycogen metabolism enzymes remained unchanged in response to high glucose exposure (Fig. EV2I,J). In line with these results, glucose deprivation reduced the proliferation rate of MITF^High^ melanoma cells very slowly, with moderate changes only appearing by 48 h (Fig. 2K).

The results indicate that MITF^High^ melanoma cells can accumulate glucose in the form of glycogen under nutrient-rich conditions and expend it to obtain energy under glucose-limited conditions. By contrast, IGR39 cells do not accumulate glycogen and glucose levels do not regulate their levels of enzymes for the synthesis or degradation of glycogen.

### The proliferative capacity of MITF^High^ melanoma cells relies on glycogen mobilization

**Figure 3:**
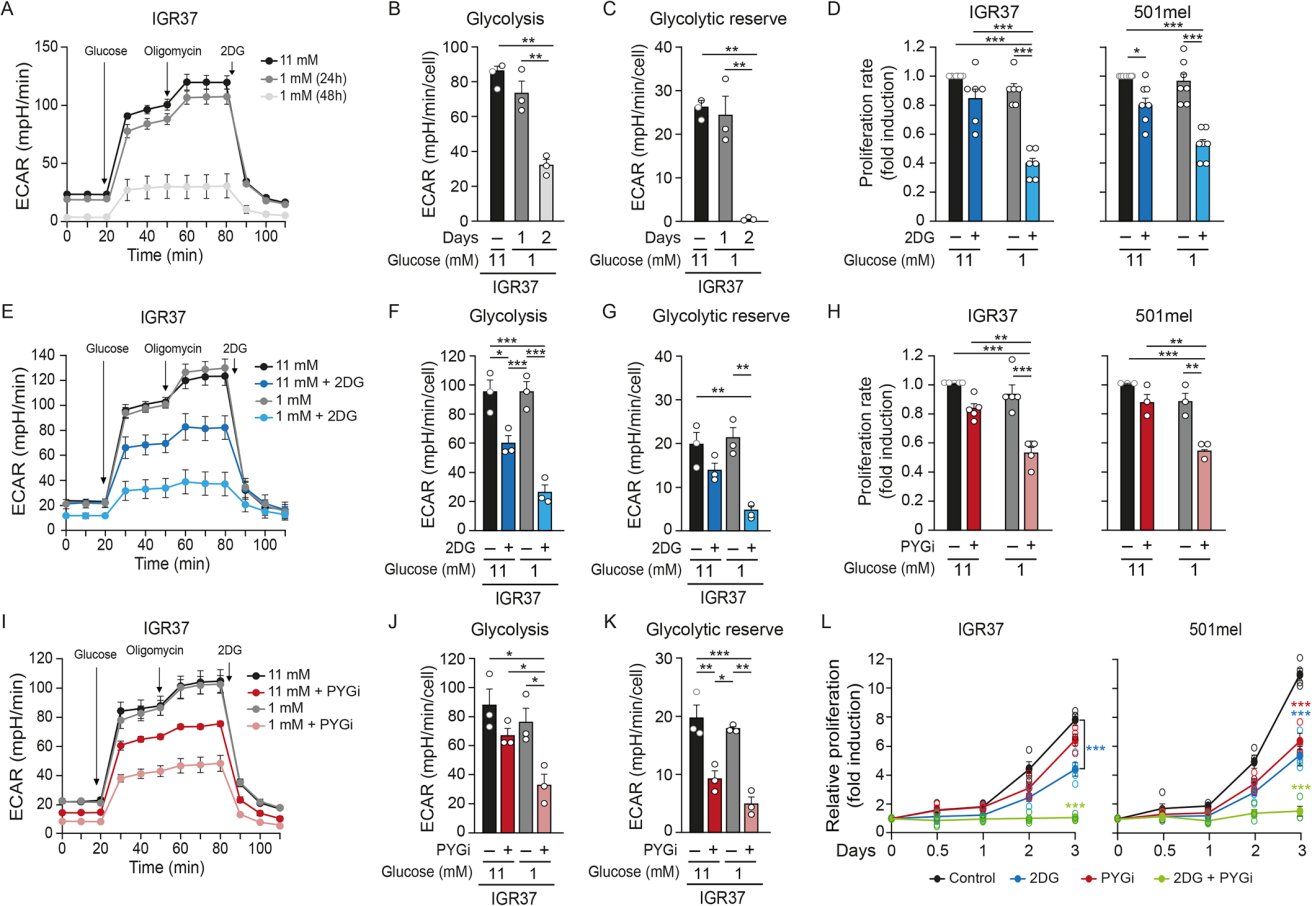
Glycogen fuels glycolysis in MITF^High^ melanoma cells to proliferate under glucose stress conditions. (**A**, **E**, **I**) Extracellular acidification rate (ECAR) of IGR37 melanoma cells cultured under glucose depletion (1 mM) for 1 or 2 days (**A**), pretreated with the glycolysis inhibitor 2-deoxy-D-glucose (2DG) at 1.5 mM (**E**), or with the glycogen phosphorylase inhibitor (PYGi) CP-91149 at 50 µM for 24 h (**I**). *n* = 3 independent experiments. Data represent mean ± SEM. (**B**, **C**, **F**, **G**, **J**, **K**) Quantification of glycolytic parameters derived from (**A**, **E**, **I**), respectively. Bar graphs represent basal glycolysis (**B**, **F**, **J**) and glycolytic reserve (**C**, **G**, **K**). *n* = 3. Data represent mean ± SEM. Statistical analysis by one-way ANOVA. *P < 0.05; **P < 0.01; ***P < 0.001. (**D**, **H**) Melanoma cell proliferation in response to glucose depletion or 1.5 mM 2DG (**D**), or 50 µM PYGi (**H**), for 24 h. *n* = 6 (IGR37) and *n* = 7 (501mel) in (**D**); *n* = 5 (IGR37) and *n* = 3 (501mel) in (**H**). Data represent mean ± SEM. Statistical analysis by one-way ANOVA. *P < 0.05; **P < 0.01; ***P < 0.001. (**L**) Proliferation curves of IGR37 and 501mel melanoma cells treated with 2DG (1.5 mM, blue), PYGi (50 µM, red), or both (green), compared to control cells cultured in 11 mM glucose (black). *n* = 4. Data represent mean ± SEM. Statistical analysis by one-way ANOVA. *P < 0.05; **P < 0.01; ***P < 0.001. Exact *P* values: (**B**) 11 mM vs. 1 mM day 1, *P* = 0.0011; 11 mM vs. 1 mM day 2, *P* = 0.0041. (**C**) 11 mM vs. 1 mM day 1, *P* = 0.0016; 11 mM vs. 1 mM day 2, *P* = 0.0021. (**F**) 11 mM control vs. 11 mM 2DG, *P* = 0.0345; 11 mM control vs. 1 mM 2DG, *P* = 0.0005; 11 mM 2DG vs. 1 mM, *P* = 0.0001; 1 mM control vs. 1 mM 2DG, *P* = 0.0005. (**G**) 11 mM vs. 1 mM 2DG, *P* = 0.0041; 1 mM control vs. 1 mM 2DG, *P* = 0.0021. (**J**) 11 mM vs. 1 mM PYGi, *P* = 0.0113; 1 mM control vs. 1 mM PYGi, *P* = 0.0425; 11 mM PYGi vs. 1 mM PYGi, *P* = 0.0203. (**K**) 11 mM control vs. 11 mM PYGi, *P* = 0.0066; 11 mM control vs. 1 mM PYGi, *P* = 0.0006; 11 mM PYGi vs. 1 mM, *P* = 0.0188; 1 mM control vs. 1 mM PYGi, *P* = 0.0012. (**D**) For IGR37: 11 mM vs. 1 mM 2DG, *P* = 0.0001; 11 mM 2DG vs. 1 mM 2DG, *P* = 0.0001; 1 mM control vs. 1 mM 2DG, *P* = 0.0001. For 501mel: 11 mM control vs. 11 mM 2DG, *P* = 0.0443; 11 mM vs. 1 mM 2DG, *P* = 0.0001; 1 mM control vs. 1 mM 2DG, *P* = 0.0001. (**H**) For IGR37: 11 mM vs. 1 mM PYGi, *P* = 0.0001; 11 mM PYGi vs. 1 mM PYGi, *P* = 0.0012; 1 mM control vs. 1 mM PYGi, *P* = 0.0001. For 501mel: 11 mM vs. 1 mM PYGi, *P* = 0.0007; 11 mM PYGi vs. 1 mM PYGi, *P* = 0.0041; 1 mM control vs. 1 mM PYGi, *P* = 0.0041. (**L**) For IGR37: Control vs. 2DG, *P* = 0.0002; Control vs. 2DG  + PYGi, *P* = 0.0001; 2DG vs. 2DG + PYGi, *P* = 0.0002; PYGi vs. 2DG  + PYGi, *P* = 0.0001. For 501mel: Control vs. 2DG, *P* = 0.0001; Control vs. PYGi, *P* = 0.0001; Control vs. 2DG + PYGi, *P* = 0.0001; 2DG vs. 2DG + PYGi, *P* = 0.0007; PYGi vs. 2DG + PYGi, *P* = 0.0001. Source data are available online for this figure.

**Figure EV3:**
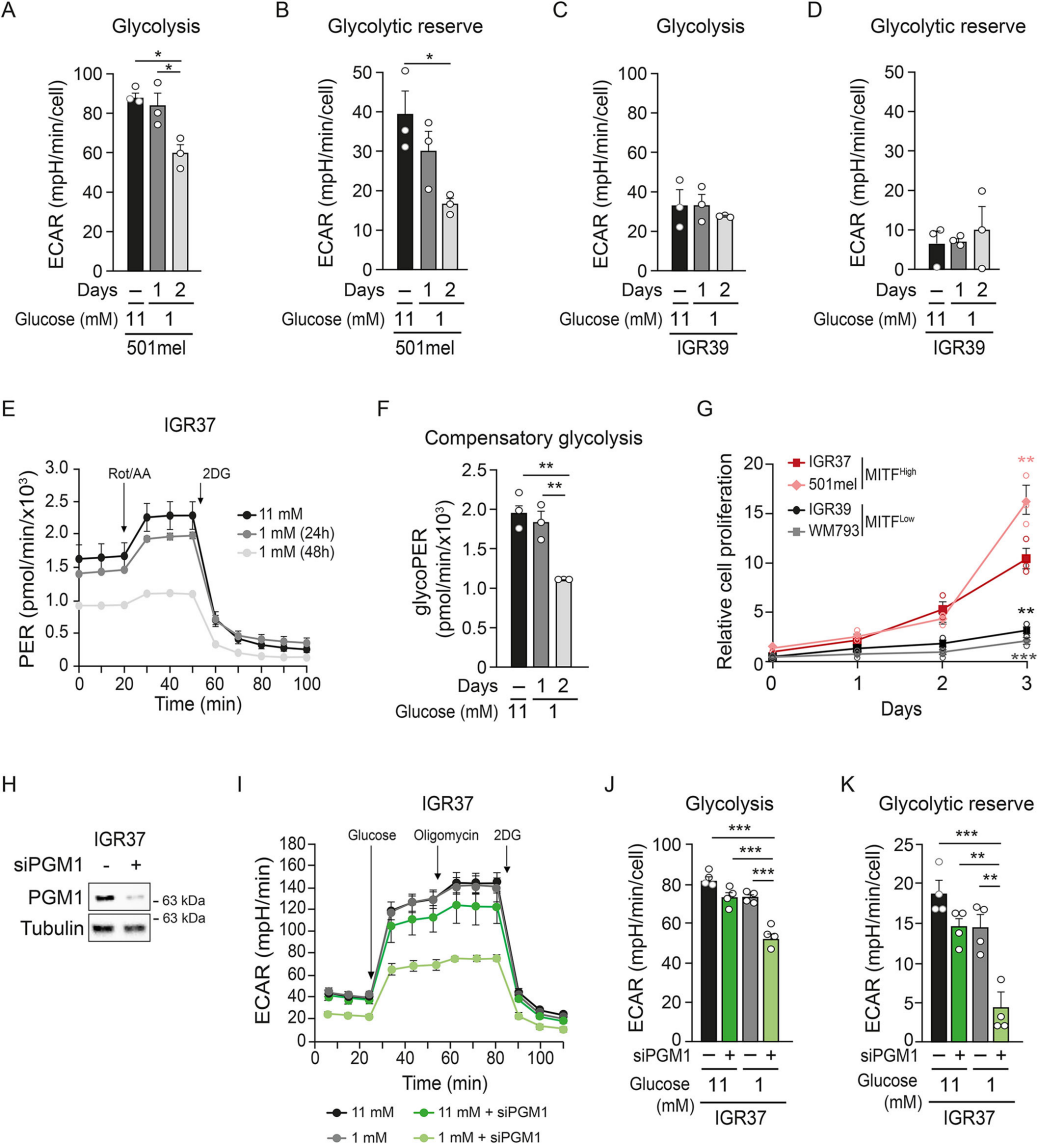
Integrated analysis of glycolytic responses to glucose depletion and PGM1 loss and comparison of proliferation rates for MITF^High^ and MITF^Low^ melanoma cells. (**A**–**D**) Seahorse assessment of basal glycolysis (**A**, **C**) and glycolytic reserve (**B**, **D**) derived from the extracellular acidification rate (ECAR) measurement in 501mel (**A**, **B**) and IGR39 (**C**, **D**) melanoma cells. (**E**) Time course of glycolytic proton efflux rate profile (PER) changes of IGR37 melanoma cells depleted of glucose (from 11 to 1 mM) for 1 and 2 days. Mean ± SEM of *n* = 3 independent experiments. (**F**) Quantification of compensatory glycolysis measurement derived from (**E**); (**G**) Proliferation of MITF^High^ (IGR37, 501mel) and MITF^Low^ (IGR39, WM793) melanoma cells growing in 11 mM glucose for 3 days. (**H**) Western blog analysis of whole cell extracts from IGR37 melanoma cells transfected with control or PGM1-specific siRNAs. Tubulin as loading control. (**I**) Extracellular acidification rate (ECAR) of IGR37 melanoma cells depleted of glucose (1 mM) and/or transfected with PGM1 targeting siRNAs for 24 h. (**J**,** K**) Quantification of glycolytic parameters derived from I under indicated conditions: Basal glycolysis (**J**) and Glycolytic reserve (**K**). Statistical analysis by one-way ANOVA (**A**–**D**, **F**, **G**). *n* = 3 independent experiments (**A**–**D**, **F**, **G**); *n* = 4 independent experiments (**J**, **K**). Data represent mean ± SEM. *P < 0.05; **P < 0.01; ***P < 0.001. Exact *P* values: (**A**) 11 mM vs. 1 mM 2 days, *P* = 0.011; 1 mM 1 day vs. 2 days, *P* = 0,0322. (**B**) 11 mM vs. 1 mM 2 days, *P* = 0.0443. (**F**) 11 mM vs. 1 mM 2 days, *P* = 0.003; 1 mM 1 day vs. 2 days, *P* = 0,0086. (**G**) IGR37 vs. 501mel, *P* = 0,0116, IGR37 vs. IGR39, *P* = 0,0028, IGR37 vs. WM793, *P* = 0,0011; 501mel vs. IGR39, *P* = 0,0001; 501mel vs. WM793, *P* = 0,0001. (**J**) 11 mM vs. 1 mM + siPGM1, *P* = 0.0001; 11 mM + siPGM1 vs. 1 mM + siPGM1, *P* = 0.0001; 1 mM vs. 1 mM + siPGM1, *P* = 0.0001. (**K**) 11 mM vs. 1 mM + siPGM1, *P* = 0.0002; 11 mM + siPGM1 vs. 1 mM + siPGM1, *P* = 0.0038; 1 mM vs. 1 mM + siPGM1, *P* = 0.0038.

In melanoma and other tissues, highly proliferative cells are characterized by a metabolism that is predominantly glycolytic and strongly dependent on glucose (Zhang et al, 2022). Therefore, the correlation between glycogen accumulation and greater proliferative capacity of MITF^High^ cells, suggests first that proliferative phenotype cells take up more glucose than they use for energy production or fabricating biomolecules required for cell division; and second, that the excess glucose is used to make and store glycogen that will act as a metabolic buffer by providing glucose when extracellular glucose is limiting. As such, a capacity to store glycogen will confer an advantage to proliferative cells. We therefore used Seahorse assays to analyze the metabolic plasticity of MITF^High^ cells. Glucose stress tests were used under basal conditions with or without inhibitors of glycogen metabolism. First, the glycolytic rate of IGR37 cells was determined by measuring the ECAR over time under conditions of glucose abundance (11 mM) or limitation (1 mM). The results (Fig. 3A–C) revealed that in the short term (within the first 24 h) glucose depletion only very slightly affected the ECAR, whereas after 2 days of glucose depletion the ECAR was almost abolished (Fig. 3A). Changes in ECAR were quantified to reveal that long term (2 days) glucose depletion strongly reduced glycolysis by 60% (Fig. 3B) and abolished the glycolytic reserve in IGR37 cells (Fig. 3C). Similar changes with milder reductions were recorded in 501mel cells (Fig. EV3A,B). In contrast, MITF^Low^ cells did not respond to glucose depletion with ECAR changes, neither in the short nor in the long term (Fig. EV3C,D). To distinguish how glucose depletion affected the proton efflux rate (PER) due to glycolysis or mitochondrial respiration, a glycolytic rate assay was performed in IGR37 cells. The results revealed that short-term (1 day) glucose depletion does not change the proton efflux rate (PER) (Fig. EV3E) or compensatory glycolysis (Fig. EV3F). However, long-term (2 days) glucose depletion led to a strong reduction of 50% in both glycolysis and compensatory glycolysis (Fig. EV3E,F). The fact that MITF^High^ cells (IGR37 and 501mel) depleted of glucose maintain their maximum glycolytic capacity and reserve for at least the first 24 h, reflects their ability to use glycogen as glucose source under stress conditions; loss of glycogen over longer periods correlates with the loss of glycolytic capacity and reserve after 2 days and with the time needed to record significant reductions in the proliferation rate (as shown in Fig. 2K). Therefore, glycogen mobilization fuels the proliferation capacity of MITF^High^ cell lines, and a lack of glycogen correlates with low proliferation capacity in MITF^Low^ cell lines (Fig. EV3G).

The results suggested that blocking the ability to use glycogen should alter the glycolytic profile and the proliferative capacity of MITF^High^ melanoma cells. We therefore tested this hypothesis using Seahorse analysis. As expected, 24 h of either glucose depletion (from 11 to 1 mM), or treatment with 2-deoxy-D-glucose (2DG), a potent glycolysis inhibitor, did not significantly reduce the proliferation rate of IGR37 cells and only mildly decreased (less than 20%) that of 501mel cells, (Fig. 3D). However, inhibition of glycolysis by 2DG in cells cultured in 1 mM glucose dramatically reduced the proliferation rate in IGR37 by 60% and in 501mel by 40%, unveiling the effect of 2DG in the absence of glycogen reserves. 2DG treatment for 24 h of IGR37 cells cultured with 11 mM glucose moderately decreased their glycolytic profile (Fig. 3E). The ECAR from glycolysis was reduced by 30% (Fig. 3F) without significant alterations in the glycolytic reserve (Fig. 3G). However, when cells were cultured with 1 mM glucose, 2DG treatment led to a strong, 75–80% reduction in ECAR through glycolysis and was almost lost for the glycolytic reserve (Fig. 3F,G). We confirmed these shifts in the glycolytic profile by siRNA-mediated knockdown of PGM1 that blocks both glycogen synthesis and glycogen utilization by inhibiting the reversible conversion between G6P and G1P. PGM1 knockdown (Fig. EV3H) significantly reduced glycolysis in cells cultured with 1 mM glucose but not in the presence of 11 mM glucose, as measured by decreased ECAR (Fig. EV3I,J). The glycolytic reserve was also reduced by more than 50% in PGM1‑depleted cells after 24 h of glucose deprivation (Fig. EV3K).

To target more specifically the ability to use glycogen and study how this would affect the proliferation rate and glycolytic profile of MITF^High^ cells, an inhibitor specific for the enzymes that degrade glycogen (PYG), CP-91149 (Zois and Harris, 2016) was used. Cells cultured with 11 mM or 1 mM glucose were exposed to the PYG inhibitor (PYGi) for 24 h. The proliferation rate of MITF^High^ (IGR37 or 501mel cells) cultured with 11 mM glucose did not change, but was reduced up to 50% in cells cultured with 1 mM glucose (Fig. 3H). The glycolytic profile of IGR37 cells was modified by using CP-91149 in a similar way to 2DG treatment, with the stronger effect in cells cultured with 1 mM glucose (Fig. 3I). As with 2DG, glycolysis was not significantly modified by PYGi in cells cultured with 11 mM glucose but was strongly reduced, more than 50%, in cells cultured in 1 mM glucose according to the changes in ECAR (Fig. 3J). The glycolytic reserve was significantly reduced by >50% in cells cultured with 11 or 1 mM glucose upon treatment with CP-91149 for 24 h (Fig. 3K).

Surprisingly, combined inhibition of glycolysis and glycogen utilization using CP-91149 and 2DG simultaneously, completely blocked the proliferative capacity of MITF^High^ IGR37 and 501mel cells cultured with 11 mM glucose (Fig. 3L), highlighting the importance of glycogen usage under energetic stress.

Thus, blockade of glycogen metabolism in MITF^High^ melanoma cells when cultured under glucose stress (1 mM) strongly reduces the glycolytic capacity by 24 h, with a later reduction in the proliferation rate. The results indicate that glycogen fuels glycolysis when the availability of extracellular glucose becomes limiting, as would occur during tumor expansion.

### Loss of capacity to use glycogen induces a phenotype switch towards invasiveness in melanoma cells

**Figure 4:**
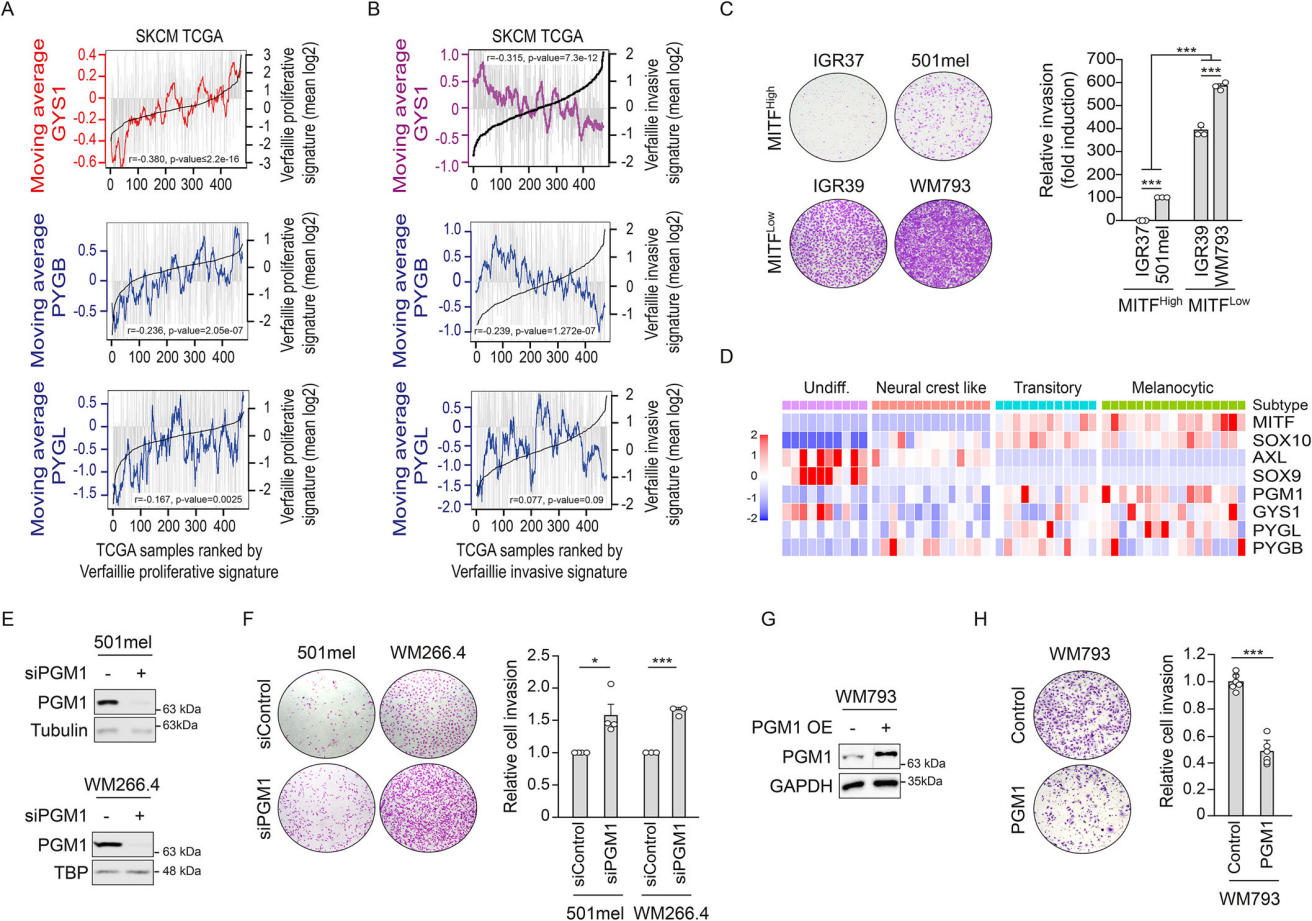
Glycogen metabolism enzymes correlate with proliferative phenotype. (**A**,** B**) GYS1, PYGL, and PYGB mRNA expression (gray bars) in the TCGA melanoma cohort ranked by Verfaillie proliferative (**A**) or invasive (**B**) signature expression (black line). The moving average of each 20-melanoma window is indicated by colored lines. Spearman’s correlation coefficient (R) and *P* value are indicated. (**C**) Matrigel transwell invasion assay of the indicated melanoma cell lines cultured in 11 mM glucose for 24 h. Left: representative images of invading cells stained with crystal violet. Right: quantification from *n* = 3 biological replicates. Data represent mean ± SEM. Statistical analysis by one-way ANOVA. *P < 0.05; **P < 0.01; ***P < 0.001. (**D**) Heatmap showing relative mRNA expression of GYS1, PGM1, PYGB, and PYGL in 53 melanoma cell lines from the CCLE, classified according to Tsoi et al (Tsoi et al, 2018), using MITF, SOX10, AXL, and SOX9 expression as phenotype markers. (**E**, **G**) Western blot analysis of PGM1 protein levels in whole cell extracts from the indicated melanoma cells following PGM1 depletion by siRNA (**E**) or PGM1 overexpression using lentiviral vectors (**G**). Tubulin, TBP, or GAPDH were used as loading controls. (**F**, **H**) Matrigel transwell invasion assay of the indicated melanoma cells transfected with control or PGM1 siRNAs (**F**) or expressing PGM1 overexpression (OE) constructs (**H**) for 24 h. Representative images and quantification are shown. *n* = 3 (**F**); *n* = 6 (**H**). Data represent mean ± SEM. Statistical analysis by unpaired *t*-test. *P < 0.05; **P < 0.01; ***P < 0.001. Exact *P* values: (**C**) IGR37 vs. 501mel, *P* = 0.0001; IGR37 vs. IGR39, *P* = 0.0001; IGR37 vs. WM793, *P* = 0.0001; 501mel vs. IGR39, *P* = 0.0001; 501mel vs. WM793, *P* = 0.0001; IGR39 vs. WM793, *P* = 0.0001. (**F**) 501mel siControl vs. siPGM1, *P* = 0.0167; WM266.4 siControl vs. siPGM1, *P* = 0.0001. (**H**) WM793 Control vs. PGM1 OE, *P* = 0.0001. Source data are available online for this figure.

So far, the results indicate that glucose stress conditions regulate the key enzymes for glycogen metabolism in MITF^High^ melanoma cells to enable them to use glycogen stores to support proliferation. Whether the expression of these enzymes is upregulated in proliferative tumors is unknown. We therefore interrogated the TCGA skin cutaneous melanoma cohort (SKCM) that revealed a strong positive correlation between the Verfaillie proliferative signature gene set (Verfaillie et al, 2015) and *GYS1* mRNA expression (*r* = 0.380; *p* < 2.2 × 10^−16^), (Fig. 4A). Likewise, the Verfaillie proliferative signature also positively correlated with PYGB mRNA expression (*r* = 0.236; *p* = 2.05 × 10^−7^) and PYGL rendered a weak correlation. Consistent with these observations, the Verfaillie invasive gene expression signature significantly and inversely correlated with GYS1 (*r* = 0.315; *p* = 7.3 × 10^−12^) and PYGB (*r* = 0.239; *p* = 1.27 × 10^−7^) but not with PYGL (Fig. 4B). The data imply that the importance of glycogen metabolism is reduced or absent in de-differentiated, invasive, and slow-cycling cells. MITF^Low^ cells have a higher invasive capacity than MITF^High^ cells (Fig. 4C), which is consistent with their absence of glycogen stores and the low levels of key enzymes for glycogen metabolism.

The possible association of glycogen metabolism enzymes with defined melanoma cell phenotypes was explored in a panel of 53 well-characterized melanoma cell lines subdivided into four different phenotypes (Tsoi et al, 2018), with *MITF*, *SOX10*, *SOX9*, and *AXL* used as markers of cell state (Fig. 4D). The results indicate that PYG enzymes for glycogen degradation are present in differentiated melanocytic melanoma phenotypes, which showed high PYGL and low PYGB mRNA levels. PYGL tended to decrease and PYGB to increase in transitory and neural crest phenotypes, and both PYGL and PYGB were absent in undifferentiated phenotypes. For glycogen synthesis, GYS1 mRNA levels were also high in melanocytic phenotypes. The presence of high levels of GYS1 mRNA in undifferentiated phenotypes that lack PGM1 (which provides a substrate for GYS1 reactions) is puzzling. Remarkably, PGM1 mRNA expression, like PYG expression, was largely restricted to MITF^High^ melanocytic and transitory melanoma cell phenotypes that lack expression of AXL, a hallmark of melanoma invasion and therapy resistance (Fig. 4D) (Müller et al, 2014; Konieczkowski et al, 2014). This prompted us to ask whether PGM1 depletion might impact melanoma cell phenotypes.

**Figure EV4:**
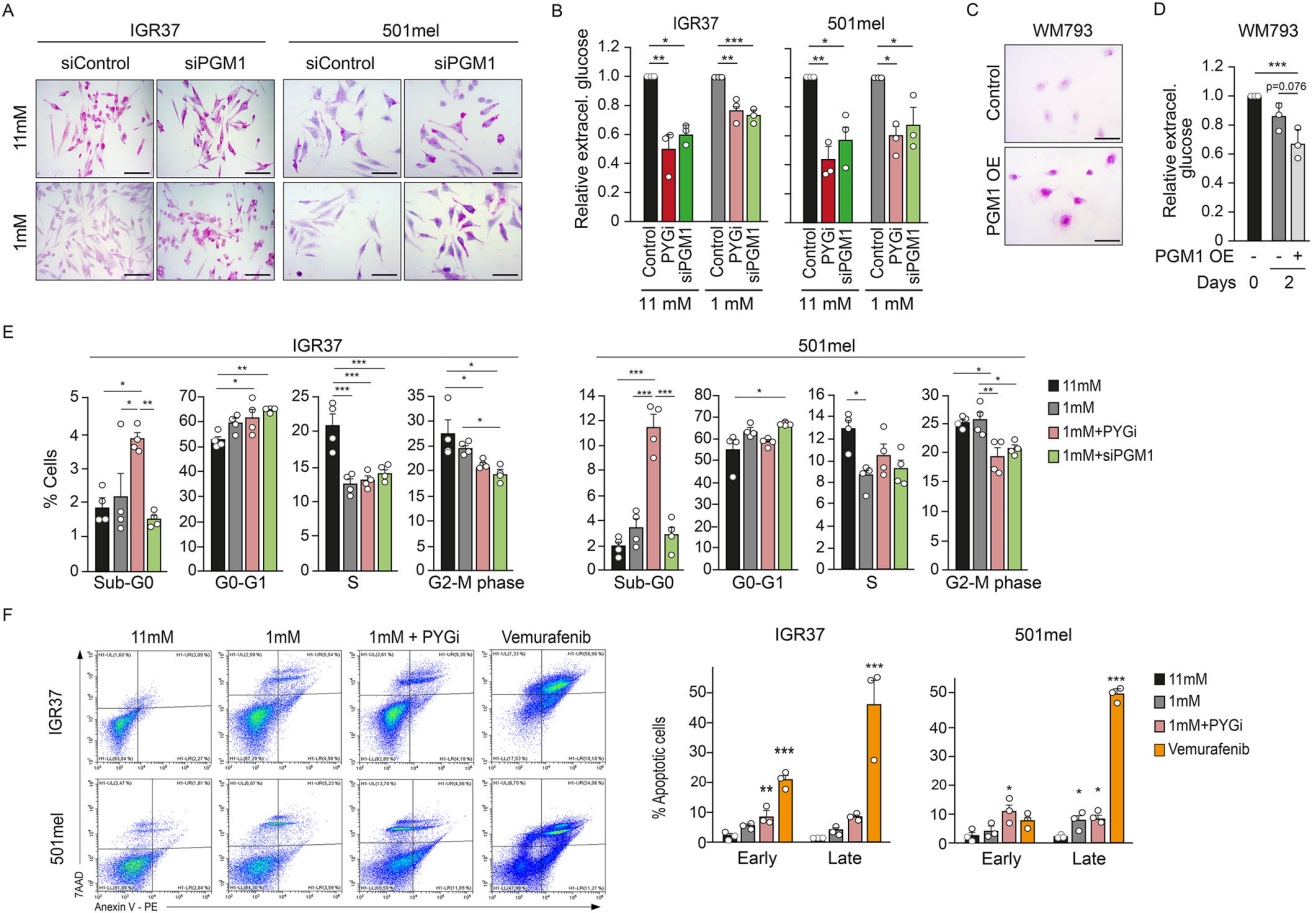
Glycogen dynamics under conditions that limit glucose availability or glycogen degradation in melanoma cells and effects on cell cycle and cell death. (**A**) Changes in glycogen content by MITF^High^ (IGR37, 501mel) melanoma cells upon glucose limitation (11 to 1 mM) or limiting glycogen degradation by specific siRNAs transfection targeting PGM1 (or control) for 24 h. Representative images of PAS staining; scale bars: 25 μm. (**B**) Extracellular glucose depletion by IGR37 and 501mel (MITF^High^) cells upon glucose limitation (from 11 to 1 mM) and/or upon inhibition of glycogen degradation by transfection with PGM1-specific siRNAs or treatment with the glycogen phosphorylase inhibitor CP‑91149 (PYGi, 50 µM) for 24 h. (**C**) Changes in glycogen accumulation by MITF^Low^ WM793 melanoma cells cultured in high glucose and after ectopic lentiviral overexpression (OE) of PGM1 or control DNA, for 24 h; representative images of PAS / PAS-D staining; scale bars: 25 μm. (**D**) Changes in extracellular glucose upon PGM1 overexpression (OE) in WM793 melanoma cells. (**E**) Flow cytometry analysis of MITF^High^ (IGR37, 501mel) melanoma cells after limitation of glucose availability. Cells were depleted of glucose (from 11 to 1 mM, 24 h) or depleted of glucose and of PGM1 by siRNAs or treated with the glycogen phosphorylase inhibitor CP‑91149 (PYGi, 50 µM) for 48 h. Percentages of cells in Sub-Go, Go-G1, S and G2-M phase are shown; (**F**) Flow cytometry analysis of early and late apoptosis. IGR37 and 501mel melanoma cells were depleted of glucose (from 11 to 1 mM) for 24 h or additionally treated with the glycogen phosphorylase inhibitor CP‑91149 (PYGi, 50 µM) for 48 h. Vemurafenib was used as apoptosis positive control. Representative images (left) and quantification (right). Statistical analysis by one-way ANOVA (**B**, **D**, **E**, **F**). *n* = 3 (**B**, **D**, **F**) and *n* = 4 (**E**) biological replicates. Data represent mean ± SEM. *P < 0.05; **P < 0.01; ***P < 0.001. Exact *P* values: (**B**) For IGR37: 11 mM Control vs. PYGi, *P* = 0.0036; Control vs. siPGM1, *P* = 0.0108; 1 mM Control vs. PYGi, *P* = 0.0018; Control vs. siPGM1, *P* = 0.0009. For 501mel: 11 mM Control vs. PYGi, *P* = 0.0029; Control vs. siPGM1, *P* = 0.0107; 1 mM Control vs. PYGi, *P* = 0.0208; Control vs. siPGM1, *P* = 0.0443. (**D**) Control (−) day 0 vs. PGM1 OE (+) day 2, *P* = 0.0006, PGM1 OE (−) vs. PGM1 OE (+) at day 2, *P* = 0.076. (**E**) For IGR37 in Sub-G0 phase: 11 mM vs. 1 mM+PYGi, *P* = 0.0230; 1 mM vs. 1 mM+PYGi, *P* = 0.0306; 1 mM+PYGi vs. 1 mM+siPGM1, *P* = 0.0062. Go-G1 phase: 11 mM vs. 1 mM+PYGi, *P* = 0.0362; 1 mM vs. 1 mM+siPGM1, *P* = 0.0063. S phase: 11 mM vs. 1 mM, *P* = 0.0004; 11 mM vs. 1 mM+PYGi, *P* = 0.0007; 11 mM vs. 1 mM+siPGM1, *P* = 0.0002. G2-M phase: 11 mM vs. 1 mM+PYGi, *P* = 0.0384; 11 mM vs. 1 mM+siPGM1, *P* = 0.0122; 1 mM vs. 1 mM+siPGM1, *P* = 0.0193. For 501mel in Sub-G0 phase: 11 mM vs. 1 mM+PYGi, *P* = 0.0001; 1 mM vs. 1 mM+PYGi, *P* = 0.0001; 1 mM+PYGi vs. 1 mM+siPGM1, *P* = 0.0002. Go-G1 phase: 11 mM vs. 1 mM+siPGM1, *P* = 0.0171. S phase: 11 mM vs. 1 mM, *P* = 0.0340. G2-M phase: 11 mM vs. 1 mM+PYGi, *P* = 0.0121; 1 mM vs. 1 mM+siPGM1, *P* = 0.0320, 1 mM vs. 1 mM+PYGi, *P* = 0.0054. (**F**) For IGR37 Early apoptosis: 11 mM vs. 1 mM+PYGi, *P* = 0.005 and 11 mM vs. Vemurafenib, *P* = 0.0001; Late apoptosis 11 mM vs. Vemurafenib, *P* = 0.0004. In 501mel Early apoptosis: 11 mM vs. 1 mM+PYGi, *P* = 0.0246; Late apoptosis 11 mM vs. 1 mM, *P* = 0.0417; 11 mM vs. 1 mM+PYGi, *P* = 0.0234 and 11 mM vs. Vemurafenib, *P* = 0.0001.

Knockdown of PGM1 using siRNA in MITF^High^ melanoma cells (Fig. 4E) before challenging them with Matrigel invasion assays, revealed more than 50% increased invasiveness in cells depleted of PGM1 (Fig. 4F) despite the abundance of glucose (11 mM). Consistent with an impaired ability to mobilize glycogen stores, PGM1-knockdown cells retained glycogen content even under low-glucose conditions (Fig. EV4A), reflecting their inability to utilize stored glycogen as an alternative energy source when glucose availability is restricted. In line with this, both PGM1-knockdown cells and melanoma cells treated with PYGi (Fig. EV4B) displayed increased glucose uptake, reflecting a compensatory metabolic response to impaired glycogen mobilization. To corroborate these findings through a complementary gain-of-function approach, we overexpressed PGM1 in WM793 cells (Fig. 4G), an invasive MITF^Low^ line characterized by low endogenous PGM1 levels and negligible glycogen accumulation. PGM1 overexpression was sufficient to promote intracellular glycogen accumulation (Fig. EV4C) and significantly reduced invasive capacity in Matrigel assays (Fig. 4H), phenocopying the behavior of MITF^High^ cells with high endogenous PGM1 levels. Although extracellular glucose levels showed a tendency to decrease in PGM1-overexpressing cells, this difference did not reach statistical significance (Fig. EV4D), consistent with a trend toward increased glucose routing into glycogen synthesis. Taken together, these loss- and gain-of-function experiments establish a causal relationship between PGM1 activity, glycogen metabolism and the regulation of melanoma invasiveness, whereby PGM1-driven glycogen storage actively restrains the invasive phenotype.

**Figure 5:**
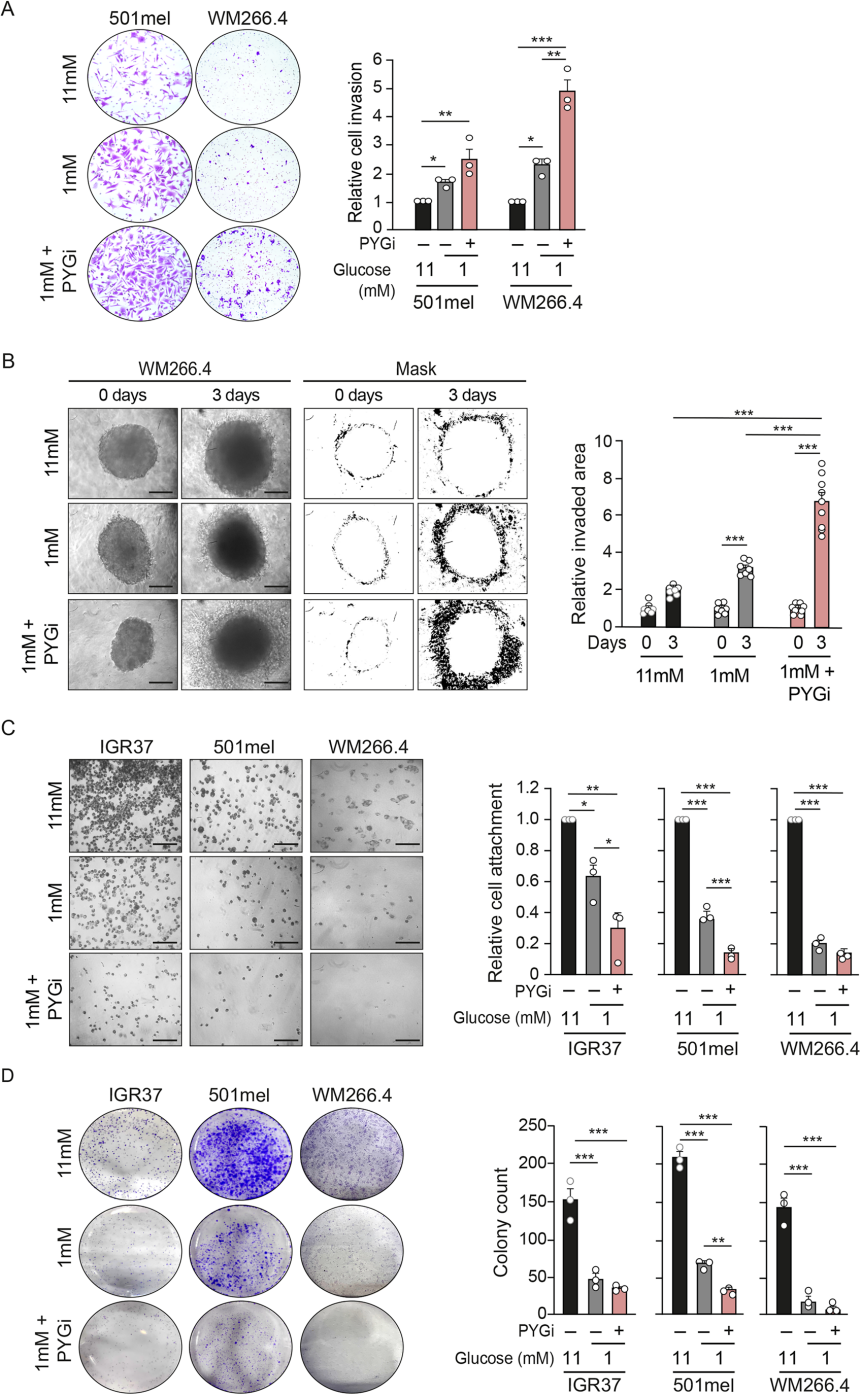
Lack of glycogen mobilization capacity induces a phenotype switch in melanoma cells. (**A**) Matrigel transwell invasion assays of indicated melanoma cells; effect of glucose deprivation (1 mM) alone or together with glycogen phosphorylase inhibition with CP‑91149 (PYGi, 50 µM) for 24 h. Representative images and quantification (*n* = 3). (**B**) Matrigel Invasion assay using spheroids of WM266.4 cells at 0 and 3 days, with corresponding binary masks and quantification. *n* ≥ 7. (**C**) Adhesion assays in indicated melanoma cells cultured under glucose-rich (11 mM) or limited conditions (1 mM) or additionally pretreated with PYGi (50 µM) for 24 h. Representative images and quantification (*n* = 3). (**D**) Colony‑formation assays using the same cell lines and treatments lasting 4 days. Representative images and quantification (*n* = 3). Mean ± SEM of replicates is displayed for panels (**A**–**D**). Statistical analysis by one-way ANOVA. *P < 0.05; **P < 0.01; ***P < 0.001. Exact *P* values: (**A**) For 501mel 11 mM vs. 1 mM, *P* = 0.0135; 11 vs. 1 mM+PYGi, *P* = 0.0077. For WM266.4 11 vs. 1 mM, *P* = 0.0481; 11 vs. 1 mM+PYGi, *P* = 0.0001; 1 mM vs. 1 mM+PYGi, *P* = 0.0012. (**B**) 11 mM vs. 1 mM+PYGi 3days, *P* = 0.0001; 1 mM 0 days vs. 3 days, *P* = 0.0001; 1 mM+PYGi 0 days vs 3days, *P* = 0.0001; 3 days 1 mM vs. 1 mM+PYGi, *P* = 0.0001. (**C**) For IGR37 11 mM vs. 1 mM, *P* = 0.0287; 11 vs. 1 mM+PYGi, *P* = 0.0011; 1 mM vs. 1 mM+PYGi, *P* = 0.043. For 501mel 11 mM vs. 1 mM, *P* = 0.0001; 11 vs. 1 mM+PYGi, *P* = 0.0001; 1 mM vs. 1 mM+PYGi, *P* = 0.002. WM266.4 11 vs. 1 mM, *P* = 0.0001; 11 vs. 1 mM+PYGi, *P* = 0.0001. (**D**) For IGR37 11 vs. 1 mM, *P* = 0.0007; 11 mM vs. 1 mM+PYGi, *P* = 0.0004. For 501mel 11 vs. 1 mM, *P* = 0.0001; 11 vs. 1 mM+PYGi, *P* = 0.0001; 1 mM vs. 1 mM+PYGi, *P* = 0.0058. For WM266.4 11 vs. 1 mM, *P* = 0.0002; 11 vs. 1 mM+PYGi, *P* = 0.0001. Source data are available online for this figure.

Similarly, prolonged glucose depletion or inhibition of glycogen degradation with the glycogen phosphorylase inhibitor CP-94119 (PYGi) increased invasiveness of 501mel and WM266.4 melanoma cells as assessed by Matrigel transwell assay (Fig. 5A) or three-dimensional spheroid invasion assay (Fig. 5B). Under glucose-depleted conditions alone, both 501mel and WM266.4 cells displayed a modest but consistent increase in invasive capacity compared to glucose-replete controls (Fig. 5A). Notably, the combination of glucose depletion and PYGi treatment markedly potentiated this effect, with the most pronounced increase observed in WM266.4 cells (Fig. 5A), a line characterized by a more intermediate phenotype along the proliferative-to-invasive spectrum. Consistent with these findings, WM266.4 spheroids subjected to glucose depletion or PYGi treatment alone showed increased invasive outgrowth compared to untreated controls, and the combined treatment further potentiated invasion in this three-dimensional model (Fig. 5B), corroborating the Matrigel data in a more physiologically relevant setting, and confirming that the inability to access the glycogen metabolic buffer in these settings drives a phenotypic shift toward a more invasive state.

The influence of glucose on adhesion and colony formation, essential steps in distant metastasis were next examined. In line with previous results, glucose depletion to 1 mM significantly reduced adhesion capacity in all MITF^High^ cell lines tested, with reductions of ~30% in IGR37, 60% in 501mel, and 70% in WM266.4 cells (Fig. 5C). Additional inhibition of glycogen phosphorylase with PYGi further reduced adhesion by up to 80% in 501mel and up to 60% in IGR37, suggesting that glycogen mobilization partially compensates for reduced glucose availability to sustain adhesive capacity. Likewise, colony formation was significantly impaired under glucose-limiting conditions across all cell lines tested and was further reduced when glycogen utilization was blocked with PYGi. Quantification of crystal violet staining revealed that glucose depletion alone reduced colony formation by 40–80% depending on the cell line, and that additional PYGi treatment further decreased colony formation to 70–90% of control values (Fig. 5D).

To determine whether the increased invasiveness observed under these conditions was associated with a loss of proliferative potential, cell-cycle distribution was analyzed in MITF^High^ cells (501mel and IGR37) following glucose depletion alone or in combination with pharmacological inhibition of glycogen mobilization by PYGi or genetic depletion of PGM1 by knockdown (Fig. EV4E). Glucose deprivation for 48 h reduced the number of cells in S-phase and promoted the accumulation of cells in G0–G1. PGM1 depletion or PYGi potentiated these effects further, decreasing the G2-M population (Fig. EV4E). Note also a modest increase in cell death (fraction of apoptotic cells (augmented Sub-G0) in glucose‑depleted cells treated with PYGi (Fig. EV4E). Annexin V-PE/7-AAD double staining was performed and confirmed a significant increase in the early apoptotic fraction in IGR37 and 501mel cells subjected to combined glucose depletion and PYGi treatment; vemurafenib treatment in the same cell lines was included as a positive control for cell death (Fig. EV4F).

Together, the results indicate that loss of the capacity to use glycogen reservoirs in MITF^High^ cells favors a phenotypic switch towards an invasive phenotype.

### Glycogen enzymes are transcriptional targets of MITF

**Figure 6:**
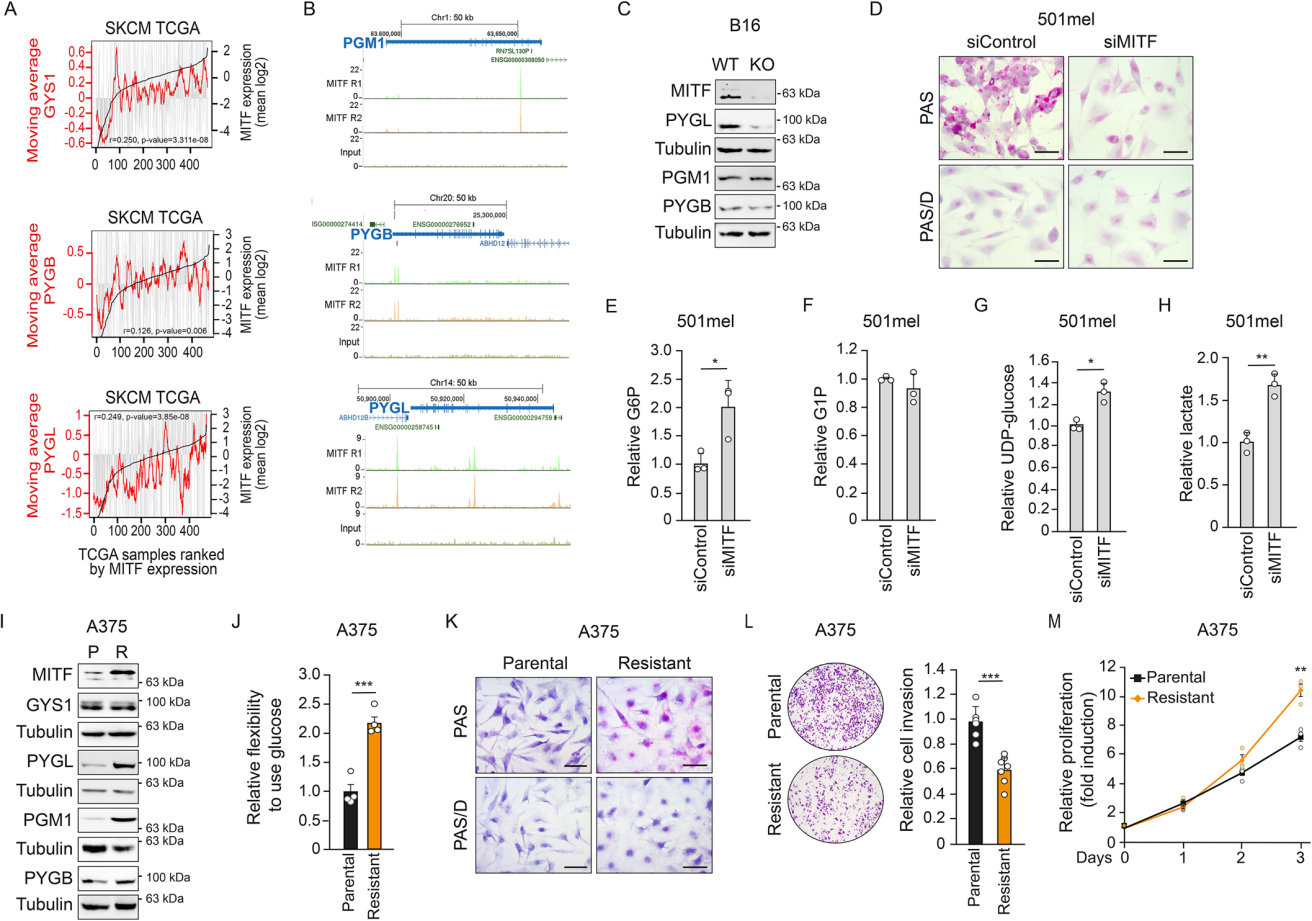
MITF expression controls glycogen mobilization enzymes. (**A**) TCGA melanomas ranked by MITF expression (black lines), with moving average expression of GYS1, PYGB, or PYGL (red line); *p* value and correlation coefficient (*R* Spearman’s rho) are indicated. (**B**) MITF ChIP‑seq tracks showing MITF occupancy at the PGM1, PYGB, and PYGL loci. (**C**) Western blot of whole cell extracts from wild type and MITF‑KO mouse melanoma B16 cells to compare levels of glycogen metabolism enzymes; tubulin as loading control. (**D**) PAS and PAS‑diastase staining of glycogen stores in 501mel cells transfected with control or MITF siRNA. Scale bars: 25 µm. (**E**–**H**) Relative levels of glycogen metabolism intermediates in 501mel cells transfected with control or MITF-specific siRNA: G6P (**E**), G1P (**F**), UDP‑glucose (**G**), and intracellular lactate (**H**). Mean ± SEM of *n* = 3 biological replicates. Data represent mean ± SEM. (**I**) Western blot comparison of MITF and glycogen metabolism enzymes levels in parental (P) and Vemurafenib‑resistant (R) A375 cells; tubulin as loading control. (**J**) Seahorse -assessment of mitochondrial glucose oxidation flexibility in parental and resistant A375 cells. Mean ± SEM of *n* = 4 biological replicates. (**K**) PAS (purple) and PAS‑D staining of glycogen stores in parental and resistant A375 cells. Scale bars: 25 µm. (**L**) Matrigel transwell invasion assays of parental and resistant A375 cells stained with crystal violet. Representative image (left) and Quantification from *n* = 6 replicates (right), mean ± SEM is displayed. (**M**) Proliferation of parental and resistant A375 cells cultured in 11 mM glucose for 3 days. *n* = 3. Data represent mean ± SEM. Statistical analysis (**E**–**H**, **J**, **L**, **M**) by Paired *t*- test; *p < 0.05; **p < 0.01; ***p < 0.001. Exact *P* values: (**E**) siControl vs. siMITF, *P* = 0.0341. (**G**) siControl vs. siMITF, *P* = 0.0161. (**H**) siControl vs. siMITF, *P* = 0.0032. (**J**) Parental vs. resistant, *P* = 0.0003. (**L**) Parental vs. resistant, *P* = 0.0001. (**M**) Parental vs. resistant, *P* = 0.0058. Source data are available online for this figure.

Since glycogen accumulation and elevated expression of glycogen metabolism enzymes, including PGM1 and PYG isoforms, were selectively observed in MITF^High^ cells, the possibility that MITF directly regulates their transcription was explored. This hypothesis was supported by the strong correlation between MITF expression and that of GYS1 (*r* = 0.250; *p* = 3.3 × 10^−8^), PYGB (*r* = 0.126 and *p* = 0.006) or PYGL (*r* = 0.249; *p* = 3.85 × 10^−8^) observed across TCGA melanoma samples (Fig. 6A). To functionally validate this, we interrogated previously published data (Louphrasitthiphol et al, 2020) on biological replicates of MITF chromatin immunoprecipitation-sequencing (ChIP-seq). The data set revealed MITF binding to the *PYGM1*, *PYGB*, and *PYGL* loci (Fig. 6B), raising the possibility that these genes are directly regulated by MITF. The protein levels of PGM1 and PYG isoforms were next assessed in previously published MITF-knockout B16 cells (Sánchez-del-Campo et al, 2021; Dias et al, 2025). MITF ablation resulted in strong downregulation of PYGL protein, while PYGB and PGM1 levels remained unchanged (Fig. 6C), indicating that MITF selectively controls PYGL expression among the glycogen metabolism enzymes examined.

To extend these findings to a complementary loss-of-function approach in human melanoma cells, MITF was transiently silenced by siRNA in 501mel cells (Fig. 6D,H). MITF knockdown resulted in a significant reduction in intracellular glycogen content, as evidenced by decreased PAS staining intensity (Fig. 6D), indicating that MITF actively promotes glycogen accumulation in melanoma cells. This was accompanied by metabolic changes compatible with reduced glycogen utilization, including elevated intracellular G6P levels and an increased G6P/G1P ratio (Fig. 6E,F), accumulation of UDP-glucose (Fig. 6G), and increased lactate production from glycolysis (Fig. 6H), suggesting a shift away from glycogen storage upon MITF loss. Notably, the elevated G6P/G1P ratio observed in MITF-silenced 501mel cells recapitulates the metabolic profile characteristic of MITF^Low^ cells (Fig. 2B).

Beyond steady-state conditions, we next asked whether the MITF–glycogen axis is dynamically remodeled in a clinically relevant context of acquired drug resistance. To address this, we evaluated how glycogen enzyme levels change during resistance acquisition using the A375 cell line, which expresses intermediate levels of MITF and is widely used as a model for the response to BRAF-targeted therapy. Cells were selected for long-term resistance to vemurafenib, a treatment known to induce phenotypic plasticity and shifts along the proliferative-to-invasive axis in melanoma. Upon resistance acquisition, both PYGL and PGM1 protein levels were markedly upregulated (Fig. 6I), in concert with an increase in MITF expression, consistent with a phenotypic transition toward a more differentiated state. This transcriptional and enzymatic reprogramming was accompanied by a metabolic shift toward greater flexibility in glucose utilization, as assessed by Seahorse extracellular flux analysis (Fig. 6J), increased intracellular glycogen accumulation (Fig. 6K), a significant reduction in invasive capacity compared with parental cells (Fig. 6L) and an increase in proliferative capacity (Fig. 6M), collectively indicating that resistance acquisition drives cells toward a MITF^High^-like state characterized by enhanced glycogen metabolism.

### PGM1- and PYGB-dependent glycogen mobilization during human melanoma progression has prognostic value

**Figure 7:**
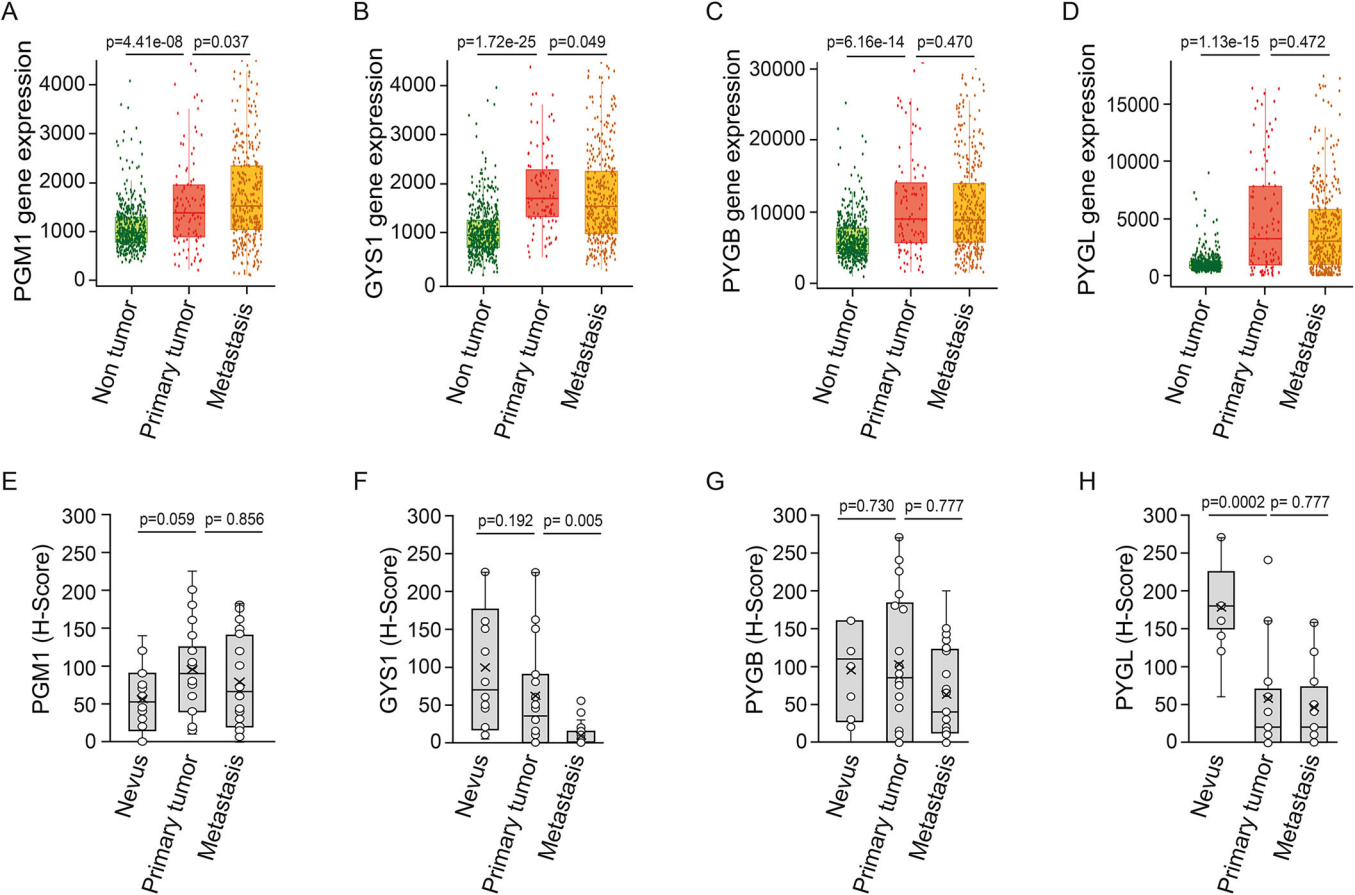
Comparison of glycogen mobilization enzyme dynamics at the mRNA level (nontumor to primary tumor to metastasis) and protein level (nevi to primary tumor to metastasis). (**A**–**D**) TNM plot analysis of mRNA levels of PGM1 (**A**), GYS1 (**B**), PYGB (**C**), or PYGL (**D**) in 377 samples of non-cancer tissue (normal) versus 211 melanoma tissue (tumor) and 111 metastatic tissues (metastatic). Data were collected from the TCGA database. Expression is shown as transcripts per million (TPM) in log2. Samples were compared with the Mann–Whitney *U*-test. (**E**–**H**) Histoscore (H-Score) profile for PGM1 (**E**), GYS1 (**F**), PYGB (**G**), and PYGL (**H**) content comparing nevus (*n* = 14 in (**E**–**G**) and *n* = 12 in **H**), primary melanoma (*n* = 22 in (**E**), *n* = 23 in (**F**), *n* = 26 in (**G**), and *n* = 21 in (**H**)) and metastatic (*n* = 23 in (**E**, **F**); *n* = 25 in (**G**) and *n* = 24 in (**H**) human samples as in Fig. 1B. Statistical analysis by Mann–Whitney *U*-test. *p < 0.05, **p < 0.01, ***p < 0.001. (**A**–**H**) Box plots show median (50%) with lower (25%) and upper (75%) quartiles; whiskers represent minimum and maximum values. Exact *P* values: (**A**–**D**) Nontumor vs. primary tumor: PGM1, *P* = 4.41 ×  10⁻⁸; GYS1, *P* = 1.72 × 10⁻²⁵; PYGB, *P* = 6.16 × 10^−^¹⁴; PYGL, *P* = 1.13 × 10^−^¹⁵. Primary tumor vs. metastasis: PGM1, *P* = 0.037; GYS1, *P* = 0.049; PYGB, *P* = 0.470; PYGL, *P* = 0.472. (**E**–**H**) Nevus vs. primary tumor: PGM1, *P* = 0.059; GYS1, *P* = 0.192; PYGB, *P* = 0.730; PYGL, *P* = 0.0002. Primary tumor vs. metastasis: PGM1, *P* = 0.856; GYS1, *P* = 0.005; PYGB, *P* = 0.777; PYGL, *P* = 0.777. Source data are available online for this figure.

In many cancer types other than melanoma, high tumor expression of enzymes related to glycogen metabolism is associated with worse patient outcomes (de Heer et al, 2023), but the underlying mechanisms remain unclear. To understand whether and how the gene expression of enzymes required for glycogen synthesis (*GYS1* and *PGM1*) or degradation (*PYGB* and *PYGL*) is associated with patient outcome, we explored the TCGA database with 571 human cutaneous melanoma samples matched with non-cancerous tissue. Differential gene expression analysis in Tumor, Normal, and Metastatic tissues (TNMplot) analysis revealed mRNA increases from normal skin to tumor tissue for *PGM1* (Fig. 7A), *GYS1* (Fig. 7B), *PYGB* (Fig. 7C), and *PYGL* (Fig. 7D) that were significant in all cases (*p* < 0.001); despite the under-representation of melanocytes in normal skin, the data implies that expression of genes for glycogen metabolism is important in primary tumors. Comparison between primary tumors and metastases revealed significant (*P* < 0.05) changes in the expression of enzymes involved in glycogen synthesis, with increased *PGM1* (Fig. 7A) and decreased *GYS1* (Fig. 7B) expression, whereas no significant changes were observed in the expression of genes encoding glycogen-degrading enzymes *PYGB* or *PYGL* (Fig. 7C,D). The data were compatible with an increased need to express enzymes for glycogen metabolism early in tumor formation (from non-malignant to tumor) and the progressive loss of glycogen metabolism towards metastasis. The tendency to decrease and the ample variation in metastasis for gene expression of glycogen metabolism enzymes is indicative of a heterogeneity of adaptive metabolic responses that may vary with the new niche.

The multiplicity of mechanisms that regulate enzyme activity frequently leads to discrepancies between mRNA and protein levels. We therefore performed immunohistochemical analysis to assess protein expression of glycogen metabolism enzymes in a tissue microarray (TMA) comprising pre-malignant human nevi (*n* = 14), primary melanoma (*n* = 27) and metastatic samples (*n* = 28) obtained from the Biobank of Hospital Clínico San Carlos (Table 1). Comparison of nevi with primary melanoma samples showed a trend toward increased PGM1 protein levels that did not reach statistical significance (Fig. 7E). It is important to note that the mRNA data derived from TCGA compares non‑tumor tissue with primary tumor samples, whereas the protein analyses in our TMA contrast nevi with primary melanoma. This difference in the reference baseline may also contribute to the observed discrepancies between mRNA and protein expression patterns. From primary melanoma to metastasis, PGM1 protein levels showed a non-significant tendency to decrease, contrasting with the upward trend observed at the mRNA level, suggesting that post-transcriptional regulatory mechanisms may contribute to refine PGM1 protein levels in this transition. A similar protein/mRNA discrepancy was observed for GYS1, whose protein levels tended to decrease from nevi to primary tumor, though this difference did not reach statistical significance (Fig. 7F). This contrasted with the significant increase recorded at the mRNA level between nontumor and primary tumor (Fig. 7B). The difference between mRNA and protein results may reflect the limited sample size available for protein detection or the fact protein levels are being compared between nevi and primary tumor, whereas mRNA was compared between nontumor and primary tumor. Importantly, a strong and statistically significant decrease in GYS1 protein levels was detected between primary melanoma and metastases (*p* = 0.005), suggesting that downregulation of glycogen synthesis capacity may be a feature of metastatic progression, consistent with the notion that active glycogen storage restrains invasive dissemination.

**Figure EV5:**
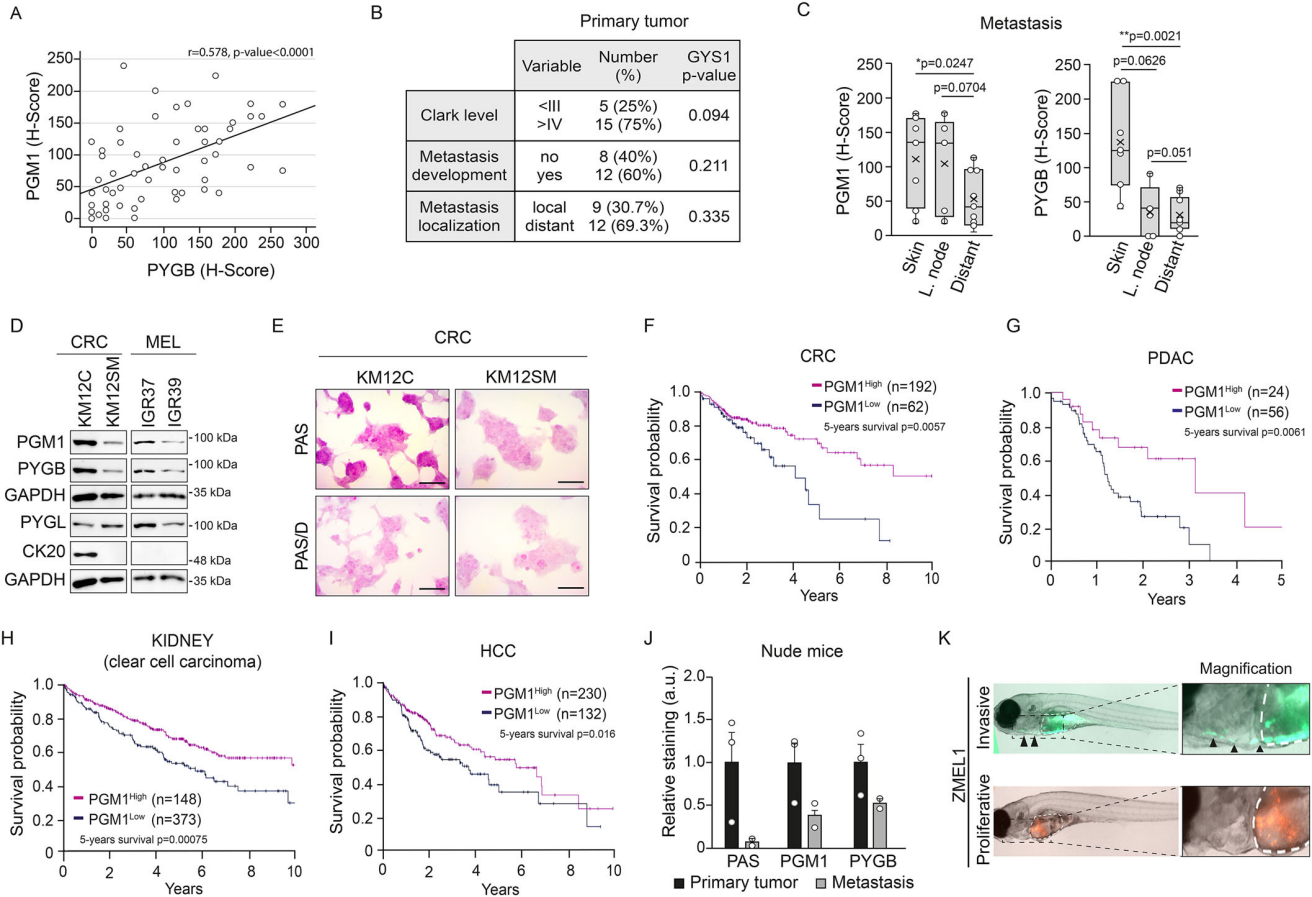
Relevance of glycogen mobilization enzymes across cancer types. (**A**) Spearman rank correlation between PGM1 and PYGB protein levels in human melanoma samples. Spearman’s rho (*R*) and *P* value are indicated. *p < 0.05, **p < 0.01, ***p < 0.001. (**B**) Association analysis between GYS1 H-score and clinicopathological characteristics of patients listed in Table 1. Statistical analysis by the Mann–Whitney *U*-test. Significance threshold set at *P* < 0.01. Exact *P* values for GYS1: Clark level, *P* = 0.094; Metastasis development, *P* = 0.211; metastasis localization, *P* = 0.335. (**C**) H-score analysis of PGM1 and PYGB protein levels in melanoma metastasis samples (Table 1) stratified by distance from the primary tumor (skin, lymph node, or distant). Box plots show median (50%) with lower (25%) and upper (75%) quartiles; whiskers represent minimum and maximum values. Statistical analysis by the Mann–Whitney *U*-test. *P < 0.05; **P < 0.01; ***P < 0.001. Exact *P* values: Skin vs. distant: PGM1, *P* = 0.0247; PYGB, *P* = 0.0021. Lymph node vs. distant: PGM1, *P* = 0.0704; PYGB, *P* = 0.0051. Skin vs. lymph node: PYGB, *P* = 0.0626. (**D**) Western blot analysis of whole cell extracts; levels of glycogen-degrading enzymes (PGM1, PYGB, and PYGL) in proliferative versus metastatic phenotypes of colorectal cancer (KM12C and KM12SM) or melanoma (IGR37 vs IGR39) cells. GAPDH as loading control. (**E**) Glycogen content in proliferative KM12C and invasive KM12SM colorectal cancer cell lines revealed by PAS/PAS-D staining. Representative images; scale bars: 25 μm. (**F**–**I**) Kaplan–Meier curves displaying the estimated survival probability performed using the best cut-off point and data from The Human Protein Atlas for the following cohorts: colorectal cancer (**F**), (CRC): *n* = 254 of which PGM1^High^ (*n* = 192), PGM1^Low^ (*n* = 62), *P* = 0.0057; pancreatic ductal adenocarcinoma (**G**), (PDAC): *n* = 80, PGM1^High^ (*n* = 56), *P* = 0.0061; PGM1^Low^ (*n* = 24); kidney Clear cell carcinoma (**H**), *n* = 521, PGM1^High^ (*n* = 148); PGM1^Low^ (*n* = 373), *P* = 0.00075; and hepatocellular carcinoma (**I**) (HCC): *n* = 362, PGM1^High^ (*n* = 132),; PGM1^Low^ (*n* = 230), *P* = 0.016. *p < 0.05, **p < 0.01, ***p < 0.001 (**J**) Quantification of IHC staining of mouse tumor samples: primary and metastasis. The data corresponds to the histoscore profile of glycogen content (PAS), PGM1 and PYGB glycogen-degrading enzymes. (**K**) Larval zebrafish 3 days post-transplant (dpt) of invasive (green) or proliferative (orange) ZMEL1 cells into the yolk sac, representative images. The dotted line indicates the original transplantation site. Unfilled arrowheads indicate instances of dissemination to distal tissue.

For the glycogen-degrading enzymes, PYGB did not show significant differences between nevi and primary melanoma or metastases (Fig. 7G). However, PYGL (Fig. 7H) revealed a very significant (*p* = 0.0002) decrease in protein levels from nevi to primary melanoma, which contrasted with the increased mRNA expression presented in Fig. 7D. The transition from primary tumor to metastasis was accompanied by a tendency toward decreased PYGL mRNA and protein levels, though neither reached statistical significance, suggesting again that glycogen metabolism is non-essential for metastases. Interestingly, the levels of PGM1 and PYGB proteins showed a very significant positive correlation (*r* = 0.578, *p* < 0.0001) (Fig. EV5A). Note the dual role of PGM1, which may participate in glycogen synthesis by converting G6P to G1P, or in glycogen degradation by converting G1P into G6P.

**Figure 8:**
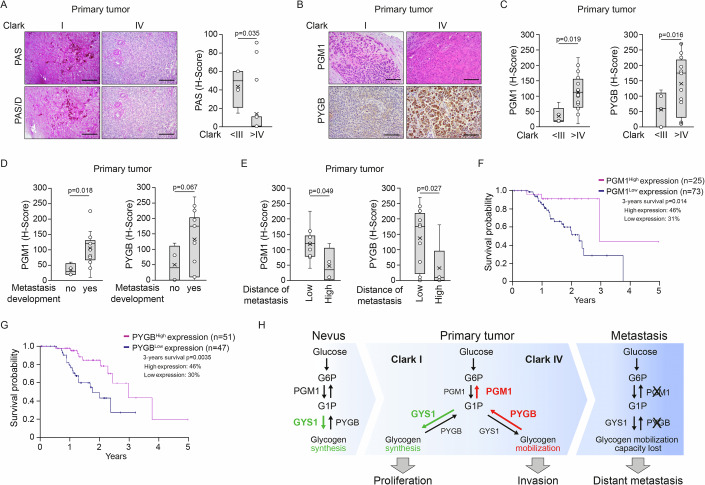
Clinical relevance of PYGB and PGM1 levels in human melanoma samples. (**A**) Glycogen content assessed by PAS staining (purple) in human melanoma biopsies stratified by Clark level (I or IV). Diastase digestion (PAS/D) confirms staining specificity. Representative images (left); scale bars: 50 µm. Right: quantification of glycogen content as Histoscore (H-score) in melanomas with low (≤III) or high (≥IV) Clark level. (**B**) Immunostaining (brown) for PGM1 or PYGB on human melanoma samples (Clark I or IV level as in **A**) with hematoxylin counterstaining for nuclei (dark blue) and cytoplasm (light blue). Representative micrographs; scale bars: 50 μm. (**C**–**E**) Quantification of PGM1 or PYGB content to compare: primary tumors with low versus high Clark level (C), melanomas that developed metastasis or did not (**D**) and melanomas that developed metastases at low or high distance from primary tumor (**E**). Box plots show median (50%) with lower (25%) and upper (75%) quartiles; whiskers represent minimum and maximum values. Statistical analysis by the Mann–Whitney *U*-test. *P < 0.05; **P < 0.01; ***P < 0.001. Exact *P* values: (**A**, **C**) Clark ≤ III vs. ≥ IV: PAS, *P* = 0.035; PGM1, *P* = 0.019; PYGB, *P* = 0.016. (**D**) Metastasis development (no vs. yes): PGM1, *P* = 0.018; PYGB, *P* = 0.067. (**E**) Metastasis distance (low vs. high): PGM1, *P* = 0.049; PYGB, *P* = 0.027. (**F**, **G**) Kaplan–Meier curves displaying the estimated survival probability for melanoma patients performed using the best cut-off point and data from 102 melanoma patients from The Human Protein Atlas; PGM1^High^ (*n* = 25) and PGM1^Low^ (*n* = 73) in (**F**), *P* = 0.014; PYGB^High^ (*n* = 51) and PYGB^Low^ (*n* = 47) in (**G**), *P* = 0.0035. (**H**) Schematic overview of glycogen metabolism dynamics during melanoma progression. Nevus and primary melanomas (Clark I) exhibit high glycogen content, which correlates with higher GYS1 levels in proliferative stages. Primary tumors that go on to develop metastases exhibit higher levels of glycogen mobilization enzymes: PGM1 and PYGB than those that do not develop metastases; PGM1 and PYGB activity drives glycogen depletion and associated loss of metabolic buffering to suppress invasion. Melanomas that develop distant metastases (more aggressive) and their metastases (from lung, brain or liver) exhibit lower levels of PGM1 and PYGB and have a worse prognosis. Glycogen synthesis is presented with green-labeled enzymes and arrows, and glycogen degradation by red-labeled enzymes and arrows. Proliferative, invasive, and metastatic phenotypes (bottom) are linked to glycogen synthesis, breakdown, or loss of glycogen mobilization capacity. Source data are available online for this figure.

Globally, the data suggested that the capacity to store and mobilize glycogen plays an important role in the early stages of melanoma, in which MITF^High^ melanoma cells may use glycogen as an energy source to proliferate, and this capacity loses importance as the disease progresses. To explore this, we evaluated the association between the expression levels of GYS1, PGM1 and PYGB and clinicopathological features of disease progression, including invasiveness through the skin as measured by Clark level, establishment of metastases and distance of metastasis from the primary tumor. GYS1 levels were not associated with any of these features (Fig. EV5B). In primary tumors, those with higher metastatic capacity (Clark IV) exhibited significantly lower glycogen levels (Fig. 8A) and significantly elevated PGM1 and PYGB expression (Fig. 8B,C), indicative of enhanced glycogen mobilization capacity. Rather than reflecting glycogen accumulation, this pattern suggests that glycogen stores are actively depleted through increased catabolism, highlighting the potential importance of glycogen utilization for melanoma progression. The capacity to establish metastases was positively associated with increased expression of PGM1 and showed a trend toward higher PYGB expression (Fig. 8D), consistent with a metabolic advantage conferred by glycogen catabolism during early metastatic dissemination.

Interestingly, primary tumors that gave rise to metastases relatively close to the primary lesion exhibited higher PGM1 and PYGB levels than those associated with distant metastases in lung, liver or brain (Fig. 8E), suggesting that the glycogen-mobilizing phenotype may be particularly relevant for local invasion and early dissemination, whereas more aggressive tumors capable of distant organ colonization may rely on alternative metabolic strategies. Supporting these observations, Kaplan–Meier analysis of melanoma patient samples (Fig. 8F,G) revealed that tumors expressing high levels of PGM1 and PYGB were associated with significantly improved patient survival, consistent with the notion that the capacity to mobilize glycogen correlates with a less aggressive disease course. This is in line with the in vitro results, where genetic depletion of PGM1, which impairs glycogen mobilization, leads to increased invasiveness (Fig. 4E,F). Comparable trends were observed in metastatic samples, where skin metastases, which are less aggressive and associated with a more favorable prognosis, exhibited higher PGM1 and PYGB levels than visceral metastases in the liver or lung (Fig. EV5C). Our results suggest that more distantly located metastases express lower levels of glycogen mobilization enzymes and display reduced glycogen stores (Fig. 1A), pointing to a progressive loss of glycogen metabolic capacity with increasing metastatic distance.

To assess whether these findings extend beyond melanoma, we examined glycogen metabolism in an in vivo-derived colorectal cancer metastasis model. The parental KM12C colorectal carcinoma line and its spontaneous hepatic metastasis-derived subline KM12SM (Morikawa et al, 1988b, 1988a) were compared. KM12SM displays a more aggressive phenotype and higher metastatic tropism toward the liver than the parental line. Western blot analysis revealed that KM12C cells express high levels of glycogen metabolism enzymes, including PGM1 and PYG isoforms, whereas KM12SM cells showed a marked reduction in these proteins (Fig. EV5D, left panels), mirroring the pattern observed between MITF^High^ IGR37 and MITF^Low^ IGR39 melanoma cells (Fig. EV5D, right panels). Consistent with this enzymatic profile, KM12C cells accumulated substantial intracellular glycogen as evidenced by PAS staining, while KM12SM cells showed negligible glycogen content, with diastase treatment confirming staining specificity (Fig. EV5E). Together, these data indicate that the inverse relationship between glycogen mobilization capacity and metastatic aggressiveness is not restricted to melanoma but is recapitulated in a colorectal cancer progression model. To further explore the clinical relevance of these findings across tumor types, Kaplan–Meier survival analyses were performed in independent cohorts of colorectal cancer (CRC), pancreatic ductal adenocarcinoma (PDAC), kidney clear cell carcinoma and hepatocellular carcinoma (HCC) (Fig. EV5F–I). Consistent with the melanoma data, high PGM1 expression was associated with improved survival probability across all four cancer types, suggesting that the prognostic value of glycogen mobilization capacity may extend to multiple tumor contexts.

Taken together, these results support a model in which glycogen metabolism plays a dual role in melanoma progression: it sustains proliferative capacity at early stages while actively suppressing invasiveness, such that loss of glycogen stores, or impaired glycogen mobilization, facilitates a phenotypic switch from proliferation to invasion (Fig. 8H). Beyond its mechanistic relevance, these findings highlight the potential prognostic value of glycogen metabolic status in melanoma, which could be evaluated in clinical samples using the straightforward combination of PAS staining for glycogen content and immunohistochemistry for PGM1 and PYGB expression.

### Inhibition of glycogen phosphorylase enhances tumor cell invasion in vivo

Having established that glycogen mobilization suppresses invasiveness at the cellular level and correlates with disease progression in patient samples, we next sought to validate these findings using complementary in vivo models and to assess the functional consequences of pharmacological glycogen phosphorylase inhibition on tumor invasive behavior.

**Figure 9:**
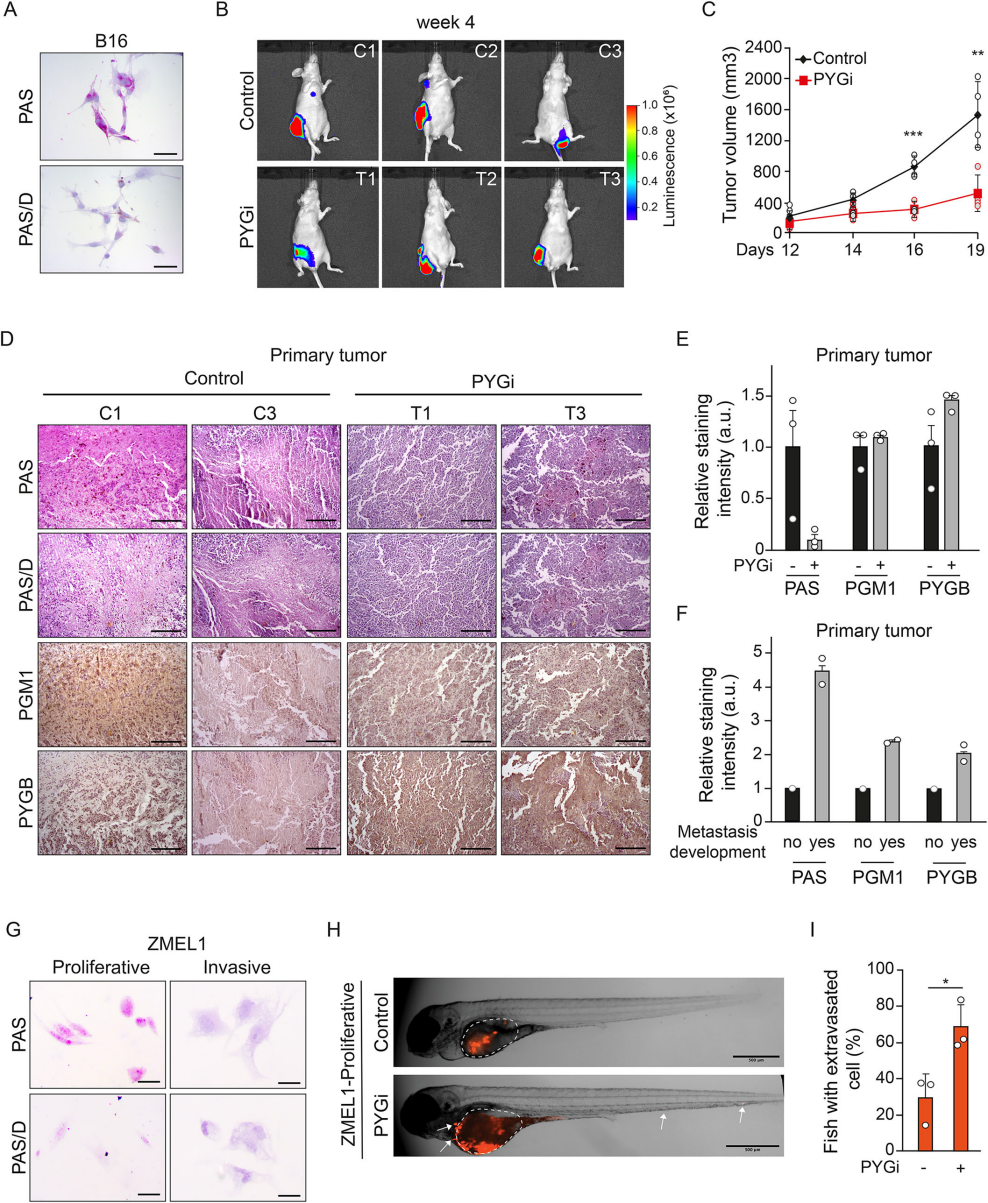
Glycogen phosphorylase inhibition promotes tumor cell invasion in vivo. (**A**) Glycogen stores in B16-F10 melanoma cells revealed by PAS staining (purple), diastase (PAS-D) treatment shows specificity for glycogen staining. Representative images, scale bars: 25 μm. (**B**) Representative bioluminescence IVIS images of control (C1–C3) or treated with the PYGi CP-91149 (50 mg/kg) (T1–T3) mice, 4 weeks after subcutaneous injection of B16-F10 luciferase cells (*n* = 4 mice/group). Firefly luciferin (120 mg/kg) was injected intraperitoneally. (**C**) Tumor growth was quantified by measuring the volume with a digital caliper in mice from (**B**). Data represent mean ± SEM. Statistical analysis by one-way ANOVA. **P < 0.01; ***P < 0.001. Control vs. PYGi: day 16, *P* = 0.0007; day 19, *P* = 0.0056. (**D**) Immunohistochemical analysis of glycogen content and glycogen-mobilizing enzymes in the primary tumor of mice treated with vehicle (control) or PYGi. Glycogen content was stained with Periodic Acid-Shiff (PAS) alone (purple) or followed by diastase (PAS/D) digestion, as in previous figures to prove specificity (top panels); immunohistochemistry of paraffin-embedded sections stained with PGM1 and PYGB antibodies (brown) (bottom panels). Representative images; scale bars = 50 µm. (**E**,** F**) Quantification of IHC staining from primary tumor samples as described in (**D**). *n* = 3 per group. Data represent mean ± SEM. (**G**) Glycogen stores in proliferative and invasive ZMEL1 zebrafish melanoma cells revealed by PAS and PAS/D staining. Representative images; scale bars: 25 µm. (**H**) Representative image of a larval zebrafish 3 days post-transplant (dpt) of proliferative cells treated with 0.01% DMSO (Control) or 1 μM CP-91149 (PYGi) into the yolk sac. The dotted line indicates the original transplantation site. Unfilled arrowheads indicate dissemination to distal tissue. Scale bars: 500 µm. (**I**) Quantification of proliferative (PRO) cell transplants. Each data point represents a replicate of *n* = 6 to *n* = 11 fish per group. Data represent mean ± SEM. Statistical analysis by unpaired *t*-test. *P < 0.05. PYGi (−) vs. (+), *P* = 0.0202. Source data are available online for this figure.

To this end, B16 melanoma cells, which display a proliferative phenotype and accumulate intracellular glycogen, as confirmed by positive PAS staining, abolished upon diastase pretreatment (Fig. 9A), were implanted subcutaneously into athymic nude mice. Animals were treated with the glycogen phosphorylase inhibitor CP-91149 (PYGi) or vehicle once tumors reached a palpable size. Vehicle-treated mice developed significantly larger primary tumors compared to PYGi-treated animals, as demonstrated by IVIS bioluminescence imaging (Fig. 9B) and quantified by serial caliper measurements (Fig. 9C). Strikingly, vehicle-treated mice developed overt metastases, whereas PYGi-treated animals failed to do so (Fig. 9B).

Histological analysis of primary tumor sections from these mice revealed a degree of tumoral heterogeneity comparable to that observed in human samples, characterized by variable glycogen content and corresponding patterns of immunohistochemical staining for PGM1 and PYGB (Fig. 9D, mice C1 and C3). PYGi treatment led to a decrease in PAS staining intensity (Fig. 9D, mice T1 and T3), reminiscent of the pattern observed in human Clark stage IV primary tumors (Fig. 8A). Immunohistochemical staining for PGM1 and PYGB showed increased expression of PYGB in PYGi-treated animals (Fig. 9D,E), indicating upregulation in response to treatment, possibly reflecting a compensatory attempt to access depleted glycogen stores under the high energetic demand imposed by PYGi. Notably, this combination of low glycogen content and elevated PYGB expression closely parallels the metabolic profile of highly invasive Clark stage IV human tumors (Fig. 8A–C), further supporting the translational relevance of this model.

Stratification of untreated primary tumors according to metastatic outcome allowed further exploration of the relationship between glycogen metabolism and metastatic progression within this model. Tumors from mice that developed metastases exhibited significantly higher PAS staining intensity (Fig. 9D, C1) alongside elevated PGM1 and PYGB expression compared with non-metastatic tumors (Fig. 9D, C3), as confirmed by IHC quantification (Fig. 9F), reinforcing our findings in human primary melanoma and the notion that active glycogen metabolism correlates with metastatic capacity. Strikingly, analysis of matched lung metastases revealed markedly lower PAS staining and reduced PGM1 and PYGB expression compared with their corresponding primary tumors (Fig. EV5J), recapitulating the pattern observed in human metastatic samples (Fig. 8D) and suggesting that glycogen mobilization capacity is progressively lost as tumor cells colonize distant organs.

While the murine model allowed us to assess the overall impact of PYGi on tumor growth and metastatic outcome, the early cellular events underlying tumor cell dissemination are difficult to resolve in vivo in mice, as the failure of PYGi-treated mice to exhibit metastasis could arise for several reasons. We therefore turned to a zebrafish larval transplantation model, which, owing to the optical transparency of *casper* larvae, enables real-time visualization and quantification of the initial steps of tumor cell invasion at single-cell resolution. First, we confirmed that zebrafish melanoma ZMEL proliferative phenotype cells accumulate intracellular glycogen, as evidenced by positive PAS staining, whereas ZMEL invasive phenotype cells displayed markedly reduced glycogen content, with diastase treatment confirming staining specificity (Fig. 9G). This is consistent with the metabolic differences observed between MITF^High^ and MITF^Low^ human melanoma cells and supports the conservation of the glycogen-invasiveness axis across species. Fluorescently labeled ZMEL proliferative (orange) and invasive (green) cells were microinjected into the yolk sac of 2-day post-fertilization *casper* zebrafish larvae and treated with PYGi or vehicle before imaging 3 days later. ZMEL invasive cells showed significantly higher dissemination beyond the yolk sac than proliferative cells in vehicle-treated larvae (Fig. EV5K). Strikingly, pharmacological inhibition of glycogen phosphorylase in larvae injected with proliferative ZMEL cells induced an increase in their invasive capacity more than 2.5-fold and to levels comparable to those of untreated invasive cells (Fig. 9H,I), demonstrating that acute blockade of glycogen mobilization is sufficient to phenocopy the invasive state in vivo.

Taken together, the data provides strong in vivo evidence that glycogen phosphorylase activity restrains tumor cell invasiveness, and that its pharmacological inhibition promotes a pro-invasive phenotype across independent experimental systems.

## Discussion

Over recent years, it has become clear that microenvironment-driven transitions between phenotypic states underpins cancer progression and makes a major contribution to therapy resistance. Understanding how cells initiate and maintain the switch from proliferation to invasion is therefore a major issue. Here we reveal that melanoma MITF^High^ proliferative phenotype cells, but not MITF^Low^ invasive cells, store glucose as glycogen. This observation is consistent with the notion that invasive cells are starving (García-Jiménez and Goding, 2019), either because they are exposed to limiting nutrients or because they fail to take up, sense or use the nutrients available. Consequently, while proliferative cells can accumulate glycogen when nutrients are abundant, invasive cells are unable to do so. In line with this, proliferative melanoma cells exhibit increased levels of PGM1, an enzyme that catalyzes the interconversion of G1P and G6P at a key glucose trafficking point: feeding glucose into glycogen synthesis under extracellular glucose-replete conditions, or conducting glucose away from glycogen degradation towards glycolysis and biosynthetic pathways on glucose limitation (Jin et al, 2018). Consequently, the proliferative IGR37 melanoma cell line exhibited low levels of G6P, G1P and UDP-glucose, reflecting efficient glucose channeling into glycogen synthesis and elevated glycogen turnover consistent with a pro-survival role for PGM1 upon glucose deprivation (Curtis et al, 2019; Li et al, 2020; Cao et al, 2021).

Glycogen accumulation in melanoma appears to be promoted directly and indirectly by MITF, whose expression is suppressed by nutrient limitation (Falletta et al, 2017; Ferguson et al, 2017), with low MITF facilitating invasion (Carreira et al, 2006) as a pro-survival strategy to seek new nutrient supplies. Since the adoption of an invasive phenotype renders migrating cells sensitive to ferroptosis, anoikis or an unresolved integrated stress response (García-Jiménez and Goding, 2019; Zhang et al, 2022), a decision to switch to invasion is not to be taken lightly. We suggest that glycogen acts as a “metabolic buffer” by which glycogen mobilization stabilizes the proliferative state by preventing or delaying a switch to an invasive phenotype that would otherwise be triggered by transient glucose limitation. Although here we have focused on glycogen storage, glycogen synthesis in melanoma is embedded within a broader signaling network in which the PI3K–AKT-mTORC1, AMPK, and PKA pathways converge to regulate the balance between GYS1-mediated synthesis and PYG-mediated mobilization, with additional allosteric tuning by metabolites including glucose 6-phosphate, AMP, and ATP (Zois et al, 2014). While our study focuses on glycogen as the primary metabolic buffer within this network, we acknowledge that glycogen depletion provokes downstream adaptations in glycolysis, oxidative phosphorylation, and lipid metabolism that may also contribute to phenotype switching. It is also likely that there exist other intra- or extracellular cellular metabolic buffers, for example, lipid droplets (a source of carbon) or extracellular matrix (a source of nitrogen), that may act as nutrient reservoirs that shape the dynamics of phenotype switching.

Our observations using proliferative and invasive colorectal cancer cells suggest that the role of glycogen as a metabolic buffer stabilizing the proliferative state is likely to apply to cancer types other than melanoma. Indeed, Kaplan–Meier analysis of a variety of cancer types, including melanoma, in the Human Protein Atlas confirms that high PGM1 protein content strongly associates with improved patient survival. In support of this, glycogen metabolism has previously been implicated in cancer biology across multiple tumor types, however the available data remain scarce, heterogeneous, and derived from studies that do not distinguish metastatic status (Curtis et al, 2019; Liu et al, 2021; Altemus et al, 2019). For example, high glycogen content has been reported to inversely correlate with proliferation (Favaro et al, 2012; Curtis et al, 2019), and PYG inhibition or ablation to reduce proliferation, migration, or metastatic potential in several models (Schnier et al, 2003; Ma et al, 2012; Barot et al, 2019). Moreover, earlier studies described glycogen accumulation in melanoma tissue (Nowak et al, 1998) and glycogen loss during malignant transformation in intestinal epithelial cells (Gutiérrez-Salmerón et al, 2020). However, none of these reports examined whether glycogen metabolism directly regulates phenotype switching across defined tumor states or whether phenotype can dictate glycogen accumulation. Our results suggest that glycogen content decreases in advanced primary melanomas (Clark IV) and metastases compared to nevi or early melanoma stages (Clark I), consistent with the idea that glycogen suppresses invasiveness. Moreover, human primary tumors in patients with distant metastases exhibited higher levels of PGM1 and PYGB levels. These observations made on human melanomas were recapitulated in our mouse model, where matched lung metastases exhibited significantly lower PAS staining and reduced PYGB expression compared with their corresponding primary tumors. One limitation of the murine melanoma model is that when measuring metastatic dissemination, we could not discriminate between the effects of the PYGi on invasion, survival or outgrowth of metastases. Nevertheless, while a role for glycogen usage in survival or metastatic outgrowth remains likely, compelling evidence from the zebrafish larval transplantation model revealed that pharmacological blockade of glycogen phosphorylase in proliferative phenotype ZMEL1 cells was sufficient to phenocopy the invasive state in vivo at single-cell resolution.

Glycogen phosphorylases (PYG) catalyze glycogen degradation and are present in melanoma as two isoforms that differ in their allosteric regulation: PYGL activity is hormonally controlled by insulin and glucagon signaling, while PYGB activity depends on intracellular nutrient availability through the AMP:ATP ratio (Mathieu et al, 2017). This mode of regulation confers an advantage to PYGB in cancer cells, where its upregulation may sustain proliferation by driving glycogen catabolism until stores are depleted, ultimately contributing to invasion (Altemus et al, 2019; Tashima et al, 2000). These findings are consistent with increased PYGB levels reported in other cancers (Altemus et al, 2019; Jiang et al, 2022; Ren et al, 2024). By contrast, studies in other tumors have associated elevated PYGB with worse prognosis (Altemus et al, 2019; Matsuzaki et al, 1998; Zhou et al, 2019; Yang et al, 2024), suggesting a role context-dependent in cancer progression.

Finally, consistent with previous studies (Müller et al, 2014; Konieczkowski et al, 2014; Rambow et al, 2018), we found that selection for outgrowth of BRAFi-resistance cells led to elevated MITF expression, upregulation of PGM1 and PYGL levels, increased glycogen accumulation, enhanced metabolic flexibility, and reduced invasive capacity. These observations raise the question as to whether pharmacological inhibition of glycogen metabolism might be a useful therapeutic. In non-small cell lung carcinoma, pancreatic cancer and HCC (Ma et al, 2012; Barot et al, 2019; Schnier et al, 2003), the glycogen phosphorylase inhibitors CP-91149 and CP-320626 have shown potent antiproliferative effects. However, our results show that in melanoma, CP-91149 only decreased cell proliferation under glucose-limiting conditions in proliferative MITF^High^ cells, revealing that glycogen stores buffer against energetic stress rather than being essential under basal conditions. Nevertheless, while cancer cells may be under energetic stress in vivo, caution is needed. Even if treatment with glycogen phosphorylase inhibitors decreases proliferation, our results suggest they may increase invasion. However, in the murine B16 transplant model, mice treated with CP-91149 exhibited reduced primary tumor growth and no metastases. This may reflect a cell-intrinsic limitation in the invasive capacity of melanoma cells in the primary tumor, for example, by restricting their ability to survive their metastatic journey. Alternatively, systemic blockade of glycogen phosphorylase activity may limit glycogen bioavailability in metastatic target organs such as the liver, rendering these tissues less permissive to tumor colonization or metastatic outgrowth.

In summary, our work identifies a previously unrecognized, phenotype‑specific role for glycogen: rather than serving solely as a fuel reserve, glycogen acts as a temporal metabolic buffer that delays the proliferative-to-invasive transition under glucose stress. This principle is likely to extend to a range of cancers beyond melanoma. Our results, therefore, have major implications for our understanding of cancer progression and therapy resistance.

## Methods

**Table:** Reagents and tools table

Reagent/resource	Reference or source	Identifier or Catalog Number
**Experimental models**		
Cell line: IGR37	Luisa Lanfrancone Lab (Aubert et al, 1980)	RRID: CVCL_2075
Cell line: 501mel	Ruth Halaban Lab (Zakut et al, 1993)	RRID: CVCL_4633
Cell line: A375	ATCC (Fogh et al, 1977; Giard et al, 1973)	RRID: CVCL_0132
Cell line: IGR39	Luisa Lanfrancone Lab (Aubert et al, 1980)	RRID: CVCL_2076
Cell line: WM793	Meenhard Herlyn Lab (Herlyn et al, 1985)	RRID: CVCL_8787
Cell line: SKMEL28	ATCC (Fogh et al, 1977)	RRID: CVCL_0526
Cell line: WM983B	Meenhard Herlyn Lab (Herlyn et al, 1985)	RRID: CVCL_6809
Cell line: WM266.4	Meenhard Herlyn Lab (Herlyn et al, 1985)	RRID: CVCL_2765
Cell line: WM1366	Meenhard Herlyn Lab (Herlyn et al, 1985)	RRID: CVCL_6789
Cell line: WM278	Meenhard Herlyn Lab (Herlyn et al, 1985)	RRID: CVCL_6473
Cell line: B16-F10-Luciferase	Luis Sanchez del Campo. (Sánchez-del-Campo et al, 2021)	
Cell line: B16-F10-Luciferase MITF KO	Luis Sanchez del Campo. (Sánchez-del-Campo et al, 2021)	
Cell line: KM12C	I. Fidler’s Lab. (Morikawa et al, 1988a)	RRID: CVCL_9547
Cell line: KM12SM	I. Fidler’s Lab. (Morikawa et al, 1988a)	RRID: CVCL_9548
Cell line: ZMEL1-PRO (tdTomato)	R. White Lab (Campbell et al, 2021)	
Cell line: ZMEL1-INV (EGFP)	R. White Lab (Campbell et al, 2021)	
Nude Foxn1 nu/nu mice	Envigo	
Larval *casper* zebrafish	R. White Lab (White et al, 2008)	
**Antibodies**		
Mouse monoclonal anti-Cytokeratin 20	Dako Omnis, Agilent Technologies	Cat. #GA77761-2
Mouse monoclonal anti-GAPDH	Sigma-Aldrich	Cat. #G8795
Rabbit monoclonal anti-GYS (1/2)	Cell Signalling	Cat. #3886
Rabbit monoclonal anti-Lamin B1	Invitrogen	Cat. #702972
Mouse monoclonal anti-MITF	Abcam	Cat. #Ab12039
Rabbit monoclonal anti-PGM1	Abcam	Cat. #Ab192876
Rabbit polyclonal anti-PYGB	Sigma-Aldrich	Cat. # HPA031067
Rabbit polyclonal anti-PYGB	Cell Signaling	Cat. # 50340
Rabbit polyclonal anti-PYGL	Sigma-Aldrich	Cat. #HPA000962
Rabbit polyclonal anti-PYGL	Cell Signaling	Cat. # 42103
Rabbit monoclonal anti-TBP	Cell Signalling	Cat. #44059
Mouse monoclonal anti-α-Tubulin	Sigma-Aldrich	Cat. #T5168
Goat Anti-Mouse IgG (H + L) HRPO	Bio-Rad	Cat. #170-6516
Goat Anti-Rabbit IgG (H + L) HRPO	Bio-Rad	Cat. #170-6515
**Oligonucleotides and other sequence-based reagents**		
siMITF 5′-AAAGCAGTACCTTTCTACCAC-3′	Sigma-Aldrich	Falletta et al, 2017
ON-TARGETplus Non-targeting Pool (negative control siRNA)	Dharmacon Inc	Cat. #D-001810-10-05
SMARTpool: ON-TARGETplus human PGM1 (5236) siRNA	Dharmacon Inc	Cat. #L-010925-01-0005
PGM1 lentiviral vector (human)	Abm Inc.	Cat. #LC136510
Lenti-III-Blank Lentivirus	Abm Inc.	Cat. #LVP687
**Chemicals, Enzymes and other reagents**		
7-AAD	Santa Cruz Biotechnology	Cat. #sc-221210
2-Deoxy-D-Glucose	Sigma-Aldrich	Cat. #D8375
2-NBDG (2-(N-(7-nitrobenz-2-oxa-1,3-diazol-4-il)amino)-2-desoxiglucosa)	Thermo Fisher Scientific	Cat. #N13195
2-(N-Morpholino)ethanesulfonic acid (MES)	Dojindo	Cat.# 349–01623
3-Aminopyrrolidine	Sigma-Aldrich	Cat. #404624
Antimycin A	Sigma-Aldrich	Cat. #A8674
BioCoat™ Matrigel™ Invasion Chamber (8 µm)	Corning	Cat. #45354480
Borosilicate glass capillary needle	Sutter Instrument	Cat. #Q100-50-10
Bradford	Sigma-Aldrich	Cat. #B6916
Clarity ECL detection kit	Bio-Rad	Cat. #170-5061
Collagen type I	Corning	Cat. #354249
CP-91149	Selleckhem	Cat. #S2717
CP-91149	MedChemExpress	Cat. #186392-40-5
Crystal violet	Sigma-Aldrich	Cat. #C6158
D-Camphor-10-sulfonic acid	Wako	Cat. #037–01032
D – ( + ) – Glucose	Sigma-Aldrich	Cat. #G8769
DMEM	Corning	Cat. #702512
DMEM GlutaMAX-I	Gibco	Cat. #31966
DMSO	Sigma-Aldrich	Cat. #D2650
DPBS	Gibco	Cat. #14190-144
Diastase	Sigma-Aldrich	Cat. #09962
EnVision + Dual link System HRP (DAB + )	EnVision, Dako, Agilent Technologies	Cat. #K4065
EnVision + System-HRP Labeled Polymer Anti-rabbit	EnVision, Dako, Agilent Technologies	Cat. #K4002
ERCC ExFold RNA Spike-In Mixes	Thermo Fisher	Cat. #4456739
Fetal Bovine Serum	Sigma-Aldrich	Cat. #F7524
Formalin	Sigma-Aldrich	Cat. #HT501128
Glass capillary needle	Sutter	Cat. #Q100-50-10
Glucose (1 M)	Agilent	Cat. #103577-100
Glutamine (200 mM)	Agilent	Cat. #103579-100
Glucose Assay kit	Abcam	Cat. #ab65333
Glucose 1 phosphate (G1P) Colorimetric Assay Kit	Sigma-Aldrich	Cat. #MAK098
Glucose 6-phosphate (G6P) Colorimetric Assay Kit	Sigma-Aldrich	Cat. #MAK014
Glycolytic Rate Kit	Agilent Technologies	Cat. #103344-100
Glycolytic Stress Test Kit	Agilent Technologies	Cat. #103020-100
Haematoxylin	Sigma-Aldrich	Cat. #GHS316
Harris Haematoxylin	Sigma-Aldrich	Cat. #HHS32
HRP Magenta Chromogen	Dako Agilent Technologies	Cat. #GV900
JetPRIME PolyPlus reagent	Polyplus transfection	Cat. #114-15
L-Glutamine (200 mM)	LONZA	Cat. #BE17-605E
L-Methionine sulfone	Wako	Cat. #502–76641
Matrigel® Growth Factor Reduced (GFR) Basement Membrane Matrix	Corning	Cat. #356231
MEM NEAA	Thermo Fisher Scientific	Cat. #11140035
Mito Fuel Flex Test Kit	Agilent	Cat. #103260-100
MTT (Thiazolyl Blue Tetrazolium Bromide)	Sigma-Aldrich	Cat. #M5655
Nuclease-free water	Invitrogen	Cat. #10977049
Nunclon™ Sphera™ 3D ULA plates	Thermo Fisher Scientific	Cat. #174925
Oligomycin A	Sigma-Aldrich	Cat. #75351
PBS	Corning	Cat. #BE02-017F
PE Annexin V Apoptosis Detection Kit I	BD Biosciences	Cat. #559763
Penicillin-Streptomycin	LONZA	Cat. #DE17-602E
Periodic acid	Sigma-Aldrich	Cat. #3951
Peroxidase blocking reagent	Dako Agilent Technologies	Cat. #S202386
PLX4032 (Vemurafenib)	Selleckchem	Cat. #S1267
Propidium iodide	Sigma-Aldrich	Cat. #81845
Protease inhibitor cocktail	Roche	Cat. #04693132001
QuantSeq Forward kit	LEXOGEN	Cat. #015.96
Real-Time ATP Rate Kit	Agilent Technologies	Cat. #103592-100
RNA 6000 Nano Kit	Agilent Technologies	Cat. #5067-1511
RNase A	Thermo Fisher Scientific	Cat. #12091-021
rNeasy Mini Kit	QIAGEN	Cat. #74106
Rotenone	Sigma-Aldrich	Cat. #R8875
RPMI 1640	Corning	Cat. #5510-041-cv
RPMI 1640 (without glucose)	Corning	Cat. #5510-043-cv
RPMI pH 7.4, 1 mM HEPES media	Agilent	Cat. #103576-100
Shift Reagent	Sigma-Aldrich	Cat. #3952016
Sodium pyruvate (100 mM)	Agilent	Cat. #103578-100
Tricaine-S	Syndel USA	Cat. # MS-222
Trimesate	Wako	Cat. #206–03641
TrypLE Express Enzyme	Gibco	Cat. #12604013
Tween 20	Sigma-Aldrich	Cat. #P1379
**Software**		
Agilent Seahorse Analytics	Agilent	Version 2.6.1
Bowtie	Johns Hopkins University	Version 2.5.4
CytExpert	Beckman Coulter	Version 2.6
Excel	Windows	Version 2509
FastQC	Babraham Bioinformatics	Version 0.11.9
Fiji	National Institutes of Health (USA)	Version 1.53t
Gene Cluster	Eisen Lab (USA)	Version 3
GraphPad Prism	GraphPad Software	Version 8.0.1
IBM SPSS Statistics	IBM Corporation	Version 31
ImageLab	Bio-Rad	Version 6
Leica LAS AF	Leica Microsystems	Version 3.6.0.20104
Motic Images Plus ML	Motic China Group Co., Ltd.	Version 2
Power Point	Windows	Version 2509
R package pheatmap	Raivo Kolde, CRAN	Version 1.0.12
STAR	Cold Spring Harbor Laboratory	Version 2.5.1b
TCGA analysis R-script	N/A	
TreeView	Eisen Lab (USA)	Version 8
**Other**		
Beckman Coulter CytoFLEX Flow Cytometer	Beckman Coulter	
CE-MS adapter kit G1603A	Agilent Technologies	
CE-ESI-MS sprayer kit G1607A	Agilent Technologies	
ChemiDoc^TM^ XRS+ System	Bio-Rad	
AxioZoom V16 Fluorescence stereomicroscope	Zeiss	
HiSeq 4000 System	Illumina	
HPLC 1100 Series isocratic pump	Agilent Technologies	
Mass spectrometer G6220A LC/MSD TOF	Agilent Technologies	
Molecular Signatures Database (MSigDB)	Broad Institute (USA)	
Moticam 10 + 10.0	Motic	
MTA-I Manual Tissue Microarrayer	Beecher Instruments	
Laser-Based Micropipette Puller P-2000	Sutter	
Picoliter Microinjector PLI-100A	Warner Instruments	
Seahorse XFe24 Analyzer	Agilent	
SpectraFLUOstar Omega Spectrofluorimeter	BMG Labtech	
Trans-Blot Turbo Transfer System	Bio-Rad	

### Cell lines

Human cell lines (IGR37, IGR39, 501mel, A375, A375R, WM266.4, SKMEL28, WM1366, WM278, WM793, and IGR39) were cultured in RPMI, murine (B16), CRC cells (KM12C and KM12SM), and zebrafish (ZMEL1) cell lines in DMEM with 10% FBS with 1% penicillin-streptomycin, and maintained in 5% CO_2_ environment at 37 °C. All cell lines were authenticated by STR profiling, and mycoplasma contamination tests by PCR were run monthly to ensure no contamination. To generate Vemurafenib‑resistant melanoma cells, A375 cells were seeded in 25 cm² culture flasks. When cultures reached ~80% confluence, cells were exposed to increasing concentrations of Vemurafenib, starting at 0.1 μM and gradually escalating to 1 μM over a period of 4 months. The selection process continued for a total of 6 months until cells exhibited stable growth in medium containing PLX4032.

### RNAi gene silencing

Cells plated in six-well plates at 50% confluence were transfected with siRNA using JetPRIME reagent (Polyplus transfection), following the manufacturer's instructions. After 2 days, cells were treated and collected to perform an invasion assay or to be analyzed by Western blot. Specific siRNA oligonucleotides for PGM1 and control siRNA were obtained from Dharmacon Inc, and for MITF from Sigma-Aldrich.

### Cell transduction

Target cells were seeded at 3 × 10⁵ cells per well in a six-well plate 24 h prior to transduction. Lentiviral particles carrying either PGM1 or an empty vector were added at a multiplicity of infection (MOI) of 10 in the presence of polybrene (8 µg/mL) to enhance transduction efficiency. Medium was replaced 24 h post-transduction with fresh complete medium. After 2 days, cells were treated to perform an invasion assay or to be analyzed by Western blot.

### Preparation of cell extracts

Cells were washed with iced PBS before extract preparation and scraped in RIPA buffer (10 mM Tris-HCl, pH 7.4, 5 mM EDTA, 5 mM EGTA, 1% Triton X-100, 10 mM Na_4_P_2_O_7_, pH 7.4, 10 mM NaF, 130 mM NaCl, 0.1% SDS, 0,5% Na-deoxycholate). After 5 min on ice, cells were pelleted (12,000 rpm for 5 min, 4 °C) and the supernatant was directly used as whole cell extract or frozen at −80 °C.

### Western blot

Protein lysates were subjected to 10 to 15% polyacrylamide SDS-PAGE. Proteins were transferred onto polyvinylidene difluoride membranes (PVDF). Membranes were blocked with 5% non-fat milk in T-TBS containing 0.1% Tween 20 and probed with the appropriate primary antibodies (see Key Resources Table) overnight at 4 °C. The specific bands were analyzed using ChemiDoc Imaging Systems (Bio-Rad).

### Crystal violet staining and cell density quantification

Cells were plated at a density of 2 × 10⁴ cells per well in a 12-well plate and cultured for 1 day before glucose depletion and/or treated with CP-91149 or 2DG over 5 days as indicated. Both parental and vemurafenib-resistant A375 cells were seeded at the same density. The medium was replaced every 3 days. Plates were collected at time 0, 12, 24, 48, 72, or 120 h, fixed with 4% paraformaldehyde, stained with 0.5% crystal violet for 15–30 min, washed with tap water and dried. Crystal violet was resuspended in methanol, transferred to 96-well plates and analyzed by a SpectraFLUOstar Omega plate reader (BMG Labtech). Viability was measured in duplicate.

### Matrigel invasion assays

Matrigel invasion assays were performed using BD BioCoat invasion chambers with 8 µm Matrigel-coated membrane inserts, following the manufacturer’s instructions. Melanoma cells treated or transfected as indicated were cultured in medium without 0.2% FBS overnight, and 100,000 cells were seeded per insert. Medium with 10% FBS was added to the bottom of the inserts as a chemoattractant. After 22 h, chambers were fixed in 4% paraformaldehyde for 2 min, washed in PBS, permeabilized 20 min with 100% methanol, washed again, stained with 1% crystal violet for 20 min and washed in PBS. Cells remaining above the insert were removed by gentle scraping with a sterile cotton swab. Slides were mounted, and images were acquired using a Zeiss optical microscope with a 5x objective. Cells were manually counted using ImageJ software. At least three biological replicates were performed.

### G1P and G6P measurement

Glucose 1 phosphate (G1P) and glucose 6-phosphate (G6P) cell concentrations were determined by colorimetric assays following the manufacturer's instructions (Sigma-Aldrich). Cells growing in 11 mm glucose were depleted for glucose (1 mM) for 24 h. 1 × 10^6^ cells were homogenized in 2–3 volumes of ice-cold PBS and centrifuged the samples at 13,000×*g* for 10 min to remove insoluble material. About 25 µl of sample were diluted with 25 µl of assay buffer and incubated with 50 µl of the appropriate Reaction Mix, incubate the reaction for 30 min at room temperature and measured the absorbance at 450 nm. G1P and G6P standards were used to plot a standard curve to determine the concentration (ng/µL) of each metabolite in each sample. Measurements were determined in duplicates in at least three different experiments.

### PAS and PAS-D staining

Melanoma cells seeded in coverslips were washed three times in PBS, fixed with 4% paraformaldehyde in PBS (pH 7.4) for 10 min, and washed again. For PAS-D staining, slides were incubated with 0.2% α-amylase (Diastase, Sigma-Aldrich) in PBS for 30 min at 37 °C and rinsed in tap water and then with distilled water. For PAS staining, this step was performed with PBS. Slides were then oxidized for 5 min with 1% solution of periodic acid (Sigma-Aldrich), washed with tap water for 3 min, washed with distilled water for 1 min, and incubated in Schiff’s reagent (Sigma-Aldrich) for 20 min. Slides were washed first with distilled water for 5 s and then with tap water for 10 min. Counterstaining was performed with Hematoxylin Solution (Sigma-Aldrich) for 2 min and washed three times for 5 min with PBS.

For tissue sections, slides were deparaffinized and hydrated before diastase digestion (1% α-amylase 30 min at 37 °C) and PAS staining: 10 min in 1% solution of periodic acid followed by washes and incubation in Schiff’s reagent for 20 min. Slides were counterstained with Hematoxylin for 30 s. Images were acquired using a microscope (Zeiss) with a 40× objective for cultured cells or 20× for tissue sections*.*

### Glucose (2-NBDG) uptake assay

Cells were seeded in six‑well plates and cultured for 24 h in complete medium. Culture medium was removed and replaced with RPMI (glucose‑ and sodium pyruvate‑free) supplemented with 10% FBS, and plates were incubated at 37 °C for 1 h. The fluorescent D‑glucose analog 2‑[*N*‑(7‑nitrobenz‑2‑oxa‑1,3‑diazol‑4‑yl)amino]‑2‑deoxy‑D‑glucose (2‑NBDG), was added to the medium at a final concentration of 5 μM. Cells were incubated with 2‑NBDG for 1 h at 37 °C. The uptake reaction was terminated by removing the incubation medium and washing cells twice with ice‑cold 1× PBS. Cells were harvested, centrifuged at 2000 rpm for 5 min at 4 °C, and resuspended in 500 μL ice‑cold PBS. Propidium iodide (PI) was added to a final concentration of 1 μg/mL to discriminate nonviable cells. Flow cytometry data were acquired on a Beckman Coulter CytoFLEX cytometer, collecting 50,000 single‑cell events per sample. Data was analyzed using CytExpert software. 2‑NBDG uptake was quantified as the mean fluorescence intensity (MFI) of viable cells and expressed relative to control samples.

### Glucose measurement in culture medium

Cells were seeded in six‑well plates, transfected or treated as indicated. After 3 days, glucose concentration in culture medium was measured using the Glucose Assay Kit (Abcam) according to the manufacturer’s instructions. Culture supernatants were diluted in assay buffer to fall within the standard curve range, and 50 µl aliquots were loaded in a 96‑well plate; 50 µl of reaction mix was added to standards and samples (50 µl background reaction mix to background wells), plates were incubated at 37 °C for 30 min protected from light, and absorbance (570 nm) was recorded. Sample signals were corrected by subtracting background, nmol glucose per well was interpolated from the standard curve, and the sample concentration was calculated. For each experiment, concentrations were normalized to the mean value of the designated control group to obtain relative extracellular glucose.

### Apoptosis assay by flow cytometry

For the assessment of early and late apoptosis, a commercial cell apoptosis assay kit (BD Biosciences) was used according to the manufacturer’s instructions. After 3 days of treatment, cells were harvested, washed with ice‑cold 1× PBS and resuspended in 1× binding buffer at 1 × 10^6^ cells/ml. 100 μl aliquots (1 × 10^6^ cells) were transferred to flow cytometry tubes and stained with 5 μl PE‑Annexin V and 5 μl of 7‑AAD (BD), mixed gently and incubated for 15 min at room temperature protected from light. About 400 μl of 1× Binding Buffer was added prior to acquisition, and samples were analyzed immediately by flow cytometry on a Beckman Coulter CytoFLEX cytometer, collecting 50,000 single‑cell events and gating to exclude debris and doublets. Data were analyzed using CytExpert software. Viable (Annexin V−/7‑AAD−), early apoptotic (Annexin V+/7‑AAD−) and late apoptotic/necrotic (Annexin V+/7‑AAD+) percentage of cells were analyzed. Unstained, single‑stain and positive apoptosis (cells treated with vemurafenib) controls were included in each experiment.

### Cell adhesion assay

A 40 μg/ml solution of collagen I (Corning) in PBS was used to coat 96-well plates, incubating at 4 °C for 12 h, followed by air-drying at room temperature. Prior to the assay, cells were starved for 8 h in serum-free RPMI with corresponding treatments. Cells were detached and resuspended at a density of 2 × 10⁵ cells/mL in treated medium containing 0.1% FBS. A 100 μl of the cell suspension was added to each collagen-coated well and incubated at 37 °C for 20 min. Non-adherent cells were removed by washing four times with RPMI. After recovery in RPMI with 10% FBS for 4 h, cell viability was assessed using the MTT assay. For that, the cells were incubated with MTT (1:10 dilution in culture medium; final concentration ~0.5 mg/mL) at 37 °C for 3 h. Following incubation, the supernatant was removed, images were captured using a Moticam camera (Motic), attached to an inverted optical microscope (Zeiss). The number of cells per well and condition was counted using ImageJ software. Values were normalized to the control group. All treatments were assayed in duplicate, and each experiment was repeated independently three times.

### Colony formation assay

Cells were harvested and seeded at a density of 2.5 × 10^3^ cells per well onto a six-well plate, incubated at 37 °C for 12 h to allow attachment. Non-adherent cells were removed by washing the wells with RPMI, and adherent cells were then treated with RPMI containing 11 or 1 mM glucose, with or without CP, as appropriate. Cells were incubated at 37 °C for 1 week, until colonies in control wells reached a sufficient size, with a minimum of 50 cells per colony for scoring. Cells were then fixed in 4% paraformaldehyde, stained with a 0.5% crystal violet solution for 2 h at room temperature and air-dried overnight. To quantify the number of colonies per condition, images were taken with a Moticam camera (Motic).

### Cell cycle

Cells were seeded at 50% confluence in six‑well plates, treated for 2 days, harvested by trypsinization, and washed with PBS. Cells were then fixed in 70% ethanol for 1 h on ice, stained with propidium iodide (PI; 16 µg/mL) and RNase A (1.2 mg/mL) for 30 min at 37 °C in the dark, and analyzed by flow cytometry (Beckman Coulter CytoFLEX). Cell‑cycle distribution was quantified using CytExpert software. A minimum of three independent biological replicates was performed for each condition, and results are expressed as the percentage of cells in each cell‑cycle phase.

### Tumor spheroid-based Matrigel invasion assay

For spheroid generation, 200 μl per well of WM266.4 cell suspensions at 0.1 × 10⁴cells/ml were dispensed into ULA 96‑well round‑bottom plates (Nunclon). Plates were incubated for 4 days at 37 °C in a humidified atmosphere containing 5% CO₂. To assess invasive capacity, 100 μl of medium was removed from each well containing 4‑day spheroids, 100 μL of Matrigel (Corning) was gently added, incubated at 37 °C with 5% CO₂ for 1 h to allow Matrigel polymerization, and then 100 μL of culture medium was added on top. For compound‑evaluation studies, spheroids were pre‑treated for 8 h and exposed to the compounds throughout the assay as indicated. Images were acquired at t₀ and every 24 h up to 72 h using a Moticam camera. Invasiveness was quantified by segmenting the spheroid‑covered area into two subregions using Fiji software: core and edge. The core corresponds to the dense central region where cells remain tightly aggregated, whereas the edge represents the peripheral area where cells invade the surrounding matrix as dispersed projections. These regions were extracted as mask images and the invaded area was quantified.

### Metabolomic analysis

For metabolite extraction, cells grown in 10 cm dishes were rinsed twice with 5% mannitol in MilliQ water (10 ml per wash) to remove extracellular metabolites. Cell pellets were then resuspended in 1 ml methanol and incubated for 10 min to extract intracellular metabolites. Protein precipitation was achieved by adding 400 µl chloroform (CHCl₃) and 200 µl MilliQ water, followed by centrifugation at 10,000 × *g* for 3 min at 4 °C. A volume of 400 µl of the resulting aqueous phase was passed through a 5 kDa molecular weight cut-off ultrafiltration tube to remove residual proteins and macromolecules.

Prior to analysis, 25 µl of a 200 mM internal standard mixture was added to each sample, comprising L-methionine sulfone (Wako), 2-(*N*-morpholino)ethanesulfonic acid (Dojindo), D-camphor-10-sulfonic acid (Wako), 3-aminopyrrolidine (Sigma-Aldrich), and trimesate (Wako). Metabolite profiling was carried out by capillary electrophoresis–mass spectrometry (CE-MS) following the method described by Kami et al. (Kami et al, 2013), using an Agilent CE system coupled to an Agilent G6220A LC/MSD TOF mass spectrometer, an Agilent 1100 series isocratic HPLC pump, a G1603A CE-MS adapter kit, and a G1607A CE-ESI-MS sprayer kit (Agilent Technologies).

### Seahorse experiments

Cellular metabolism was analyzed by measurement of glycolytic rate for basal conditions and compensatory glycolysis following mitochondrial inhibition (Agilent Seahorse XF Glycolytic Rate Assay), ATP production from mitochondrial oxidative phosphorylation (OXPHOS) and glycolysis (Agilent Seahorse XF Real-Time ATP Rate Assay), key parameters of glycolytic function (Agilent Seahorse XF Glycolysis Stress Test) and the mitochondrial metabolic flexibility (Agilent Seahorse XF Mito Fuel Flex Test Kit). Depending on the cell line and assay type, 15,000 to 25,000 cells were seeded in 100 µL of RPMI per well of 24-well plates (XF24) and after 4–6 h of incubation at 37 °C, once the cells were adhered to the plate and in monolayer, an additional 150 µL of RPMI medium was added. After 24–48 h, cell plates were washed twice with prewarmed Seahorse medium, following the manufacturer’s instructions for each assay type (Agilent Technologies), and incubated at 37 °C in the oven for 1 h. In parallel, the calibration cartridges were loaded into the equipment Seahorse XFe24 Analyzer (Agilent Technologies) with the appropriate treatments and after 20 min cell plates were loaded for 1–2 h. All experiments were performed at least in triplicate for each experimental condition, and the results were analyzed on the manufacturer’s own website (Seahorseanalytics.agilent).

### TMA, immunohistochemistry, and quantification

Human samples were obtained from Hospital Clínico San Carlos (HCSC). IRB-HCSC act number 21/498-E granting approval on June 25, 2021. All patients gave written informed consent for the use of their biological samples for research purposes. Fundamental ethical principles and rights promoted by Spain (LOPD 15/1999) and the European Union (2000/C364/01) were followed. In addition, all patients’ data were processed according to the Declaration of Helsinki (last revision 2013) and the Spanish National Biomedical Research Law (14/2007, of July 3).

Tissue microarrays (TMA) containing 69 cores of nevus (*n* = 14), melanoma (*n* = 27) or metastasis (*n* = 28) samples, were constructed using the MTA-1 tissue arrayer (Beecher Instruments, Sun Prairie) for immunohistochemistry analysis. Each core (diameter 0.6 mm) was punched from pre-selected tumor regions in paraffin-embedded tissues. Central tumor areas were selected, avoiding necrotic foci. Staining was conducted in 2-μm sections. Slides were deparaffinized by incubation at 60 °C for 10 min and incubated with PT-Link (Dako, Agilent) for 20 min at 95 °C in low pH to detect PGM1, PYGB and PYGL or in high pH to detect GYS. To block endogenous peroxidase, holders were incubated with peroxidase blocking reagent (Dako, Agilent) and then with (1:50) dilutions of antibody anti-PGM1, (1:25) anti-GYS, (1:100) anti-PYGB, and (1:150) anti-PYGL, overnight at 4 °C. All previously described antibodies presented high specificity. After that, slides were incubated for 20 min with the appropriate anti-Ig horseradish peroxidase-conjugated polymer (EnVision, Dako, Agilent). Sections were then visualized with HRP Magenta (Dako, Agilent) as a chromogen for 5 min and counterstained with Harris’ Hematoxylin (Sigma-Aldrich). Photographs were taken with a microscope (Zeiss) with a 20x objective. Immunoreactivity was quantified blind with a Histoscore (H-score) that considers both the intensity and percentage of cells stained for each intensity (low, medium, or high) following this algorithm (range 0–300): H-score = (low%) × 1 + (medium%) × 2 + (high %) × 3. Quantification for each patient biopsy was calculated blindly by 2 investigators (MJFA and JMU). Clinicopathological characteristics of patients are summarized in Table 1.

### Mice experiments

All experiments were performed in accordance with a protocol that was approved by the Ethics Committee of the University of Murcia and the Dirección General de Ganadería y Pesca, Comunidad Autonoma Region de Murcia (project reference A13230906). Female nude Foxn1 nu/nu mice, aged 6–8 weeks, were obtained from Envigo (Barcelona, Spain) and housed in a pathogen-free facility at the University of Murcia. Tumors were established by subcutaneously injecting 2 × 10⁵ B16-F10 luciferase cells into the flank region on one side. After 1 week, the mice were separated into two groups of similar weight. Mice were then administered the vehicle or the glycogen phosphorylase inhibitor (PYGi) CP-91149 (MedChemExpress, 50 mg/kg) intraperitoneally every day for eight days, as indicated in (Davidson et al, 2022). Tumor dimensions were measured three times weekly using a digital caliper, and tumor volume was calculated using the formula: volume (mm³) = length ×  width²/2. Mice were monitored regularly and sacrificed before any signs of distress or ulcer formation appeared. An IVIS imaging system (Caliper Life Sciences, Hopkinton, MA, USA) was used to monitor tumor growth at specified time points. Mice were euthanized by cervical dislocation, and the tumors were harvested, fixed in formalin and embedded in paraffin for subsequent immunohistochemical staining.

### Zebrafish husbandry

Zebrafish were housed in the Institute for Development and Regeneration aquatics facility at the University of Oxford. Fish were maintained at 28.5 °C with a light/dark cycle (14 h on, 10 h off). Larval *casper* zebrafish were used in this study (White et al, 2008). Anesthesia was performed using a 4 g/L, pH 7.0 stock Tricaine (Sigma-Aldrich). All zebrafish experiments were performed under Project License number PP2565788 granted by the Home Office.

### Larval transplantation and drug treatment

Transplantation of ZMEL1 cells into 2dpf Casper zebrafish larvae was performed as previously described (Haldi et al, 2006). Briefly, ZMEL1-PRO cells were detached using TrypLE Express Enzyme (Gibco), centrifuged at 300×*g* for 3 min, and resuspended at a concentration of 2.5 × 10^7^ cells/mL in 9:1 DPBS:H_2_O (Gibco) containing either 0.01% dimethyl sulfoxide (Sigma-Aldrich) or 1 µM CP-91149 (MedChemExpress). Cells were injected into the yolk sac of days post-fertilization (dpf) *casper* larvae using a Picoliter Microinjector (Warner Instruments) with a glass capillary needle (Sutter) made on a laser-based needle puller (Sutter). Larvae transplanted with DMSO-treated cells or CP-91149-treated cells were maintained in E3 embryo media containing either 0.01% DMSO or 1 µM CP-91149, respectively. Larvae with successful transplants based on the presence of fluorescent cells in the yolk sac were imaged on a Zeiss AxioZoom V16 fluorescence microscope and analyzed for extravasation events at 3 days post-transplant. Extravasation was quantified by calculating the number of fish with fluorescent cells outside the transplantation site (the yolk sac), including cells that migrated to peripheral and/or distal tissues. This analysis was done blinded with respect to drug vs. control treatment groups. Statistical analysis for this assay was performed using GraphPad Prism.

### RNA-seq

STR authenticated, mycoplasma-free melanoma cell lines were seeded in six-well plates in RPMI 1640 supplemented with 10% FBS and 100 U/ml Pen Strep and maintained in 10% CO_2_ humidified chamber. Cells were collected at around 80% confluence and snap-freeze on dry ice before batched processed using rNeasy Mini Kit (QIAGEN) as per supplier instructions and eluted in 50 µl nuclease-free water (Invitrogen). Samples with RIN values ≥9.5 (assessed using Agilent RNA 6000 Nano Kit) were carried forward for library prep using QuantSeq Forward kit (LEXOGEN) with 500 ng input material and ERCC ExFold RNA Spike-In Mixes (ThermoFisher). Sequencing was carried out on a HiSeq4000 (Illumina) by the Oxford Genomics Centre, Wellcome Trust Centre for Human Genetics.

### ChIP-seq analysis

ChIP experiments were performed as described in (Louphrasitthiphol et al, 2020). Each replicate comprised two technical replicates (the same library sequenced on two flow cells), which were merged into a single FASTQ file. Reads were quality-checked with FastQC, processed, and aligned to the human hg19 (GRCh37) reference using Bowtie, allowing up to two mismatches. Resulting SAM files were used for peak calling with HOMER, using background controls from a parallel HA ChIP-seq on 501mel parental cells. HOMER was also used for peak annotation, gene assignment (nearest gene), genome ontology analysis, de novo motif discovery, and bedgraph generation. To increase stringency, peaks with score <10 were discarded, and co‑occupancy was defined when peak summits or start coordinates lay within 200 bp. Data presented in this manuscript correspond to datasets previously reported in (Louphrasitthiphol et al, 2020).

### Bioinformatics

Raw FASTQ reads for the same samples sequenced across two lanes were stitched using UNIX, qCed using fastqc (v0.11.9), adaptor- and poly-A- trimmed using Cutadapt. Processed fastq were mapped against hg38 (GRCh38, 2015) + ERCC STAR index using rna-star (v2.5.1b) with quantMode enabled, allowing for soft-clipping and splicing (min 20 bp). Normalization and differential gene expression analyses were performed as previously described (Louphrasitthiphol et al, 2020, 2019) using edgeR glmQLFTest. Heatmaps were visualized using the R package pheatmap (v1.0.12).

Gene expression from 53 melanoma cell lines (Tsoi et al, 2018) were obtained from GSE80829 and their classification as per the original publication. Gene expression data from the CCLE (https://portals.broadinstitute.org/ccle) and TCGA (https://www.cancer.gov/tcga) were accessed through cBioportal (Gao et al; Cerami et al, 2012). Moving average trendline of bar plot representing the expression level of individual samples was generated using a simple window size of 20. Spearman correlation and the significance was calculated using cor.test function in R.

Gene expressions from samples of melanoma and healthy tissue adjacent to the tumor were analyzed with TNMplot.com (https://tnmplot.com/analysis/) tool 59. Kaplan–Meier curves were performed using the best cut-off point and data from 98 melanoma patients, 254 colorectal cancer patients, 80 pancreatic cancer patients, 521 kidney clear cell carcinoma patients, and 362 hepatocellular carcinoma patients from the TCGA and the curves were obtained from the Human Protein Atlas (https://www.proteinatlas.org/).

### Statistical analysis

Results are presented as fold induction, mean ± SEM from at least three biological replicates. At least three independent biological replicates were performed for all experiments with cell lines, with each condition tested in duplicate or triplicate within each experiment. Tests for significance between two sample groups were performed with Student’s *t*-test, and for multiple comparisons, ANOVA with Bonferroni’s post-test. All statistical analyses were performed with GraphPad Prism software. *P* values <0.05 were considered statistically significant, with a significance level of: *p < 0.05; **p < 0.01; or ***p < 0.001.

For immunohistochemical data, the Kolmogorov-Smirnov test was used to determine whether calculated H-scores for each of the antigens were well-modeled by a normal distribution. None showed a normal distribution. Analyses between groups were performed with the Mann–Whitney *U*-test and correlations with Spearman test (S) and associations with Chi-Square or Fisher’s exact test. All statistical analyses were performed with SPSS IBM software v.24. *P* values <0.05 were considered statistically significant.

## Supplementary information

Peer Review File

Source data Fig. 1

Source data Fig. 2

Source data Fig. 3

Source data Fig. 4

Source data Fig. 5

Source data Fig. 6

Source data Fig. 7

Source data Fig. 8

Source data Fig. 9

Expanded View Figures

## Data Availability

The authors declare that all data supporting the findings of this study are available within the article or from the corresponding author upon request. RNAseq datasets of all human melanoma cell are available in Gene Expression Omnibus (GEO) (https://www.ncbi.nlm.nih.gov/geo/query/acc.cgi?acc=GSE137392). ChIP-data for MITF in 501mel cells deposited in GEO with accession number (https://www.ncbi.nlm.nih.gov/geo/query/acc.cgi?acc=GSE137522). The source data of this paper are collected in the following database record: biostudies:S-SCDT-10_1038-S44318-026-00857-2.

## References

[CR1] Altemus MA, Goo LE, Little AC, Yates JA, Cheriyan HG, Wu ZF, Merajver SD (2019) Breast cancers utilize hypoxic glycogen stores via PYGB, the brain isoform of glycogen phosphorylase, to promote metastatic phenotypes. PLoS ONE 14:e0220973 31536495 10.1371/journal.pone.0220973 PMC6752868

[CR2] Aubert C, Bougé F, Galindo JR (1980) Tumorigenicity of human malignant melanocytes in nude mice in relation to their differentiation in vitro. J Natl Cancer Inst 64:1029–1040 6929009

[CR3] Barot S, Abo-Ali EM, Zhou DL, Palaguachi C, Dukhande VV (2019) Inhibition of glycogen catabolism induces intrinsic apoptosis and augments multikinase inhibitors in hepatocellular carcinoma cells. Exp Cell Res 381:288–300 31128107 10.1016/j.yexcr.2019.05.017

[CR4] Campbell NR, Rao A, Hunter MV, Sznurkowska MK, Briker L, Zhang M, Baron M, Heilmann S, Deforet M, Kenny C et al (2021) Cooperation between melanoma cell states promotes metastasis through heterotypic cluster formation. Dev Cell 56:2808–2825.e10 34529939 10.1016/j.devcel.2021.08.018 PMC8551056

[CR5] Cao B, Deng H, Cui H, Zhao R, Li H, Wei B, Chen L (2021) Knockdown of PGM1 enhances anticancer effects of orlistat in gastric cancer under glucose deprivation. Cancer Cell Int 21:481 34507580 10.1186/s12935-021-02193-3 PMC8434706

[CR6] Carreira S, Goodall J, Denat L, Rodriguez M, Nuciforo P, Hoek KS, Testori A, Larue L, Goding CR (2006) Mitf regulation of Dia1 controls melanoma proliferation and invasiveness. Genes Dev 20:3426–3439 17182868 10.1101/gad.406406 PMC1698449

[CR7] Cerami E, Gao J, Dogrusoz U, Gross BE, Sumer SO, Aksoy BA, Jacobsen A, Byrne CJ, Heuer ML, Larsson E et al (2012) The cBio cancer genomics portal: an open platform for exploring multidimensional cancer genomics data. Cancer Discov 2:401–404 22588877 10.1158/2159-8290.CD-12-0095 PMC3956037

[CR8] Curtis M, Kenny HA, Ashcroft B, Mukherjee A, Johnson A, Zhang Y, Helou Y, Batlle R, Liu X, Gutierrez N et al (2019) Fibroblasts mobilize tumor cell glycogen to promote proliferation and metastasis. Cell Metab 29:141–155.e9 30174305 10.1016/j.cmet.2018.08.007 PMC6326875

[CR9] Davidson CD, Tomczak JA, Amiel E, Carr FE (2022) Inhibition of glycogen metabolism induces reactive oxygen species-dependent cytotoxicity in anaplastic thyroid cancer in female mice. Endocrinology 163:bqac169 36240295 10.1210/endocr/bqac169 PMC10233255

[CR10] de Heer EC, Zois CE, Bridges E, van der Vegt B, Sheldon H, Veldman WA, Zwager MC, van der Sluis T, Haider S, Morita T et al (2023) Glycogen synthase 1 targeting reveals a metabolic vulnerability in triple-negative breast cancer. J Exp Clin Cancer Res 42:143 37280675 10.1186/s13046-023-02715-z PMC10242793

[CR11] Dias D, Oliveira E, Martí-Díaz R, Andrews S, Chocarro-Calvo A, Bellini A, Mosteo L, García YV, Chauhan J, Li L et al (2025) MITF, TFEB, and TFE3 drive distinct adaptive gene expression programs and immune infiltration in melanoma. Cell Rep 44:116499 41275493 10.1016/j.celrep.2025.116499 PMC12828906

[CR12] Falletta P, Goding CR, Vivas-García Y (2022) Connecting metabolic rewiring with phenotype switching in melanoma. Front Cell Dev Biol 10:930250 35912100 10.3389/fcell.2022.930250 PMC9334657

[CR13] Falletta P, Sanchez-Del-Campo L, Chauhan J, Effern M, Kenyon A, Kershaw CJ, Siddaway R, Lisle R, Freter R, Daniels MJ et al (2017) Translation reprogramming is an evolutionarily conserved driver of phenotypic plasticity and therapeutic resistance in melanoma. Genes Dev 31:18–33 28096186 10.1101/gad.290940.116 PMC5287109

[CR14] Favaro E, Bensaad K, Chong MG, Tennant DA, Ferguson DJP, Snell C, Steers G, Turley H, Li JL, Günther UL et al (2012) Glucose utilization via glycogen phosphorylase sustains proliferation and prevents premature senescence in cancer cells. Cell Metab 16:751–764 23177934 10.1016/j.cmet.2012.10.017

[CR15] Ferguson J, Smith M, Zudaire I, Wellbrock C, Arozarena I (2017) Glucose availability controls ATF4-mediated MITF suppression to drive melanoma cell growth. Oncotarget 8:32946–32959 28380427 10.18632/oncotarget.16514 PMC5464841

[CR16] Fogh J, Fogh JM, Orfeo T (1977) One hundred and twenty-seven cultured human tumor cell lines producing tumors in nude mice. J Natl Cancer Inst 59:221–226 327080 10.1093/jnci/59.1.221

[CR17] Gao J, Aksoy BA, Dogrusoz U, Dresdner G, Gross B, Sumer SO, Sun Y, Jacobsen A, Sinha R, Larsson E, et al (2013) Integrative analysis of complex cancer genomics and clinical profiles using the cBioPortal. Sci Signal 6:pl1 10.1126/scisignal.2004088 PMC4160307 23550210

[CR18] García-Jiménez C, Goding CR (2019) Starvation and pseudo-starvation as drivers of cancer metastasis through translation reprogramming. Cell Metab 29:254–267 30581118 10.1016/j.cmet.2018.11.018 PMC6365217

[CR19] Giard DJ, Aaronson SA, Todaro GJ, Arnstein P, Kersey JH, Dosik H, Parks WP (1973) In vitro cultivation of human tumors: establishment of cell lines derived from a series of solid tumors. JNCI J Natl Cancer Inst 51:1417–1423 4357758 10.1093/jnci/51.5.1417

[CR20] Goding CR, Arnheiter H (2019) Mitf—the first 25 years. Genes Dev 33:983–1007 31123060 10.1101/gad.324657.119 PMC6672050

[CR21] Gutiérrez-Salmerón M, García-Martínez JM, Martínez-Useros J, Fernández-Aceñero MJ, Viollet B, Olivier S, Chauhan J, Lucena SR, de la Vieja A, Goding CR et al (2020) Paradoxical activation of AMPK by glucose drives selective EP300 activity in colorectal cancer. PLoS Biol 18:e3000732 32603375 10.1371/journal.pbio.3000732 PMC7326158

[CR22] Gyimesi G, Pujol-Giménez J, Kanai Y, Hediger MA (2020) Sodium-coupled glucose transport, the SLC5 family, and therapeutically relevant inhibitors: from molecular discovery to clinical application. Pflügers Arch 472:1177–1206 32767111 10.1007/s00424-020-02433-x PMC7462921

[CR23] Haldi M, Ton C, Seng WL, McGrath P (2006) Human melanoma cells transplanted into zebrafish proliferate, migrate, produce melanin, form masses and stimulate angiogenesis in zebrafish. Angiogenesis 9:139–151 17051341 10.1007/s10456-006-9040-2

[CR24] Herlyn M et al (1985) Characteristics of cultured human melanocytes isolated from different stages of tumor progression. Cancer Res 45:5670–5676 4053039

[CR25] Jiang L, Liu S, Deng T, Yang Y, Zhang Y (2022) Analysis of the expression, function and signaling of glycogen phosphorylase isoforms in hepatocellular carcinoma. Oncol Lett 24:244 35761940 10.3892/ol.2022.13364 PMC9214699

[CR26] Jin G-Z, Zhang Y, Cong W-M, Wu X, Wang X, Wu S, Wang S, Zhou W, Yuan S, Gao H et al (2018) Phosphoglucomutase 1 inhibits hepatocellular carcinoma progression by regulating glucose trafficking. PLoS Biol 16:e2006483 30335765 10.1371/journal.pbio.2006483 PMC6193743

[CR27] Jose C, Bellance N, Rossignol R (2011) Choosing between glycolysis and oxidative phosphorylation: a tumor’s dilemma?. Biochim Biophys Acta Bioenerg 1807:552–561 10.1016/j.bbabio.2010.10.012 20955683

[CR28] Kami K, Fujimori T, Sato H, Sato M, Yamamoto H, Ohashi Y, Sugiyama N, Ishihama Y, Onozuka H, Ochiai A et al (2013) Metabolomic profiling of lung and prostate tumor tissues by capillary electrophoresis time-of-flight mass spectrometry. Metabolomics 9:444–453 23543897 10.1007/s11306-012-0452-2 PMC3608864

[CR29] Khan T, Sullivan MA, Gunter JH, Kryza T, Lyons N, He Y, Hooper JD (2020) Revisiting glycogen in cancer: a conspicuous and targetable enabler of malignant transformation. Front Oncol 10:592455 33224887 10.3389/fonc.2020.592455 PMC7667517

[CR30] Konieczkowski DJ, Johannessen CM, Abudayyeh O, Kim JW, Cooper ZA, Piris A, Frederick DT, Barzily-Rokni M, Straussman R, Haq R et al (2014) A melanoma cell state distinction influences sensitivity to MAPK pathway inhibitors. Cancer Discov 4:816–827 24771846 10.1158/2159-8290.CD-13-0424 PMC4154497

[CR31] Li Y, Liang R, Sun M, Li Z, Sheng H, Wang J, Xu P, Liu S, Yang W, Lu B et al (2020) AMPK-dependent phosphorylation of HDAC8 triggers PGM1 expression to promote lung cancer cell survival under glucose starvation. Cancer Lett 478:82–92 32171858 10.1016/j.canlet.2020.03.007

[CR32] Liu Q, Li J, Zhang W, Xiao C, Zhang S, Nian C, Li J, Su D, Chen L, Zhao Q et al (2021) Glycogen accumulation and phase separation drives liver tumor initiation. Cell 184:5559–5576.e19 34678143 10.1016/j.cell.2021.10.001

[CR33] Liu Y, Li X, Yang J, Chen S, Zhu C, Shi Y, Dang S, Zhang W, Li W (2024) Pan-cancer analysis of SLC2A family genes as prognostic biomarkers and therapeutic targets. Heliyon 10:e29655 38655365 10.1016/j.heliyon.2024.e29655 PMC11036058

[CR34] Louphrasitthiphol P, Ledaki I, Chauhan J, Falletta P, Siddaway R, Buffa FM, Mole DR, Soga T, Goding CR (2019) MITF controls the TCA cycle to modulate the melanoma hypoxia response. Pigment Cell Melanoma Res 32:792–808 31207090 10.1111/pcmr.12802 PMC6777998

[CR35] Louphrasitthiphol P, Siddaway R, Loffreda A, Pogenberg V, Friedrichsen H, Schepsky A, Zeng Z, Lu M, Strub T, Freter R et al (2020) Tuning transcription factor availability through acetylation-mediated genomic redistribution. Mol Cell 79:472–487.e10 32531202 10.1016/j.molcel.2020.05.025 PMC7427332

[CR36] Ma D, Wang J, Zhao Y, Lee WNP, Xiao J, Go VLW, Wang Q, Recker RR, Xiao GG (2012) Inhibition of glycogen phosphorylation induces changes in cellular proteome and signaling pathways in MIA pancreatic cancer cells. Pancreas 41:397–408 22158071 10.1097/MPA.0b013e318236f022 PMC3306546

[CR37] Marusyk A, Almendro V, Polyak K (2012) Intra-tumour heterogeneity: a looking glass for cancer?. Nat Rev Cancer 12:323–334 22513401 10.1038/nrc3261

[CR38] Mathieu C, Dupret JM, Rodrigues Lima F (2017) The structure of brain glycogen phosphorylase—from allosteric regulation mechanisms to clinical perspectives. FEBS J 284:546–554 27782369 10.1111/febs.13937

[CR39] Matsuzaki H, Shimada S, Uno K, Tsuruta J, Ogawa M (1998) Novel subtyping of intestinal metaplasia in the human stomach: brain-type glycogen phosphorylase expression in the proliferative zone and its relationship with carcinogenesis. Am J Clin Pathol 109:181–189 9583890 10.1093/ajcp/109.2.181

[CR40] Morikawa K, Walker SM, Jessup JM, Fidler IJ (1988a) In vivo selection of highly metastatic cells from surgical specimens of different primary human colon carcinomas implanted into nude mice. Cancer Res 48:1943–1948 3349467

[CR41] Morikawa K, Walker SM, Nakajima M, Pathak S, Jessup JM, Fidler IJ (1988b) Influence of organ environment on the growth, selection, and metastasis of human colon carcinoma cells in nude mice. Cancer Res 48:6863–6871 2846163

[CR42] Müller J, Krijgsman O, Tsoi J, Robert L, Hugo W, Song C, Kong X, Possik PA, Cornelissen-Steijger PDM, Foppen MHG et al (2014) Low MITF/AXL ratio predicts early resistance to multiple targeted drugs in melanoma. Nat Commun 5:5712 25502142 10.1038/ncomms6712 PMC4428333

[CR43] Nowak MA, Fatteh SM, Campbell TE (1998) Glycogen-rich malignant melanomas and glycogen-rich balloon cell malignant melanomas: frequency and pattern of PAS positivity in primary and metastatic melanomas. Arch Pathol Lab Med 122:353–360 9648905

[CR44] Parmenter TJ, Kleinschmidt M, Kinross KM, Bond ST, Li J, Kaadige MR, Rao A, Sheppard KE, Hugo W, Pupo GM et al (2014) Response of BRAF-mutant melanoma to BRAF inhibition is mediated by a network of transcriptional regulators of glycolysis. Cancer Discov 4:423–433 24469106 10.1158/2159-8290.CD-13-0440 PMC4110245

[CR45] Rambow F, Rogiers A, Marin-Bejar O, Aibar S, Femel J, Dewaele M, Karras P, Brown D, Chang YH, Debiec-Rychter M et al (2018) Toward minimal residual disease-directed therapy in melanoma. Cell 174:843–855.e19 30017245 10.1016/j.cell.2018.06.025

[CR46] Ren LK, Lu RS, Fei XB, Chen SJ, Liu P, Zhu CH, Wang X, Pan YZ (2024) Unveiling the role of PYGB in pancreatic cancer: a novel diagnostic biomarker and gene therapy target. J Cancer Res Clin Oncol 150:1–20 10.1007/s00432-024-05644-2 PMC10940407 38483604

[CR47] Sánchez-del-Campo L, Martí-Díaz R, Montenegro MF, González-Guerrero R, Hernández-Caselles T, Martínez-Barba E, Piñero-Madrona A, Cabezas-Herrera J, Goding CR, Rodríguez-López JN (2021) MITF induces escape from innate immunity in melanoma. J Exp Clin Cancer Res 40:117 33789714 10.1186/s13046-021-01916-8 PMC8015040

[CR48] Schnier JB, Nishi K, Monks A, Gorin FA, Bradbury EM (2003) Inhibition of glycogen phosphorylase (GP) by CP-91,149 induces growth inhibition correlating with brain GP expression. Biochem Biophys Res Commun 309:126–134 12943673 10.1016/s0006-291x(03)01542-0

[CR49] Tashima S, Shimada S, Yamaguchi K, Tsuruta J, Ogawa M (2000) Expression of brain-type glycogen phosphorylase is a potentially novel early biomarker in the carcinogenesis of human colorectal carcinomas. Am J Gastroenterol 95:255–263 10638593 10.1111/j.1572-0241.2000.01692.x

[CR50] Tsoi J, Robert L, Paraiso K, Galvan C, Sheu KM, Lay J, Wong DJL, Atefi M, Shirazi R, Wang X et al (2018) Multi-stage differentiation defines melanoma subtypes with differential vulnerability to drug-induced iron-dependent oxidative stress. Cancer Cell 33:890–904.e5 29657129 10.1016/j.ccell.2018.03.017 PMC5953834

[CR51] Verfaillie A, Imrichova H, Atak ZK, Dewaele M, Rambow F, Hulselmans G, Christiaens V, Svetlichnyy D, Luciani F, Van Den Mooter L et al (2015) Decoding the regulatory landscape of melanoma reveals TEADS as regulators of the invasive cell state. Nat Commun 6:1–16 10.1038/ncomms7683 PMC4403341 25865119

[CR52] White RM, Sessa A, Burke C, Bowman T, LeBlanc J, Ceol C, Bourque C, Dovey M, Goessling W, Burns CE et al (2008) Transparent adult zebrafish as a tool for in vivo transplantation analysis. Cell Stem Cell 2:183–189 18371439 10.1016/j.stem.2007.11.002 PMC2292119

[CR53] Yang C, Wang H, Shao M, Chu F, He Y, Chen X, Fan J, Chen J, Cai Q, Wu C (2024) Brain-type glycogen phosphorylase (PYGB) in the pathologies of diseases: a systematic review. Cells 13:289 38334681 10.3390/cells13030289 PMC10854662

[CR54] Zakut R et al (1993) KIT ligand (mast cell growth factor) inhibits the growth of KIT-expressing melanoma cells. Oncogene 8:2221–2229 7687762

[CR55] Zhang C, Quinones A, Le A (2022) Metabolic reservoir cycles in cancer. Semin Cancer Biol 86:180–188 35390455 10.1016/j.semcancer.2022.03.023 PMC9530070

[CR56] Zhang G, Frederick DT, Wu L, Wei Z, Krepler C, Srinivasan S, Chae YC, Xu X, Choi H, Dimwamwa E et al (2016) Targeting mitochondrial biogenesis to overcome drug resistance to MAPK inhibitors. J Clin Invest 126:1834–1856 27043285 10.1172/JCI82661 PMC4855947

[CR57] Zhou Y, Jin Z, Wang C (2019) Glycogen phosphorylase B promotes ovarian cancer progression via Wnt/β-catenin signaling and is regulated by miR-133a-3p. Biomed Pharmacother 120:109449 31627092 10.1016/j.biopha.2019.109449

[CR58] Zois CE, Favaro E, Harris AL (2014) Glycogen metabolism in cancer. Biochem Pharmacol 92:3–11 25219323 10.1016/j.bcp.2014.09.001

[CR59] Zois CE, Harris AL (2016) Glycogen metabolism has a key role in the cancer microenvironment and provides new targets for cancer therapy. J Mol Med 94:137 26882899 10.1007/s00109-015-1377-9 PMC4762924

