# Supplementary material for: Metabolic buffering restricts phenotype switching in melanoma
Source: EMBO J. 2026 Jul 15;45(16):5884–918. doi: 10.1038/s44318-026-00857-2 (PMC13483203; doi:10.1038/s44318-026-00857-2)
Supplement: Supplementary file 1 [file 44318_2026_857_MOESM11_ESM.pdf]

Expanded View Figures

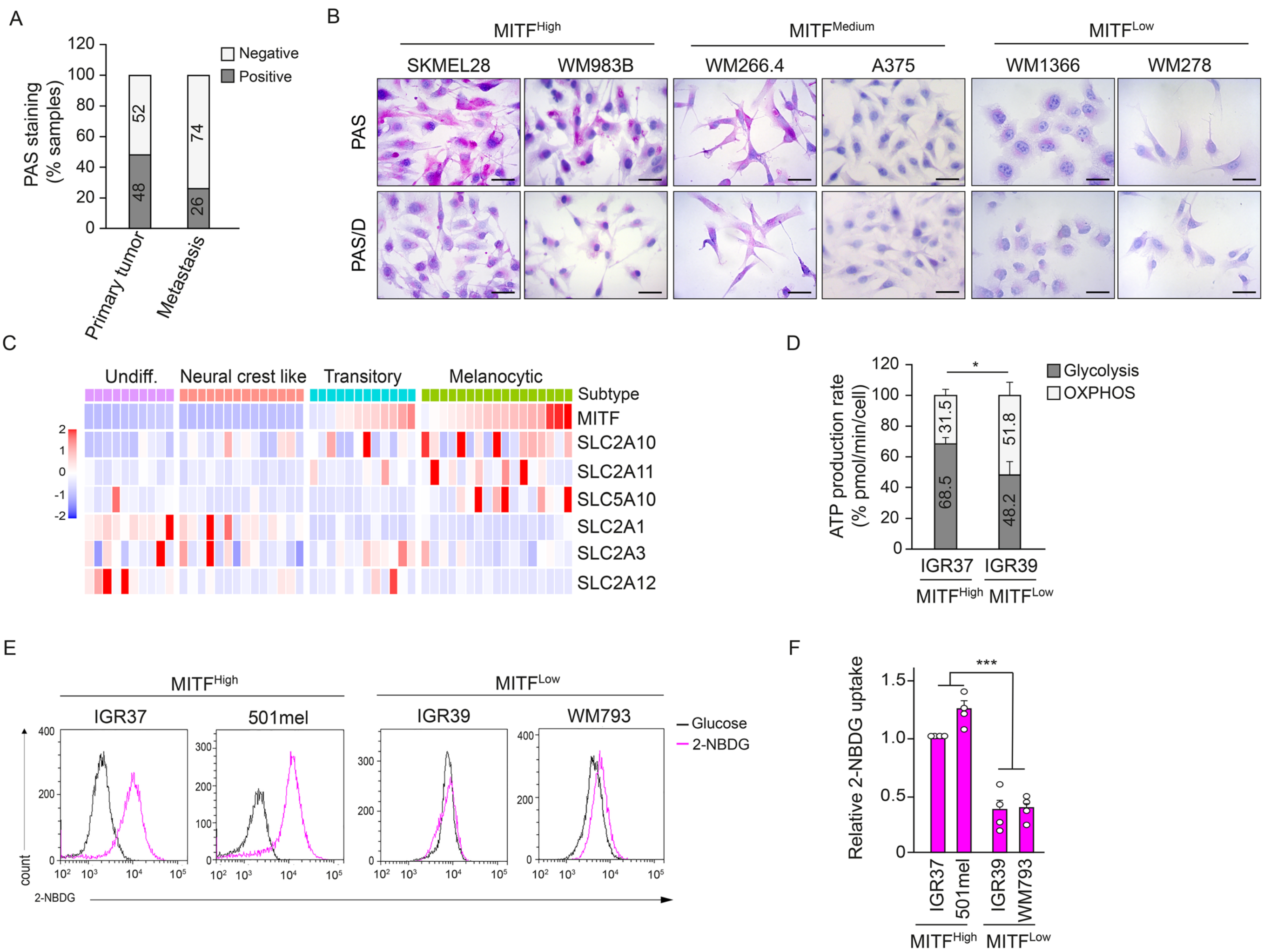

Figure EV1. Glucose uptake and storage in melanoma.

(**A**) Graphic representation of the percentage (%) of samples that store (PAS-Positive) and that do not store glycogen (PAS-Negative) in our collection of melanoma and metastasis biopsies. (**B**) Glycogen stores in SKMEL28 and WM983B (MITF^{High}), WM266.4 and A375 (MITF^{Medium}), and WM1366 and WM278 (MITF^{Low}) melanoma cells were revealed by PAS/PAS-D staining. Representative images, scale bars: 25 µm. (**C**) Heatmap showing relative expression of MITF and glucose transporters: SLC2A10, SLC2A11, SLC5A10, SLC2A1, SLC2A3, and SLC2A12 in the CCLE collection of cell lines classified by phenotype (Tsoi et al, 2018). (**D**) Glycolytic vs mitochondrial oxidative phosphorylation (OXPHOS) production of ATP expressed as percentages, and comparing MITF^{High} (IGR37) and MITF^{Low} (IGR39) melanoma cells. $n = 4$. Data represent mean ± SEM. Statistical analysis by paired *t*-test. $^{*}P < 0.05$. IGR37 vs. IGR39, $P = 0.022$. (**E, F**) Glucose uptake evaluated by flow cytometry in live MITF^{High} (IGR37 and 501mel) and MITF^{Low} (IGR39 and WM793) melanoma cells incubated with 10 µM of the fluorescent glucose analog 2-NBDG for 30 min in the absence of glucose. Representative flow cytometry histograms (**E**) and quantification of mean fluorescence intensity (**F**). $n = 4$. Data represent mean ± SEM. Statistical analysis by one-way ANOVA. $^{*}P < 0.05$; $^{**}P < 0.01$; $^{***}P < 0.001$. Exact *P* values: IGR37 vs. IGR39, $P = 0.0001$; IGR37 vs. WM793, $P = 0.0002$; 501mel vs. IGR39, $P = 0.0001$; 501mel vs. WM793, $P = 0.0001$.

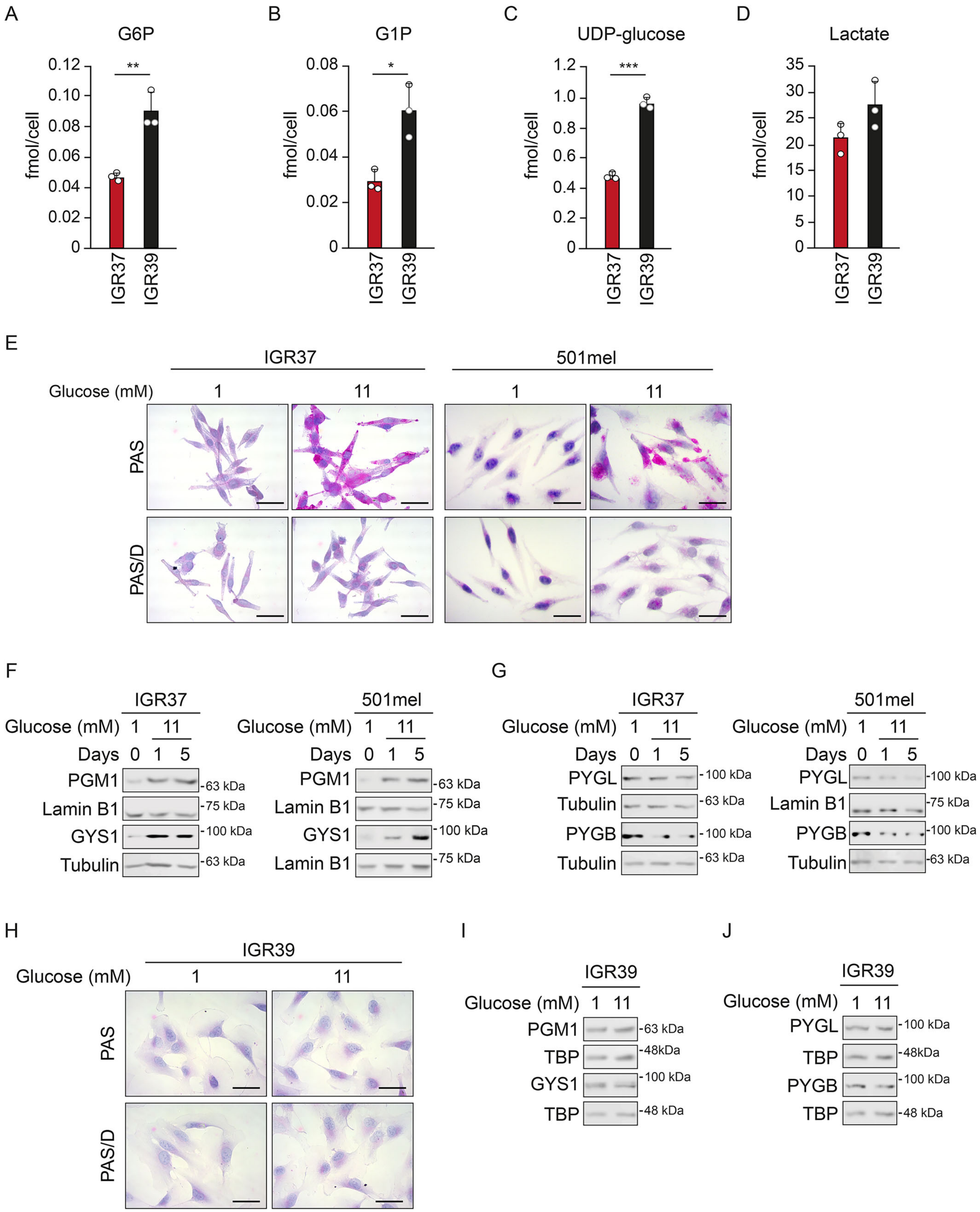
A
G6P
fmol/cell
0.12
0.10
0.08
0.06
0.04
0.02
0
**
IGR37
IGR39
B
G1P
fmol/cell
0.08
0.06
0.04
0.02
0
*
IGR37
IGR39
C
UDP-glucose
fmol/cell
1.2
1.0
0.8
0.6
0.4
0.2
0
***
IGR37
IGR39
D
Lactate
fmol/cell
35
30
25
20
15
10
5
0
IGR37
IGR39
E
IGR37
501mel
Glucose (mM) 1 11 1 11
PAS
PAS/D
F
IGR37
Glucose (mM) 1 11
Days 0 1 5
PGM1 -63 kDa
Lamin B1 -75 kDa
GYS1 -100 kDa
Tubulin -63 kDa
501mel
Glucose (mM) 1 11
Days 0 1 5
PGM1 -63 kDa
Lamin B1 -75 kDa
GYS1 -100 kDa
Lamin B1 -75 kDa
G
IGR37
Glucose (mM) 1 11
Days 0 1 5
PYGL - 100 kDa
Tubulin -63 kDa
PYGB -100 kDa
Tubulin -63 kDa
501mel
Glucose (mM) 1 11
Days 0 1 5
PYGL -100 kDa
Lamin B1 -75 kDa
PYGB -100 kDa
Tubulin -63 kDa
H
IGR39
Glucose (mM) 1 11
PAS
PAS/D
I
IGR39
Glucose (mM) 1 11
PGM1 -63 kDa
TBP -48kDa
GYS1 -100 kDa
TBP -48 kDa
J
IGR39
Glucose (mM) 1 11
PYGL -100 kDa
TBP -48kDa
PYGB -100 kDa
TBP -48 kDa

 © The Author(s)

Figure EV2. Glycogen intermediaries and glycogen dynamics in melanoma cells.

(**A–D**) Metabolomic analysis by capillary electrophoresis–mass spectrometry of steady-state levels of G6P (**A**), G1P (**B**), UDP-glucose (**C**), and lactate (**D**) in IGR37 and IGR39 melanoma cells. $n = 3$ biological replicates. Data represent mean ± standard deviation. Statistical analysis by *t*-test. *$P < 0.05$; **$P < 0.01$; ***$P < 0.001$. Exact *P* values (IGR37 vs. IGR39): G6P, $P = 0.0058$; G1P, $P = 0.0127$; UDP-glucose, $P = 0.0001$; Lactate, $P = 0.1568$. (**E**) Glycogen accumulation by IGR37 and 501mel melanoma cells growing in 1 mM glucose for 24 h and incubated with 11 mM glucose for another 24 h, revealed with PAS (purple)/PAS-D staining. Representative images; scale bars: 25 µm. (**F**, **G**) Western blot analysis of whole cell extracts of indicated cells to compare dynamic changes in the levels of the indicated glycogen metabolism enzymes following glucose addition to the culture (from 1 to 11 mM) for 1 or 5 days. Tubulin or Lamin B as loading control. (**H–J**) Glycogen dynamics in $MITF^{Low}$ IGR39 melanoma cells growing as in (**E**) for comparison. (**H**) Representative images, scale bars: 25 µm. (**I**, **J**) Western blot analysis of IGR39 treated as in (**D**), whole cell extracts; TBP as loading control. Three independent Western blot experiments were performed for each marker.

© The Author(s)

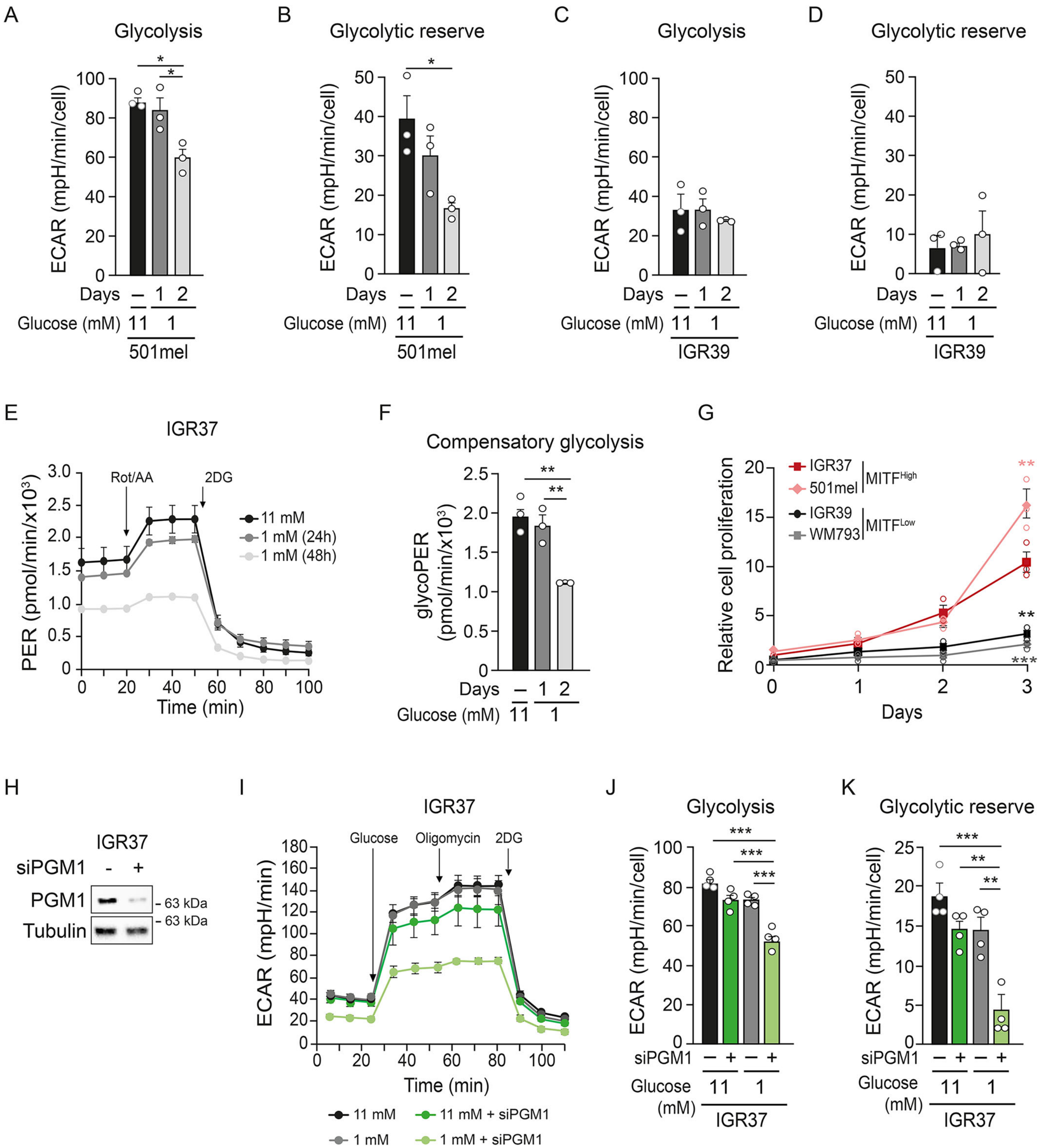
A
Glycolysis
ECAR (mpH/min/cell)
Days – 1 2
Glucose (mM) 11 1
501mel
B
Glycolytic reserve
ECAR (mpH/min/cell)
Days – 1 2
Glucose (mM) 11 1
501mel
C
Glycolysis
ECAR (mpH/min/cell)
Days – 1 2
Glucose (mM) 11 1
IGR39
D
Glycolytic reserve
ECAR (mpH/min/cell)
Days – 1 2
Glucose (mM) 11 1
IGR39
E
IGR37
PER (pmol/min/x10³)
Rot/AA
2DG
11 mM
1 mM (24h)
1 mM (48h)
Time (min)
F
Compensatory glycolysis
glycoPER (pmol/min/x10³)
Days – 1 2
Glucose (mM) 11 1
G
Relative cell proliferation
IGR37
501mel
MITF High
IGR39
WM793
MITF Low
Days
H
IGR37
siPGM1 - +
PGM1
Tubulin
63 kDa
63 kDa
I
IGR37
Glucose
Oligomycin
2DG
ECAR (mpH/min)
Time (min)
11 mM
11 mM + siPGM1
1 mM
1 mM + siPGM1
J
Glycolysis
ECAR (mpH/min/cell)
siPGM1 – + – +
Glucose (mM) 11 1
IGR37
K
Glycolytic reserve
ECAR (mpH/min/cell)
siPGM1 – + – +
Glucose (mM) 11 1
IGR37

 © The Author(s)

Figure EV3. Integrated analysis of glycolytic responses to glucose depletion and PGM1 loss and comparison of proliferation rates for MITF^{High} and MITF^{Low} melanoma cells.

(**A–D**) Seahorse assessment of basal glycolysis (**A**, **C**) and glycolytic reserve (**B**, **D**) derived from the extracellular acidification rate (ECAR) measurement in 501mel (**A**, **B**) and IGR39 (**C**, **D**) melanoma cells. (**E**) Time course of glycolytic proton efflux rate profile (PER) changes of IGR37 melanoma cells depleted of glucose (from 11 to 1 mM) for 1 and 2 days. Mean ± SEM of $n = 3$ independent experiments. (**F**) Quantification of compensatory glycolysis measurement derived from (**E**); (**G**) Proliferation of MITF^{High} (IGR37, 501mel) and MITF^{Low} (IGR39, WM793) melanoma cells growing in 11 mM glucose for 3 days. (**H**) Western blog analysis of whole cell extracts from IGR37 melanoma cells transfected with control or PGM1-specific siRNAs. Tubulin as loading control. (**I**) Extracellular acidification rate (ECAR) of IGR37 melanoma cells depleted of glucose (1 mM) and/or transfected with PGM1 targeting siRNAs for 24 h. (**J**, **K**) Quantification of glycolytic parameters derived from I under indicated conditions: Basal glycolysis (**J**) and Glycolytic reserve (**K**). Statistical analysis by one-way ANOVA (**A–D**, **F**, **G**). $n = 3$ independent experiments (**A–D**, **F**, **G**); $n = 4$ independent experiments (**J**, **K**). Data represent mean ± SEM. *$P < 0.05$; **$P < 0.01$; ***$P < 0.001$. Exact *P* values: (**A**) 11 mM vs. 1 mM 2 days, $P = 0.011$; 1 mM 1 day vs. 2 days, $P = 0,0322$. (**B**) 11 mM vs. 1 mM 2 days, $P = 0.0443$. (**F**) 11 mM vs. 1 mM 2 days, $P = 0.003$; 1 mM 1 day vs. 2 days, $P = 0,0086$. (**G**) IGR37 vs. 501mel, $P = 0,0116$, IGR37 vs. IGR39, $P = 0,0028$, IGR37 vs. WM793, $P = 0,0011$; 501mel vs. IGR39, $P = 0,0001$; 501mel vs. WM793, $P = 0,0001$. (**J**) 11 mM vs. 1 mM + siPGM1, $P = 0.0001$; 11 mM + siPGM1 vs. 1 mM + siPGM1, $P = 0.0001$; 1 mM vs. 1 mM + siPGM1, $P = 0.0001$. (**K**) 11 mM vs. 1 mM + siPGM1, $P = 0.0002$; 11 mM + siPGM1 vs. 1 mM + siPGM1, $P = 0.0038$; 1 mM vs. 1 mM + siPGM1, $P = 0.0038$.

© The Author(s)

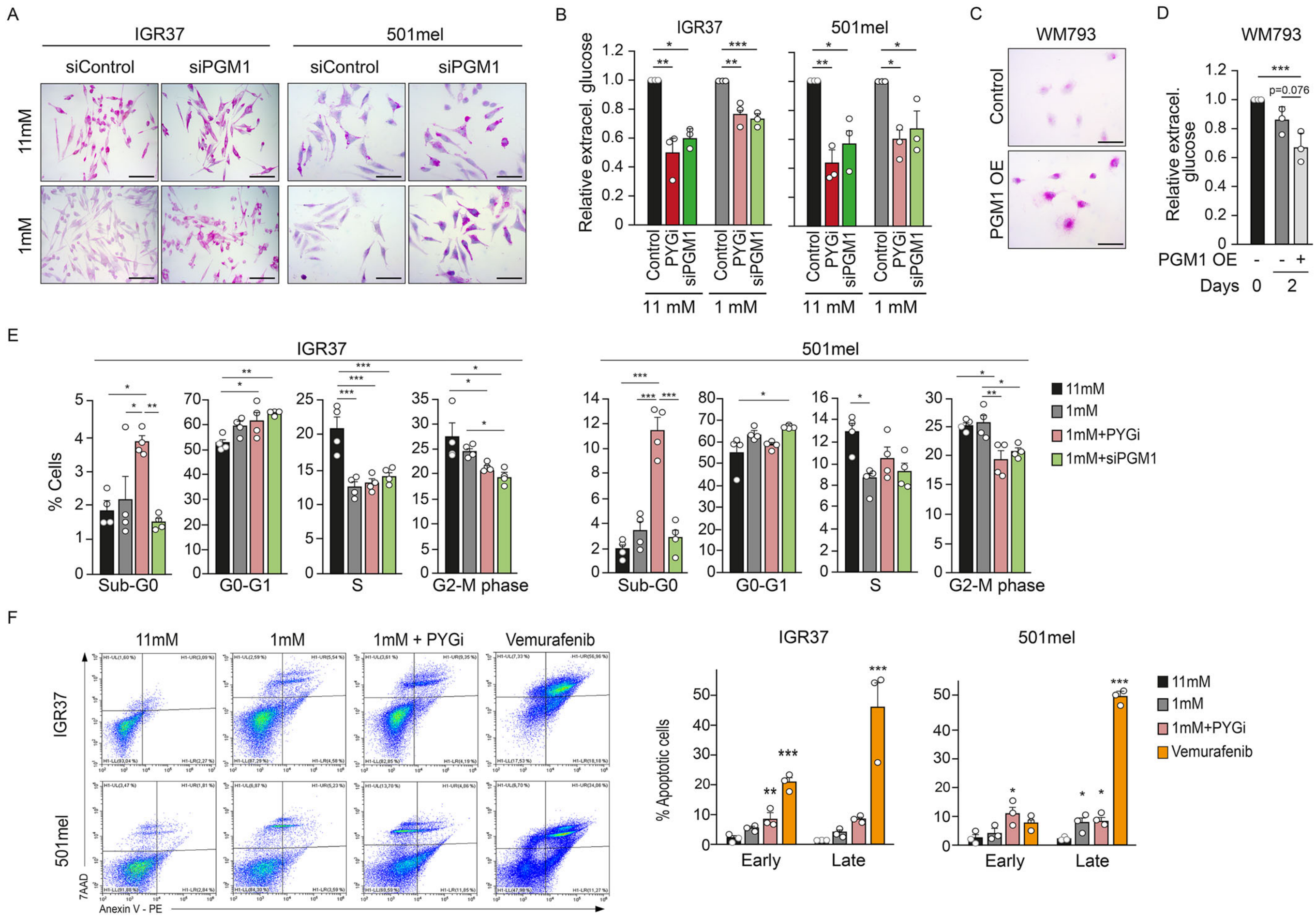

Figure EV4. Glycogen dynamics under conditions that limit glucose availability or glycogen degradation in melanoma cells and effects on cell cycle and cell death.

(**A**) Changes in glycogen content by MITF^{High} (IGR37, 501mel) melanoma cells upon glucose limitation (11 to 1 mM) or limiting glycogen degradation by specific siRNAs transfection targeting PGM1 (or control) for 24 h. Representative images of PAS staining; scale bars: 25 µm. (**B**) Extracellular glucose depletion by IGR37 and 501mel (MITF^{High}) cells upon glucose limitation (from 11 to 1 mM) and/or upon inhibition of glycogen degradation by transfection with PGM1-specific siRNAs or treatment with the glycogen phosphorylase inhibitor CP-91149 (PYGi, 50 µM) for 24 h. (**C**) Changes in glycogen accumulation by MITF^{Low} WM793 melanoma cells cultured in high glucose and after ectopic lentiviral overexpression (OE) of PGM1 or control DNA, for 24 h; representative images of PAS / PAS-D staining; scale bars: 25 µm. (**D**) Changes in extracellular glucose upon PGM1 overexpression (OE) in WM793 melanoma cells. (**E**) Flow cytometry analysis of MITF^{High} (IGR37, 501mel) melanoma cells after limitation of glucose availability. Cells were depleted of glucose (from 11 to 1 mM, 24 h) or depleted of glucose and of PGM1 by siRNAs or treated with the glycogen phosphorylase inhibitor CP-91149 (PYGi, 50 µM) for 48 h. Percentages of cells in Sub-Go, Go-G1, S and G2-M phase are shown; (**F**) Flow cytometry analysis of early and late apoptosis. IGR37 and 501mel melanoma cells were depleted of glucose (from 11 to 1 mM) for 24 h or additionally treated with the glycogen phosphorylase inhibitor CP-91149 (PYGi, 50 µM) for 48 h. Vemurafenib was used as apoptosis positive control. Representative images (left) and quantification (right). Statistical analysis by one-way ANOVA (**B**, **D**, **E**, **F**). $n = 3$ (**B**, **D**, **F**) and $n = 4$ (**E**) biological replicates. Data represent mean ± SEM. *$P < 0.05$; **$P < 0.01$; ***$P < 0.001$. Exact P values: (**B**) For IGR37: 11 mM Control vs. PYGi, $P = 0.0036$; Control vs. siPGM1, $P = 0.0108$; 1 mM Control vs. PYGi, $P = 0.0018$; Control vs. siPGM1, $P = 0.0009$. For 501mel: 11 mM Control vs. PYGi, $P = 0.0029$; Control vs. siPGM1, $P = 0.0107$; 1 mM Control vs. PYGi, $P = 0.0208$; Control vs. siPGM1, $P = 0.0443$. (**D**) Control (−) day 0 vs. PGM1 OE (+) day 2, $P = 0.0006$, PGM1 OE (−) vs. PGM1 OE (+) at day 2, $P = 0.076$. (**E**) For IGR37 in Sub-G0 phase: 11 mM vs. 1 mM+PYGi, $P = 0.0230$; 1 mM vs. 1 mM+PYGi, $P = 0.0306$; 1 mM+PYGi vs. 1 mM+siPGM1, $P = 0.0062$. Go-G1 phase: 11 mM vs. 1 mM+PYGi, $P = 0.0362$; 1 mM vs. 1 mM+siPGM1, $P = 0.0063$. S phase: 11 mM vs. 1 mM, $P = 0.0004$; 11 mM vs. 1 mM+PYGi, $P = 0.0007$; 11 mM vs. 1 mM+siPGM1, $P = 0.0002$. G2-M phase: 11 mM vs. 1 mM+PYGi, $P = 0.0384$; 11 mM vs. 1 mM+siPGM1, $P = 0.0122$; 1 mM vs. 1 mM+siPGM1, $P = 0.0193$. For 501mel in Sub-G0 phase: 11 mM vs. 1 mM+PYGi, $P = 0.0001$; 1 mM vs. 1 mM+PYGi, $P = 0.0001$; 1 mM+PYGi vs. 1 mM+siPGM1, $P = 0.0002$. Go-G1 phase: 11 mM vs. 1 mM+siPGM1, $P = 0.0171$. S phase: 11 mM vs. 1 mM, $P = 0.0340$. G2-M phase: 11 mM vs. 1 mM+PYGi, $P = 0.0121$; 1 mM vs. 1 mM +siPGM1, $P = 0.0320$, 1 mM vs. 1 mM+PYGi, $P = 0.0054$. (**F**) For IGR37 Early apoptosis: 11 mM vs. 1 mM+PYGi, $P = 0.005$ and 11 mM vs. Vemurafenib, $P = 0.0001$; Late apoptosis 11 mM vs. Vemurafenib, $P = 0.0004$. In 501mel Early apoptosis: 11 mM vs. 1 mM+PYGi, $P = 0.0246$; Late apoptosis 11 mM vs. 1 mM, $P = 0.0417$; 11 mM vs. 1 mM +PYGi, $P = 0.0234$ and 11 mM vs. Vemurafenib, $P = 0.0001$.

© The Author(s)

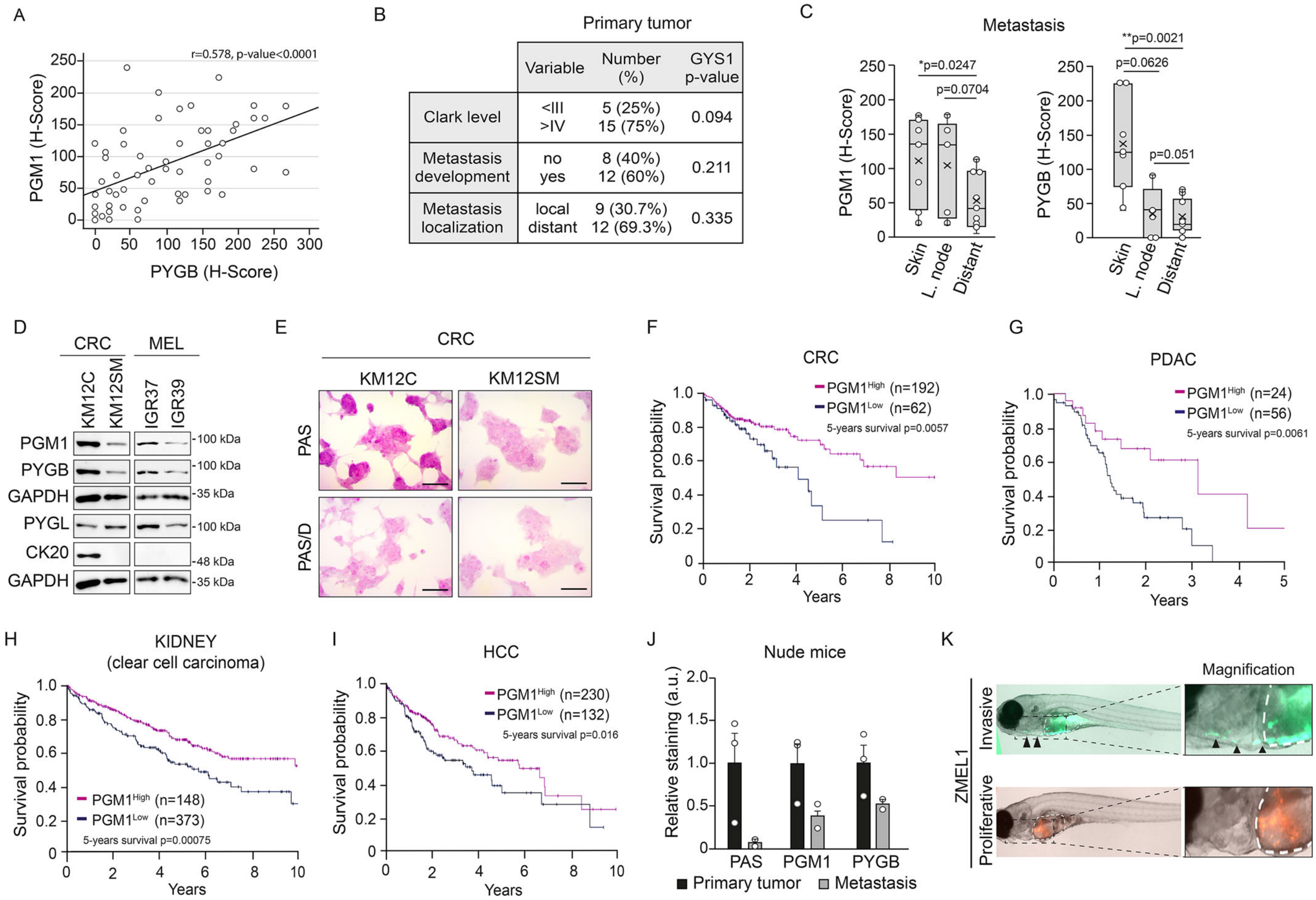

Figure EV5. Relevance of glycogen mobilization enzymes across cancer types.

(**A**) Spearman rank correlation between PGM1 and PYGB protein levels in human melanoma samples. Spearman's rho (*R*) and *P* value are indicated. $*p < 0.05$, $**p < 0.01$, $***p < 0.001$. (**B**) Association analysis between GYS1 H-score and clinicopathological characteristics of patients listed in Table 1. Statistical analysis by the Mann–Whitney *U*-test. Significance threshold set at $P < 0.01$. Exact *P* values for GYS1: Clark level, $P = 0.094$; Metastasis development, $P = 0.211$; metastasis localization, $P = 0.335$. (**C**) H-score analysis of PGM1 and PYGB protein levels in melanoma metastasis samples (Table 1) stratified by distance from the primary tumor (skin, lymph node, or distant). Box plots show median (50%) with lower (25%) and upper (75%) quartiles; whiskers represent minimum and maximum values. Statistical analysis by the Mann–Whitney *U*-test. $*P < 0.05$; $**P < 0.01$; $***P < 0.001$. Exact *P* values: Skin vs. distant: PGM1, $P = 0.0247$; PYGB, $P = 0.0021$. Lymph node vs. distant: PGM1, $P = 0.0704$; PYGB, $P = 0.0051$. Skin vs. lymph node: PYGB, $P = 0.0626$. (**D**) Western blot analysis of whole cell extracts; levels of glycogen-degrading enzymes (PGM1, PYGB, and PYGL) in proliferative versus metastatic phenotypes of colorectal cancer (KM12C and KM12SM) or melanoma (IGR37 vs IGR39) cells. GAPDH as loading control. (**E**) Glycogen content in proliferative KM12C and invasive KM12SM colorectal cancer cell lines revealed by PAS/PAS-D staining. Representative images; scale bars: 25 µm. (**F–I**) Kaplan–Meier curves displaying the estimated survival probability performed using the best cut-off point and data from The Human Protein Atlas for the following cohorts: colorectal cancer (**F**), (CRC): $n = 254$ of which $PGM1^{High}$ ($n = 192$), $PGM1^{Low}$ ($n = 62$), $P = 0.0057$; pancreatic ductal adenocarcinoma (**G**), (PDAC): $n = 80$, $PGM1^{High}$ ($n = 56$), $P = 0.0061$; $PGM1^{Low}$ ($n = 24$); kidney Clear cell carcinoma (**H**), $n = 521$, $PGM1^{High}$ ($n = 148$); $PGM1^{Low}$ ($n = 373$), $P = 0.00075$; and hepatocellular carcinoma (**I**) (HCC): $n = 362$, $PGM1^{High}$ ($n = 132$),; $PGM1^{Low}$ ($n = 230$), $P = 0.016$. $*p < 0.05$, $**p < 0.01$, $***p < 0.001$ (**J**) Quantification of IHC staining of mouse tumor samples: primary and metastasis. The data corresponds to the histoscore profile of glycogen content (PAS), PGM1 and PYGB glycogen-degrading enzymes. (**K**) Larval zebrafish 3 days post-transplant (dpt) of invasive (green) or proliferative (orange) ZMEL1 cells into the yolk sac, representative images. The dotted line indicates the original transplantation site. Unfilled arrowheads indicate instances of dissemination to distal tissue.
