# Supplementary material for: Metabolic buffering restricts phenotype switching in melanoma
Source: EMBO J. 2026 Jul 15;45(16):5884–918. doi: 10.1038/s44318-026-00857-2 (PMC13483203; doi:10.1038/s44318-026-00857-2)
Supplement: Supplementary file 2 [file 44318_2026_857_MOESM1_ESM.pdf]

THE EMBO JOURNAL

Metabolic buffering restricts phenotype switching in melanoma

Ana Ramirez-Sanchez, Miguel Jociles-Ortega, JOSE GARCÍA-MARTÍNEZ, Irene Torrens-Martínez, Javier Martínez-Useros, Pakavarin Louphrasitthiphol, Mollie Sweeney, Ana Morente-Carrasco, Sergio Ruíz-Reyes, Nerea Redondo-Díaz, Teresa Olmos-De Blas, MARÍA FERNÁNDEZ-ACEÑERO, Jose Neptuno Rodriguez-Lopez, Tomoyoshi Soga, Richard White, Colin Goding, Luis Sánchez-del-Campo, CUSTODIA Garcia-Jimenez, and Ana Chocarro-Calvo

Corresponding author(s): Ana Chocarro-Calvo (ana.chocarro@urjc.es) , CUSTODIA Garcia-Jimenez (custodia.garcia@urjc.es)

Review Timeline:

Submission Date:	28th May 25
Editorial Decision:	8th Aug 25
Revision Received:	28th Mar 26
Editorial Decision:	29th Apr 26
Revision Received:	5th Jun 26
Accepted:	26th Jun 26

Editor: William Teale

Transaction Report:

(Note: With the exception of the correction of typographical or spelling errors that could be a source of ambiguity, letters and reports are not edited. Depending on transfer agreements, referee reports obtained elsewhere may or may not be included in this compilation. Referee reports are anonymous unless the Referee chooses to sign their reports.)

1st Editorial Decision **8th Aug 2025**

Dear Dr. Chocarro-Calvo,

Thank you again for the submission of your manuscript entitled "Metabolic buffering suppresses phenotype switching in cancer" and for your patience during the review process. We have now received three referee reports from the referees, which I copy below. Please accept my sincere apologies for the unusually long time this refereeing process took.

As you can see from their comments, all referees specifically noted that your work is timely and exciting. However, all comment that the causal relationship between glycogen and invasiveness implied in the title needs firmer, and perhaps more in vivo, experimental support.

It could be that these experiments will take a considerable time investment to complete. However, I could imagine that they might be feasible if the experimental material has already been prepared or that preparation is well underway. I therefore suggest we discuss the project next week over Zoom. Please, once you and your co-authors have had time to digest the reports, contact me, perhaps with a view to discussing this next Friday. Based on this discussion, we can decide whether the title can be kept as it is, or whether it may have to be softened (which may, depending on the data, result in a manuscript more suitable for EMBO Reports). In the meantime, I will label the manuscript as under revision in our system and supply the relevant revision instructions below my sign off.

Based on the overall interest expressed in the reports, I would like to invite you to address the comments of all referees in a revised version of the manuscript. I should add that it is The EMBO Journal policy to allow only a single major round of revision and that it is therefore important to resolve the main concerns at this stage. I believe the concerns of the referees are reasonable and addressable, but please contact me if you have any questions, need further input on the referee comments or if you anticipate any problems in addressing any of their points. Please, follow the instructions below when preparing your manuscript for resubmission.

I would also like to point out that as a matter of policy, competing manuscripts published during this period will not be taken into consideration in our assessment of the novelty presented by your study ("scooping" protection). We have extended this 'scooping protection policy' beyond the usual 3 month revision timeline to cover the period required for a full revision to address the essential experimental issues. Please contact me if you see a paper with related content published elsewhere to discuss the appropriate course of action.

When preparing your letter of response to the referees' comments, please bear in mind that this will form part of the Review Process File, and will therefore be available online to the community. For more details on our Transparent Editorial Process, please visit our website: https://www.embopress.org/page/journal/14602075/authorguide#transparentprocess

Again, please contact me at any time during revision if you need any help or have further questions.

Thank you very much again for the opportunity to consider your work for publication. I look forward to your revision.

Best regards,

William

William Teale, Ph.D.
Editor
The EMBO Journal

When submitting your revised manuscript, please carefully review the instructions below and include the following items:

1) a .docx formatted version of the manuscript text (including legends for main figures, EV figures and tables). Please make sure that the changes are highlighted to be clearly visible.

2) individual production quality figure files as .eps, .tif, .jpg (one file per figure).

3) a .docx formatted letter INCLUDING the reviewers' reports and your detailed point-by-point response to their comments. As part of the EMBO Press transparent editorial process, the point-by-point response is part of the Review Process File (RPF), which will be published alongside your paper.

4) a complete author checklist, which you can download from our author guidelines (https://wol-prod-cdn.literatumonline.com/pb-

assets/embo-site/Author Checklist%20-%20EMBO%20J-1561436015657.xlsx). Please insert information in the checklist that is also reflected in the manuscript. The completed author checklist will also be part of the RPF.

5) Please note that all corresponding authors are required to supply an ORCID ID for their name upon submission of a revised manuscript.

6) We require a 'Data Availability' section after the Materials and Methods. Before submitting your revision, primary datasets produced in this study need to be deposited in an appropriate public database, and the accession numbers and database listed under 'Data Availability'. Please remember to provide a reviewer password if the datasets are not yet public (see https://www.embopress.org/page/journal/14602075/authorguide#datadeposition). If no data deposition in external databases is needed for this paper, please then state in this section: This study includes no data deposited in external repositories. Note that the Data Availability Section is restricted to new primary data that are part of this study.

Note - All links should resolve to a page where the data can be accessed.

7) When assembling figures, please refer to our figure preparation guideline in order to ensure proper formatting and readability in print as well as on screen:
http://bit.ly/EMBOPressFigurePreparationGuideline

Please remember: Digital image enhancement is acceptable practice, as long as it accurately represents the original data and conforms to community standards. If a figure has been subjected to significant electronic manipulation, this must be noted in the figure legend or in the 'Materials and Methods' section. The editors reserve the right to request original versions of figures and the original images that were used to assemble the figure.

8) For data quantification: please specify the name of the statistical test used to generate error bars and P values, the number (n) of independent experiments (specify technical or biological replicates) underlying each data point and the test used to calculate p-values in each figure legend. The figure legends should contain a basic description of n, P and the test applied. Graphs must include a description of the bars and the error bars (s.d., s.e.m.).

9) We would also encourage you to include the source data for figure panels that show essential data. Numerical data can be provided as individual .xls or .csv files (including a tab describing the data). For 'blots' or microscopy, uncropped images should be submitted (using a zip archive or a single pdf per main figure if multiple images need to be supplied for one panel). Additional information on source data and instruction on how to label the files are available at .

10) We replaced Supplementary Information with Expanded View (EV) Figures and Tables that are collapsible/expandable online (see examples in https://www.embopress.org/doi/10.15252/embj.201695874). A maximum of 5 EV Figures can be typeset. EV Figures should be cited as 'Figure EV1, Figure EV2" etc. in the text and their respective legends should be included in the main text after the legends of regular figures.

- For the figures that you do NOT wish to display as Expanded View figures, they should be bundled together with their legends in a single PDF file called *Appendix*, which should start with a short Table of Content. Appendix figures should be referred to in the main text as: "Appendix Figure S1, Appendix Figure S2" etc. See detailed instructions regarding expanded view here: .

- Additional Tables/Datasets should be labelled and referred to as Table EV1, Dataset EV1, etc. Legends have to be provided in a separate tab in case of .xls files. Alternatively, the legend can be supplied as a separate text file (README) and zipped together with the Table/Dataset file.

11) At EMBO Press we ask authors to provide source data for the main manuscript figures. Our source data coordinator will contact you to discuss which figure panels we would need source data for and will also provide you with helpful tips on how to upload and organize the files.

12) Our journal encourages inclusion of *data citations in the reference list* to directly cite datasets that were re-used and obtained from public databases. Data citations in the article text are distinct from normal bibliographical citations and should directly link to the database records from which the data can be accessed. In the main text, data citations are formatted as follows: "Data ref: Smith et al, 2001" or "Data ref: NCBI Sequence Read Archive PRJNA342805, 2017". In the Reference list, data citations must be labeled with "[DATASET]". A data reference must provide the database name, accession number/identifiers and a resolvable link to the landing page from which the data can be accessed at the end of the reference. Further instructions are available at .

13) In order to increase the reproducibility and reach of your work, The EMBO Journal includes a table of reagents that were used in the study. Please provide this along with your revisions.

Further instructions for preparing your revised manuscript:

Please check that the title and abstract of the manuscript are brief, yet explicit, even to non-specialists.

IMPORTANT: When you send the revision we will require
- a point-by-point response to the referees' comments, with a detailed description of the changes made (as a word file).
- a word file of the manuscript text
- individual production quality figure files (one file per figure)
- a complete author checklist, which you can download from our author guidelines (https://www.embopress.org/page/journal/14602075/authorguide).
- Expanded View files (replacing Supplementary Information)
Please see out instructions to authors
https://www.embopress.org/page/journal/14602075/authorguide#expandedview
- We ask authors to provide source data for the main manuscript figures. You will receive a separate email with instructions for providing source data with your revised manuscript, including how to upload and organize the files.

Please remember: Digital image enhancement is acceptable practice, as long as it accurately represents the original data and conforms to community standards. If a figure has been subjected to significant electronic manipulation, this must be noted in the figure legend or in the 'Materials and Methods' section. The editors reserve the right to request original versions of figures and the original images that were used to assemble the figure.

Further information is available in our Guide For Authors: https://www.embopress.org/page/journal/14602075/authorguide

The revision must be submitted online within 90 days; please click on the link below to submit the revision online before 6th Nov 2025.

https://emboj.msubmit.net/cgi-bin/main.plex

Referee #1:

Ramírez-Sánchez et al. investigate how what they refer to as "metabolic buffering", in this case the ability to store glucose as glycogen and ultimately mobilize those stores, contributes to the switch between different phenotypes in cancer. The authors specifically examine melanoma, which has well-characterized phenotypes of being either proliferative (marked by fast growth and high levels of the transcription factor, MITF) or invasive (characterized by slower growth but higher mobility). Using two cell lines as representatives of each phenotype, they find that the more proliferative (MITFhigh) cell lines accumulate more glycogen than their invasive counterparts (MITFlow), which they show they can use to fuel glycolysis during glucose depleted conditions. The authors also find that the MITFhigh cell lines express higher levels of both key glycogen synthesis and degradation enzymes (with the notable exception of PYGB). In vitro, they find that MITFhigh cell lines have lower rates of relative invasion and, in one cell line, knocking down PGM1 increased invasion. They clearly establish a positive correlation between higher levels of glycogen enzymes and a proliferative phenotype as well as a compelling negative correlation between lower levels of the same enzymes and an invasive phenotype. They provide further evidence for these correlations using a large cohort of melanoma cell lines from the Cancer Genome Atlas, which supports their claims despite weak correlations in this setting. Lastly, they use patient samples of nevi, primary melanoma, and metastatic melanoma to assess the impact of glycogen storage and mobilization on metastasis and survival in a clinical context. By examining gene expression levels, they found that glycogen synthesis/degradation enzymes are upregulated in tumors compared to normal tissue. However, protein staining indicates decreased synthesis enzyme abundance in metastatic tumors versus primary tumors and decreased levels of PYGL in tumors compared to nevi. They also found higher levels of glycogen degradation enzymes are associated with higher Clark levels (a measure of invasiveness) and greater incidence of metastasis, but with more distant metastasis more likely to occur when levels of glycogen degradation enzymes are lower. They find that tumors with higher levels of glycogen degradation enzyme levels are associate with metastasis. However, they also find that lower levels of the same enzymes are more associated with distant metastases while higher levels are associated with local metastases.

Overall the authors report strong associations between glycogen metabolism enzyme expression and proliferative and invasive phenotypes in melanoma. In vitro, the authors provide compelling and interesting data showing clear differences in glycogen metabolism between the two phenotypes. However, there is limited evidence to suggest changes in glycogen metabolism actually drive phenotypic switching as implied by the title. In addition, the associations they find with clinical data are confusing and not always supportive of their cell studies. Some clinical data, such as their finding that the incidence of metastasis is higher in tumors with higher levels of glycogen metabolism and their finding that tumors with higher levels of glycogen enzymes are associated with higher invasion, seem at odds with the cell line findings. These later issues need to be addressed prior to publication.

1. There is minimal data to prove that differences in glycogen metabolism induce phenotype switching. They either need more

work to establish causation, or should adjust the presentation to not make claims about inducing phenotypes. For example, more experiments to test how changes in key glycogen enzymes lead to phenotype switching would be needed to support the existing claims.

2. The associations they report based on clinical data are confusing, and may contradict cell line findings. While both sets of data are compelling, the explanations presented for the discrepancies are somewhat confusing and the story feels incomplete. It seems that the authors expect PGM1 in proliferative tumors to simultaneously be upregulated, as indicated by their in vitro data, and downregulated, as evidenced by their clinical data. Although the authors data does not seem to be mutually exclusive, it would be useful for the authors to make some attempt at a clear reconciliation of their seemingly contradictory findings.

3. To support the general conclusions suggested by the title and abstract, some effort to contextualize glycogen metabolism beyond melanoma is needed, even if only based on correlates within clinical data sets.

Minor Concerns:

1. While interesting, all of the clinical data presented in Figure 5 may not be meaningful. While the gene expression changes are mostly statistically significant, and show trends across enzymes, the significant takeaways from the protein staining data are a bit unclear. Consequently, the authors should be careful about drawing strong conclusions from these data alone.

2. The authors should be careful about how data that is not statistically significant is presented. More care is needed about drawing conclusions from "trends" in such data.

Referee #2:

This manuscript addresses the role of glycogen metabolism in phenotypic switching of melanoma cells under microenvironmental stress. The authors suggest that the capacity to store and mobilize glycogen acts as a metabolic buffer that stabilizes the proliferative phenotype and prevents transition to an invasive state. While the concept of metabolic buffering contributing to phenotypic plasticity is compelling and relevant, I have several concerns regarding novelty, experimental design and the scope of analysis that limit the impact of this study in its present form.

- The limited novelty is one of my major concerns. The link between nutrient (environmental) stress and changes in cancer cell behavior has been studied before. Also, the role of glycogen in regulating cancer cell behavior is not entirely new and has already been reported in other tumor entities such as glioblastoma, hepatocellular carcinoma and ovarian cancer. This work does not clearly add new mechanistic insights as compared with previous reports.

- Some of the presented data are rather correlative and rely on phenotypic associations and expression-level analyses. The conclusions drawn would be significantly strengthened by additional functional assays. Using only crystal violet staining and matrigel-trans well invasion assays is not sufficient to draw strong conclusions. To this end, were the cells that dropped in the lower chamber during the invasion assay calculated? Furthermore, why were cell cycle analyses, 3D spheroid assays, drug sensitivity and other functional assays not performed? Based on the abstract I was expecting such experiments.

- The lack of "in vitro" functional assays might have been compensated by in vivo experiments. However, such investigation has also not been performed. Since the study discusses metastasis and clinical relevance, in vivo data would be helpful.

- The rationale provided for the selection of melanoma cell lines is not sufficient. Given the high degree of inter-line heterogeneity in genetic drivers (e.g., BRAF, NRAS, PTEN status), failure to stratify or account for these factors may have an impact on the described phenotypes. The authors need to clarify whether the observed effects are consistent across lines with different genetic backgrounds.

- While glycogen metabolism is the main focus, many other metabolic pathways can influence cell behavior. The authors do not show whether these other factors were considered or ruled out. This weakens the argument that glycogen is the key driver of the observed effects. The authors might consider altering the title by replacing metabolic buffering with glycogen buffering.

- All figures should include sample sizes and mention the number of biological replicates that were used. This is in particular important for the immunoblots depicted throughout the manuscript.

- The authors might want to consult the following publication: PMID: 9648905, Arch Pathol Lab Med. 1998 Apr;122(4):353-60.

Referee #3:

Ramirez-Sanchez and colleagues provide novel insights that in melanoma, the capacity to store and mobilize glycogen plays a critical role in maintaining the proliferative phenotype and preventing transition to invasion. The authors nicely demonstrate that high glycogen levels are characteristic of proliferative melanoma cells, whereas low glycogen levels are associated with an invasive phenotype. The experimental results show that a loss of glycogen storage/metabolism capacity leads to phenotypic instability and a switch to invasiveness. In addition, they demonstrate that low expression of glycogen phosphorylases and phosphoglucomutase 1 is associated with worse overall survival.

Overall, this is a very well-written and presented manuscript describing an interesting mechanism of metabolic adaptations of cancer cells. The authors present their findings clearly and describe them accurately within the text using imaging approaches.

Below are comments that aim to further increase the clarity and significance of this exciting study:

1. Can the authors shift the metabolic profile of MITF(low) cells by enforcing glycogen accumulation? Is there a difference in proliferation between MITF(low) and MITF(high) melanoma cells? Do MITF(low) and MITF(high) melanoma cells take up the same level of glucose?
2. The authors should provide an overview of the signaling pathways that regulate glycogen synthesis in melanoma.
3. To better assess the metabolic differences between MITF(high) and MITF(low) melanoma cells, the authors should provide direct metabolic measurements of glycogen, G1P, G6P and other glycolytic metabolites.
4. Can the authors provide a mechanistic insight in how MITF(high) melanoma cells mobilize glycogen under glucose-limiting conditions compared to MITF(low) cells lacking this capability. What is regulating the expression of PGM1/PYGB, are these direct target genes of MITF?
5. Do PGM1 knockdown melanoma cells show differences in the glycogen levels, basal/compensatory glycolysis, glycolytic capacity and reserve? Do PGM1 knockdown melanoma cells take up more glucose?
6. Does the loss of PGM1-mediated glycogen metabolism trigger a transcriptional reprogramming that leads to an invasive phenotype in melanoma cells?
7. The increase in invasiveness of the PGM1-depleted 501mel cells should be tested in mice.
8. In Figure 1G/H/I the data for the melanoma cell line WM793 is missing.

Referee #1:

Ramírez-Sánchez et al. investigate how what they refer to as "metabolic buffering", in this case the ability to store glucose as glycogen and ultimately mobilize those stores, contributes to the switch between different phenotypes in cancer. The authors specifically examine melanoma, which has well-characterized phenotypes of being either proliferative (marked by fast growth and high levels of the transcription factor, MITF) or invasive (characterized by slower growth but higher mobility). Using two cell lines as representatives of each phenotype, they find that the more proliferative (MITFhigh) cell lines accumulate more glycogen than their invasive counterparts (MITFlow), which they show they can use to fuel glycolysis during glucose depleted conditions. The authors also find that the MITFhigh cell lines express higher levels of both key glycogen synthesis and degradation enzymes (with the notable exception of PYGB). In vitro, they find that MITFhigh cell lines have lower rates of relative invasion and, in one cell line, knocking down PGM1 increased invasion. They clearly establish a positive correlation between higher levels of glycogen enzymes and a proliferative phenotype as well as a compelling negative correlation between lower levels of the same enzymes and an invasive phenotype. They provide further evidence for these correlations using a large cohort of melanoma cell lines from the Cancer Genome Atlas, which supports their claims despite weak correlations in this setting. Lastly, they use patient samples of nevi, primary melanoma, and metastatic melanoma to assess the impact of glycogen storage and mobilization on metastasis and survival in a clinical context. By examining gene expression levels, they found that glycogen synthesis/degradation enzymes are upregulated in tumors compared to normal tissue. However, protein staining indicates decreased synthesis enzyme abundance in metastatic tumors versus primary tumors and decreased levels of PYGL in tumors compared to nevi. They also found higher levels of glycogen degradation enzymes are associated with higher Clark levels (a measure of invasiveness) and greater incidence of metastasis, but with more distant metastasis more likely to occur when levels of glycogen degradation enzymes are lower. They find that tumors with higher levels of glycogen degradation enzyme levels are associate with metastasis. However, they also find that lower levels of the same enzymes are more associated with distant metastases while higher levels are associated with local metastases.

Overall the authors report strong associations between glycogen metabolism enzyme expression and proliferative and invasive phenotypes in melanoma. In vitro, the authors provide compelling and interesting data showing clear differences in glycogen metabolism between the two phenotypes. However, there is limited evidence to suggest changes in glycogen metabolism actually drive phenotypic switching as implied by the title. In addition, the associations they find with clinical data are confusing and not always supportive of their cell studies. Some clinical data, such as their finding that the incidence of metastasis is higher in tumors with higher levels of glycogen metabolism and their finding that tumors with higher levels of glycogen enzymes are associated with higher invasion, seem at odds with the cell line findings. These later issues need to be addressed prior to publication.

1. There is minimal data to prove that differences in glycogen metabolism induce phenotype switching. They either need more work to establish causation, or should adjust the presentation to not make claims about inducing phenotypes. For example, more experiments to test how changes in key glycogen enzymes lead to phenotype switching would be needed to support the existing claims.

We fully agree that establishing causality requires robust functional validation, and we have addressed this concern through substantial new experimental data and by revising the manuscript text to avoid overstating causal claims. To strengthen the functional link between glycogen metabolism and phenotype switching, we have added multiple independent lines of evidence:

Loss- and gain-of-function experiments with PGM1. PGM1 depletion, which impairs glycogen mobilization, consistently increases invasive capacity in both 501mel and WM266.4 cells (new Fig. 4E–F). Critically, transitory overexpression of PGM1 in the invasive WM793 line, which does not accumulate glycogen, was sufficient to promote glycogen deposition and significantly reduces invasive capacity (new Fig. 4G–H). This direct gain-of-function evidence demonstrates that PGM1 activity causally restrains the invasive phenotype.

Convergent pharmacological perturbations. Inhibition of glycogen phosphorylase (an enzyme that promotes glycogen mobilization) and glucose deprivation, both enhance invasiveness in 501mel and WM266.4 cells (new Fig. 5A) and potentiate invasive outgrowth in 3D spheroid assays (new Fig. 5B). The convergence of independent perturbations on the same phenotype substantially strengthens a causal interpretation.

Coordinated phenotypic shift. Conditions that reduce glycogen availability also impair adhesion and colony-forming capacity (new Fig. 5C–D), consistent with a coordinated transition toward a less proliferative, more motile state rather than isolated changes in individual assays.

Clinically relevant experiment. A375 cells that acquired vemurafenib resistance showed concomitant MITF re-expression, upregulation of PGM1 and PYGL, increased glycogen accumulation, and reduced invasive capacity (Fig. 6I–M). This spontaneous co-occurrence of metabolic and phenotypic reprogramming, though correlative, further supports the notion that elevated glycogen metabolism capacity actively suppresses the invasive phenotype.

Taken together, these in vitro data demonstrate that diverse, independent perturbations of glycogen metabolism reproducibly promote invasive traits, while restoration of glycogen metabolism suppresses them. We have revised the manuscript text throughout to adopt appropriately conservative language regarding causality while reflecting the strength of the converging functional evidence. We also now provide in vivo data (see below) to support the role of glycogen metabolism in controlling melanoma invasiveness.

2. The associations they report based on clinical data are confusing and may contradict cell line findings. While both sets of data are compelling, the explanations presented for the discrepancies are somewhat confusing and the story feels incomplete. It seems that the authors expect PGM1 in proliferative tumors to simultaneously be upregulated, as indicated by their in vitro data, and downregulated, as evidenced by their clinical data. Although the authors data does not seem to be mutually exclusive, it would be useful for the authors to make some attempt at a clear reconciliation of their seemingly contradictory findings.

We agree that the relationship between our experimental and clinical data required clearer explanation, and we have substantially revised the manuscript to present an explicit reconciling framework.

Our model proposes that glycogen functions as a metabolic reservoir that under glucose limitation delays the transition from a proliferative to an invasive phenotype. Proliferative $MITF^{High}$ cells express high levels of glycogen metabolism enzymes and actively cycle glycogen, synthesizing it under nutrient-replete conditions and mobilizing it under stress. As tumors progress toward invasion and metastasis, this capacity is progressively lost. The apparent discrepancy between in vitro and clinical data arises because each dataset captures a different contrast within this continuum: gene expression analyses compare non-tumor tissue with tumor or metastatic samples, whereas H-score analyses compare nevi with primary and metastatic tumors. Additionally, mRNA and protein levels can diverge substantially due to post-transcriptional regulation, allosteric modulation, and rapid metabolic adaptation, as discussed in the revised text (lines 413-433). Once these distinctions are made explicit, the two bodies of evidence are fully coherent rather than contradictory.

As mentioned above, in vitro, PGM1 depletion impairs glycogen mobilization and increases invasiveness (new Fig. EV4A, 4E–F), while PGM1 overexpression in invasive cells restores glycogen accumulation and reduces invasiveness (new Fig. 4G–H). These results establish that glycogen mobilization capacity restrains the invasive phenotype.

Primary tumors with higher metastatic potential (Clark IV) display lower glycogen levels alongside elevated PGM1 and PYGB expression (new Fig. 8A–C), consistent with active glycogen mobilization and depletion, not passive accumulation. Furthermore, primary tumors associated with local metastases show higher PGM1 and PYGB expression than those associated with distant metastases (new Fig. 8E), suggesting that glycogen mobilization supports early migratory steps but is progressively lost as cells colonize distant organs. This trajectory is recapitulated in our murine allograft data, where matched lung metastases exhibited significantly lower PAS staining and reduced PGM1 and PYGB expression compared with their corresponding primary tumors (new Fig. EV5J). Notably, lymph node metastases retain PGM1 expression (new Fig. EV5C),

whereas distant metastases do not, consistent with a post-metastatic metabolic adaptation in which sustained glycogen mobilization becomes dispensable or unsustainable.

Kaplan–Meier analysis of melanoma patient samples (new Fig. 8F–G) shows that high expression of glycogen-degrading enzymes associates with improved survival, consistent with the interpretation that active glycogen metabolism capacity reflects a more controlled disease course. This relationship extends to colorectal, pancreatic, kidney clear cell carcinoma, and hepatocellular carcinoma (new Fig. EV5F–I), suggesting it reflects a broadly conserved principle linking glycogen mobilization capacity to tumor aggressiveness.

We hope this revised explanation, together with the reorganized figures and new data, clarifies the coherence between our in vitro and clinical findings and addresses the reviewer's concerns.

3. To support the general conclusions suggested by the title and abstract, some effort to contextualize glycogen metabolism beyond melanoma is needed, even if only based on correlates within clinical data sets.

We thank the reviewer for this suggestion and have expanded the manuscript to contextualize our findings beyond melanoma through both new experimental data, cross-cancer clinical analyses, and an extended Discussion.

We examined the KM12C colorectal cancer progression model, comprising the parental KM12C line and its spontaneous hepatic metastasis-derived subline KM12SM (Morikawa et al., 1988a, 1988b). Western blot analysis revealed that KM12C cells express high levels of glycogen metabolism enzymes, including PGM1 and PYG isoforms, and accumulate substantial intracellular glycogen as evidenced by PAS staining. In contrast, the more aggressive KM12SM subline exhibited markedly lower expression of glycogen mobilization enzymes and negligible glycogen content (new Fig. EV5D–E). This inverse relationship between metastatic aggressiveness and glycogen mobilization capacity directly mirrors our melanoma findings, supporting the notion that impaired glycogen mobilization is a broader correlate of metastatic potential.

We extended our survival analysis to multiple independent cohorts. Kaplan–Meier analyses in colorectal cancer (CRC), pancreatic ductal adenocarcinoma (PDAC), kidney clear cell carcinoma and hepatocellular carcinoma (HCC) consistently show that high PGM1 expression associates with improved patient outcomes (new Fig. EV5F–I), recapitulating the relationship observed in melanoma. The consistency of this association across four tumor types with distinct biology, tissue of origin and metastatic patterns suggests that the prognostic relevance of glycogen mobilization capacity extends well beyond melanoma.

Finally, we have expanded the Discussion to place our findings within the wider cancer glycogen metabolism literature. Dysregulated glycogen storage or mobilization have been reported across a range of malignancies, including kidney, liver, ovarian, breast, lung, bladder, gastric, colorectal, prostate cancer and glioblastoma, where alterations in glycogen synthase and phosphorylase activity have been linked to tumor progression. Although these studies vary in tumor type, methodology and metastatic context, they collectively support the generalizability of our mechanistic findings and justify the broader framing reflected in the title and abstract.

Minor Concerns:

1. While interesting, all of the clinical data presented in Figure 5 may not be meaningful. While the gene expression changes are mostly statistically significant, and show trends across enzymes, the significant takeaways from the protein staining data are a bit unclear. Consequently, the authors should be careful about drawing strong conclusions from these data alone.

We agree that the protein staining data should be interpreted carefully and consequently we have revised the manuscript in several ways to address this concern.

We have softened the language throughout the Results and Discussion so that the immunohistochemical and PAS staining data are consistently presented as supportive and correlative evidence rather than definitive proof. Assertive language has been replaced with appropriately hedged language, such as "consistent with", "suggestive of", "tended to" and "showed a non-significant trend toward", wherever the protein data do not reach statistical significance or where sample sizes are limited.

We have added an explicit acknowledgment that mRNA and protein levels can diverge substantially due to post-transcriptional regulation, allosteric modulation and rapid metabolic adaptations that occur independently of transcriptional changes. This is particularly relevant for glycogen metabolism enzymes, whose activity is subject to tight allosteric and hormonal control, and is discussed in the context of the GYS1 and PGM1 protein/mRNA discrepancies observed between primary tumors and metastases (Results, lines 419-427).

We have ensured that no major conclusion of the manuscript rests solely on the protein staining data. Each key claim is supported by at least one independent line of functional evidence, including PGM1 loss- and gain-of-function experiments, pharmacological perturbation of glycogen metabolism, metabolomic profiling, and in vivo models in mice and zebrafish. The clinical staining data are therefore presented as consistent with, rather than proof of, the mechanistic framework established by these functional analyses.

We believe these revisions appropriately contextualize the clinical data and ensure that conclusions are drawn with the necessary caution.

2. The authors should be careful about how data that is not statistically significant is presented. More care is needed about drawing conclusions from "trends" in such data.

Again, we fully agree that non-significant differences should not be overinterpreted. We have revised the manuscript carefully to address this concern at multiple levels.

We have systematically reviewed the Results and Discussion and replaced any language implying definitive conclusions from non-significant trends with appropriately hedged phrasing such as "showed a tendency toward", "did not reach statistical significance", "consistent with, though not significant", and "suggestive of". Claims that previously relied solely on non-significant differences to support major conclusions have been either removed or reframed as preliminary observations requiring further validation.

We now explicitly state when differences do not reach statistical significance directly in the Results text, rather than leaving this implicit or relegating it to figure legends alone. For example, the protein-level changes in GYS1 between nevi and primary tumors, and in PGM1 between primary tumors and metastases, are now described as non-significant trends, with the caveat that limited sample sizes may reduce statistical power in these comparisons.

All figure legends have been updated to include exact p-values, sample sizes and the statistical tests used for every comparison, including those described as trends, allowing readers to independently evaluate the strength of each observation.

Finally, we have ensured that no major conclusion of the manuscript rests solely on non-significant data. Each key claim is supported by at least one statistically significant result or an independent functional experiment, so that non-significant trends serve only as corroborating context rather than primary evidence.

Referee #2:

This manuscript addresses the role of glycogen metabolism in phenotypic switching of melanoma cells under microenvironmental stress. The authors suggest that the capacity to store and mobilize glycogen acts as a metabolic buffer that stabilizes the proliferative phenotype and prevents transition to an invasive state. While the concept of metabolic buffering contributing to phenotypic plasticity is compelling and relevant, I have several concerns regarding novelty, experimental design and the scope of analysis that limit the impact of this study in its present form.

- The limited novelty is one of my major concerns. The link between nutrient (environmental) stress and changes in cancer cell behavior has been studied before. Also, the role of glycogen in regulating cancer cell behavior is not entirely new and has already been reported in other tumor entities such as glioblastoma, hepatocellular carcinoma and ovarian cancer. This work does not clearly add new mechanistic insights as compared with previous reports.

We thank the reviewer for highlighting prior work linking nutrient stress, glycogen biology and cancer behavior. We fully agree that glycogen metabolism has been implicated in several tumor types; however, our study advances the field in several substantive ways not addressed in previous reports, and we have revised the manuscript to make these contributions more explicit.

Our work provides a phenotype-resolved and mechanistically integrated analysis. While glycogen has been studied in glioblastoma, hepatocellular carcinoma, and ovarian cancer, none of these studies examined glycogen metabolism in the context of defined phenotypic states or phenotype switching, nor did they integrate temporal metabolic perturbations with lineage-specific transcriptional programs. Melanoma is uniquely suited for this analysis because its proliferative and invasive phenotypes are molecularly well-defined, enabling a precision of dissection that is difficult to achieve in less characterized tumor models.

We introduce a new mechanistic concept: glycogen as a temporal metabolic buffer. We introduce the concept that glycogen acts not merely as a fuel source but as a temporal buffer that stabilizes the proliferative MITF^{High} state under glucose limitation stress. In our model, MITF^{High} cells encountering low-glucose conditions mobilize glycogen stores to delay the decision to switch from proliferation to invasion, a switch otherwise driven by glucose-mediated activation of the integrated stress response, which translationally and transcriptionally suppresses MITF expression. When glycogen cannot be mobilized, either genetically or pharmacologically, cells prematurely transition to an invasive phenotype. MITF^{Low} cells, which lack glycogen stores, have no such buffer and are therefore constitutively invasive. This buffering model and its integration with MITF-dependent regulatory circuitry has not been described previously in any tumor context.

Our conclusions are supported by a level of experimental triangulation not assembled in prior studies, combining: (i) time-resolved in vitro perturbations including PGM1 loss- and gain-of-function, glucose withdrawal, and pharmacological glycogen phosphorylase inhibition (new Fig. 5); (ii) complementary in vivo models including murine allografts and zebrafish larval transplantation, where (PYG inhibitor) PYGi in proliferative ZMEL1 cells phenocopied the invasive state (new Fig. 9 and EVD5K); (iii) a colorectal cancer progression model (KM12C/KM12SM) independently recapitulating the inverse relationship between glycogen mobilization capacity and metastatic aggressiveness (new Fig. EV5D–E); and (iv) cross-cancer Kaplan–Meier survival analyses across colorectal, pancreatic, kidney, and hepatocellular carcinoma cohorts (new Fig. EV5F–I). This triangulation substantially strengthens causal inference and generalizability beyond single-model observations.

We provide an integrative interpretation that reconciles apparent discrepancies between mRNA, protein, and metastatic-site-specific data. By combining functional experiments with patient-derived tissue microarrays and survival analyses, we demonstrate how glycogen mobilization, depletion and post-metastatic metabolic adaptation generate distinct molecular readouts at different stages of tumor progression, an interpretation not proposed in previous studies, and one that helps explain why glycogen-related signatures appear contradictory when examined in isolation.

Together, these contributions position our work not as an incremental extension of prior glycogen studies but as a mechanistically grounded, clinically validated framework for understanding how metabolic buffering shapes phenotypic plasticity in cancer.

- Some of the presented data are rather correlative and rely on phenotypic associations and expression-level analyses. The conclusions drawn would be significantly strengthened by additional functional assays. Using only crystal violet staining and matrigel-transwell invasion assays is not sufficient to draw strong conclusions. To this end, were the cells that dropped in the lower chamber during the invasion assay calculated? Furthermore, why were cell cycle analyses, 3D spheroid assays, drug sensitivity and other functional assays not performed? Based on the abstract I was expecting such experiments.

We agree that functional validation beyond crystal violet staining and Matrigel transwell assays is essential to support our conclusions and therefore we have substantially expanded the functional evidence in the revised manuscript.

Transwell invasion quantification. Cells that migrated to the lower chamber were counted in all transwell invasion assays. These raw counts reproduce the same conclusions shown in the invasion graphs; although they introduce some inter-experiment variability (e.g., control wells ranging from ~600 to ~1,000 cells, reviewer Figure 1), they do not alter the statistical interpretation.

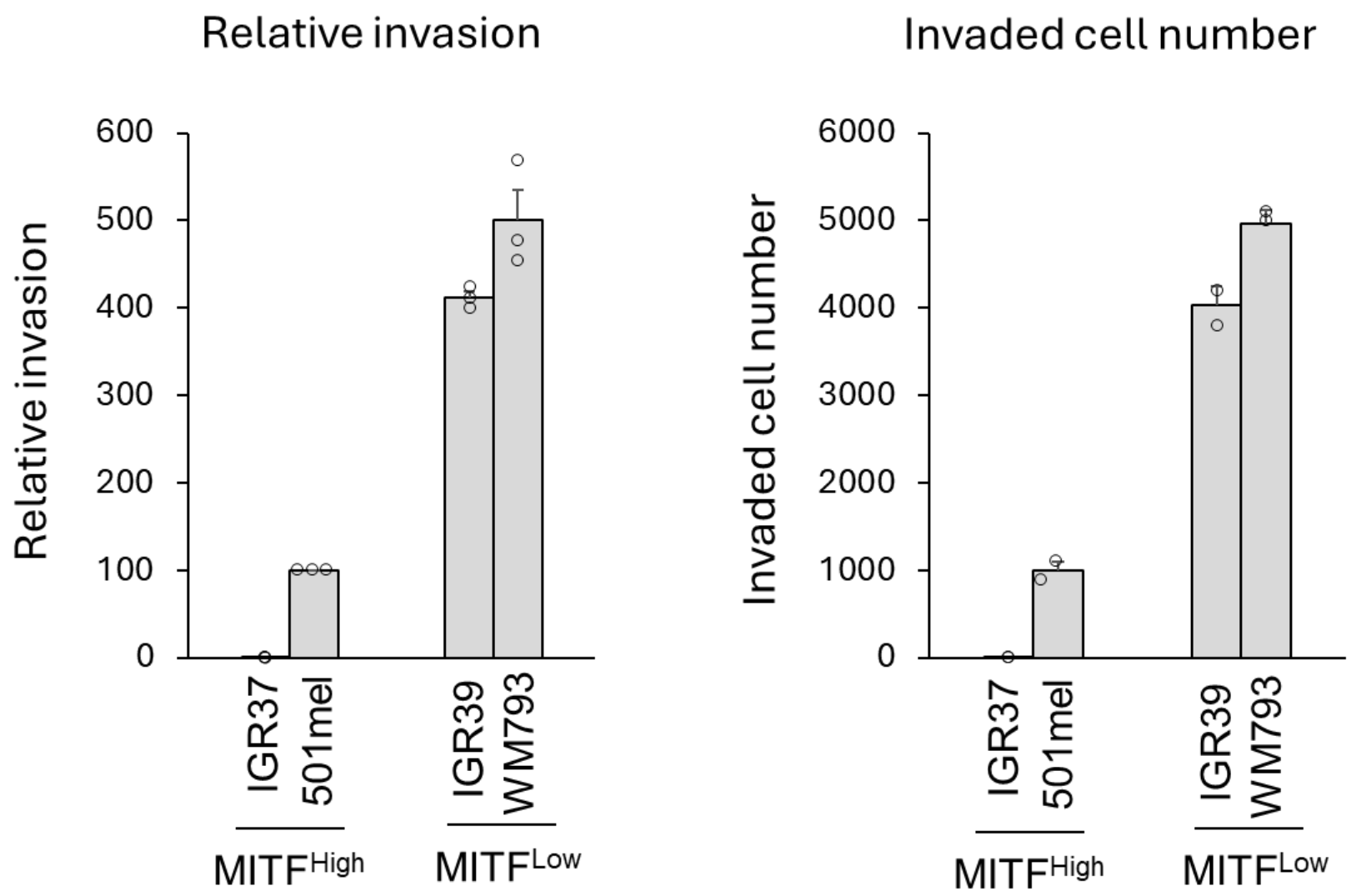

Reviewer Figure 1

Choice of crystal violet proliferation assays. We used crystal violet staining because it avoids the confounding metabolic readouts inherent to MTT/XTT assays, a particularly important consideration given that our experimental conditions directly perturb cellular metabolism. Crystal violet provides an integrated measure of cell number over timescales appropriate for phenotype shifts that unfold over more than 24 hours. Nevertheless, we acknowledge the limitations of relying on this assay alone and have added multiple functional assays as described below.

Additional functional assays. To address the reviewer's concern, we have incorporated the following:

- *3D spheroid invasion assays (new Fig. 5D):* Prolonged glucose depletion or pharmacological inhibition of glycogen degradation with the PYG inhibitor CP-94119 increased invasive outgrowth of WM266.4 spheroids, confirming that impaired access to glucose reserves drives a pro-invasive phenotypic shift in a three-dimensional model that better recapitulates tumor architecture.

- *Cell adhesion assays (new Fig. 5E):* Glucose depletion or PYGi (CP-91441) treatment reduced adhesion capacity in IGR37, 501mel, and WM266.4 cells, consistent with a coordinated shift toward a less proliferative, more motile phenotype.

- *Clonogenic assays (new Fig. 5F):* Colony formation was significantly impaired under glucose-limiting conditions and further reduced upon PYGi (CP-91441), corroborating the proliferative-to-invasive shift at the level of long-term growth capacity.

- *Cell cycle analysis (new Fig. EV4C):* Glucose deprivation reduced the S-phase fraction and promoted G0–G1 accumulation in $MITF^{High}$ cells. PGM1 knockdown or PYGi (CP-91441) treatment potentiated these effects and further decreased the G2–M population, consistent with metabolic stress-induced cell cycle arrest rather than cytotoxicity.

- *Annexin V apoptosis assays (new Fig. EV4D):* Annexin V-PE/7-AAD staining confirmed that glucose depletion combined with PYGi induced only a modest but statistically significant increase in early apoptosis, substantially lower than that induced by vemurafenib used as a positive control. This confirms that the observed phenotypic effects are not primarily driven by cell death.

Drug sensitivity. To provide context for the cell lines used in this study, we include IC50 values for vemurafenib across our melanoma panel as a Reviewer Figure (Reviewer Figure 2). These data confirm that the lines span a range of drug sensitivities consistent with their known proliferative and invasive phenotypic states, in agreement with published pharmacological profiles. These data are provided for transparency and are not proposed for inclusion in the manuscript.

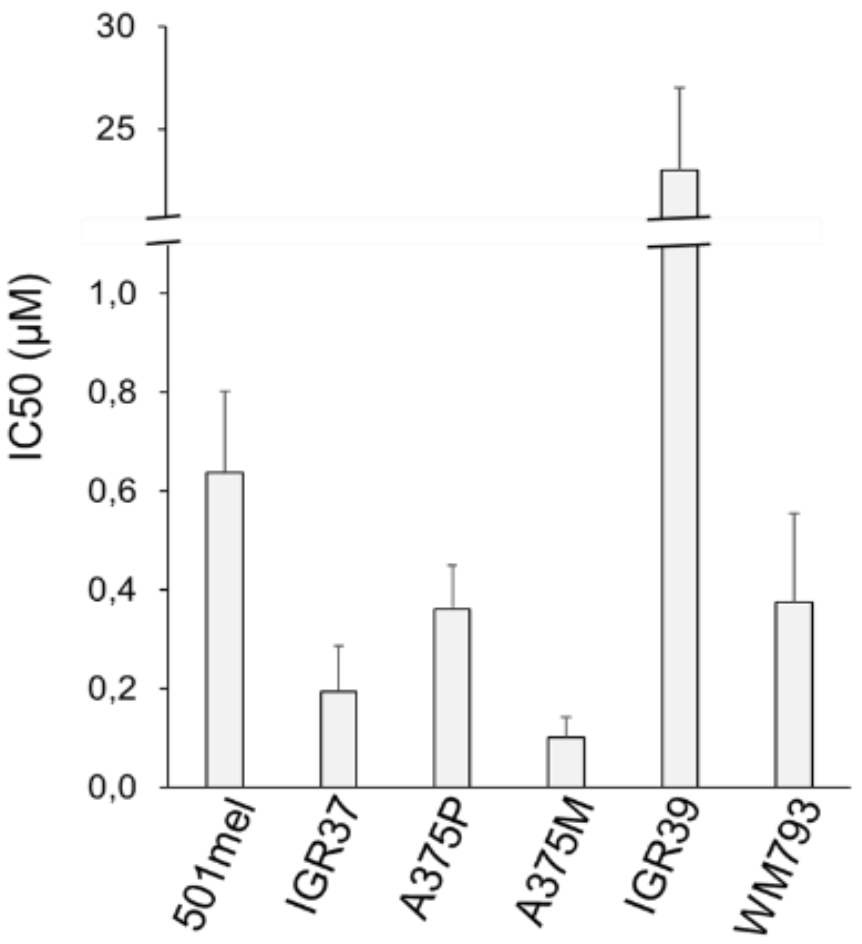

Reviewer Figure 2

Together, these additions substantially reduce reliance on any single assay type and provide robust, multimodal functional support for the role of glycogen metabolism in regulating melanoma phenotype switching.

- The lack of "in vitro" functional assays might have been compensated by in vivo experiments. However, such investigation has also not been performed. Since the study discusses metastasis and clinical relevance, in vivo data would be helpful.

We thank the reviewer for this important point and are pleased to report that the revised manuscript now includes substantial in vivo evidence directly addressing metastatic behaviour across two independent animal models, presented in new Figure 9.

Murine allograft model. B16-F10 melanoma cells were implanted subcutaneously into athymic nude mice. This model was chosen due to the inability of B16 cells to metastasize in C57/Bl6 mice and to avoid confusion by effects on immune system. Nude mice were treated with the glycogen phosphorylase inhibitor CP-91441 (PYGi) or vehicle once tumors reached a palpable size. Vehicle-treated mice developed significantly larger primary tumors and overt metastases, whereas PYGi-treated animals showed reduced primary tumor growth and failed to develop metastases (new Fig. 9B–C). This might be attributed to a limitation in the invasive capacity of melanoma cells in the primary tumor or to systemic blockade of glycogen phosphorylase activity, which may limit glycogen bioavailability in metastatic target organs such as the liver (the largest glycogen reservoir in the body) rendering these tissues less permissive to tumor colonization or tumor cell proliferation. Histological and immunohistochemical analysis confirmed that PYGi treatment reduced intracellular glycogen content and upregulated PGM1 and PYGB expression in primary tumors, mirroring the metabolic profile of highly invasive Clark stage IV human tumors (new Fig. 9D–E). Critically, when untreated primary tumors were stratified by metastatic outcome, those from mice that developed metastases exhibited significantly higher PAS staining and elevated PGM1 and PYGB levels compared with non-metastatic tumors (new Fig. 9F), recapitulating the pattern observed in human primary melanoma. Matched lung metastases showed markedly lower PAS staining and reduced enzyme expression compared with their corresponding primary tumors (new Fig. EV5J), mirroring the stage-dependent loss of glycogen metabolic capacity observed in human metastatic samples.

Zebrafish larval transplantation model. To directly visualize early tumor cell invasion in vivo at single-cell resolution, we employed the optically transparent *casper* zebrafish larval model. Fluorescently labelled ZMEL1 proliferative and invasive cells were microinjected into the Duct of Cuvier of *casper* larvae at 2 dpf and treated with PYGi or vehicle. As expected, invasive ZMEL1 cells showed significantly higher dissemination beyond the yolk sac compared with proliferative cells (new Fig. EV5K). Strikingly, PYGi treatment of larvae injected with proliferative ZMEL1 cells was sufficient to increase their invasive capacity to levels comparable to those of untreated invasive cells (new Fig. 9H–I), demonstrating that acute pharmacological blockade of glycogen mobilization phenocopies the invasive state in vivo without additional genetic alterations.

Colorectal cancer progression model. The KM12C/KM12SM isogenic pair, in which the KM12SM subline was derived from spontaneous hepatic metastasis, independently recapitulates the inverse relationship between glycogen mobilization capacity and metastatic aggressiveness observed in melanoma (new Fig. EV5D–E), extending the in vivo evidence to a second tumor type.

Together, these three systems provide robust and complementary evidence that glycogen phosphorylase activity restrains tumor cell invasiveness and that its pharmacological inhibition promotes a pro-invasive phenotype across species and tumor types. Combined with our comprehensive clinical data spanning nevi, primary melanomas, and local and distant metastases, we believe the revised manuscript now provides the in vivo and clinical validation necessary to support our conclusions.

- The rationale provided for the selection of melanoma cell lines is not sufficient. Given the high degree of inter-line heterogeneity in genetic drivers (e.g., BRAF, NRAS, PTEN status), failure to stratify or account for these factors may have an impact on the described phenotypes. The authors need to clarify whether the observed effects are consistent across lines with different genetic backgrounds.

We thank the reviewer for raising this point and agree that genetic heterogeneity across cell lines should be accounted for. We have addressed this concern at multiple levels in the revised manuscript.

The primary cell line pair used for mechanistic analyses (IGR37 and IGR39) represents an isogenic model: both lines are derived from the same patient, with IGR37 originating from a metastasis and IGR39 from a primary tumor, and both carry the $BRAF^{V600E}$ mutation. This isogenic design minimizes the confounding influence of genetic background and allows the observed differences in glycogen metabolism and invasive behaviour to be attributed to phenotypic state rather than driver mutation status. This is now explicitly stated in the Results section (line 190-196).

To demonstrate that our findings are not restricted to a single genetic background, we extended our analysis to an in-house panel of 12 melanoma cell lines spanning the full spectrum of proliferative and invasive

phenotypes (new Fig. 2E), annotated for the most prevalent driver mutations in melanoma, including BRAF, NRAS, and PTEN alterations, collectively covering more than 80% of common melanoma genotypes. The correlation between MITF expression and glycogen metabolism enzyme levels was consistent across lines regardless of driver mutation status, indicating that the relationship between the MITF-defined phenotypic state and glycogen metabolism is independent of specific genetic backgrounds. This annotation has been added to Fig. 2E and described explicitly in the Methods section.

These findings were further extended to an independent panel of 53 well-characterized melanoma cell lines from the Cancer Cell Line Encyclopedia (CCLE), ranked by MITF expression (Fig. 2F). Results broadly recapitulated those of our in-house panel across a diverse range of genotypes, supporting the robustness and generalizability of the association between MITF status and glycogen metabolism enzyme expression.

As supplementary evidence, we now include glycogen content measurements in additional melanoma cell lines representing MITFHigh, $MITF^{Low}$, and intermediate phenotypes across different genetic backgrounds (new Fig. EV1B), further confirming that glycogen accumulation tracks with MITF-defined phenotypic state rather than with any specific driver mutation.

Taken together, these data demonstrate that the relationship between MITF-defined phenotypic state and glycogen metabolism is a robust, feature of melanoma cell biology.

- While glycogen metabolism is the main focus, many other metabolic pathways can influence cell behavior. The authors do not show whether these other factors were considered or ruled out. This weakens the argument that glycogen is the key driver of the observed effects. The authors might consider altering the title by replacing metabolic buffering with glycogen buffering.

We thank the reviewer for this thoughtful comment. We agree that other metabolic pathways can influence cell behaviour and that our manuscript does not systematically rule out their contribution. We have addressed this concern in two ways.

Other metabolic pathways. Our study does not claim that glycogen is the sole determinant of phenotypic switching, and we have revised the manuscript to make this explicit. We acknowledge that glycolysis, oxidative phosphorylation, lipid metabolism, and nucleotide biosynthesis can all influence, and be influenced by, glycogen status. Indeed, our own data illustrate this interconnectedness: $MITF^{High}$ cells display higher glycolytic flux as measured by ECAR, and glucose deprivation provokes metabolic adaptations that extend beyond glycogen mobilization alone. We have strengthened the Discussion to explicitly state that glycogen implements its buffering function within a wider metabolic network, that glycogen depletion provokes downstream adaptations that may contribute to phenotype switching, and that identifying complementary or alternative metabolic drivers represents an important direction for future work.

Critically, however, the causal role of glycogen in the observed phenotypic effects is supported by multiple independent and mechanistically distinct perturbations, genetic depletion of PGM1, pharmacological inhibition of glycogen phosphorylase, and gain-of-function PGM1 overexpression, all of which converge on the same phenotypic outcome regardless of genetic background or experimental context. This convergence argues strongly that glycogen metabolism is a key, though not necessarily exclusive, driver of the observed effects.

Regarding the title. We have carefully considered the reviewer's suggestion to replace "metabolic buffering" with "glycogen buffering." We opted to retain "metabolic buffering" because it more accurately reflects the conceptual advance of our work: glycogen is presented as a central, experimentally tractable example of a broader class of cellular energy reservoirs that modulate phenotype stability, and this framing intentionally positions our findings within the wider context of metabolic regulation of cancer cell plasticity. Nevertheless, we recognize that this terminology may imply a broader mechanistic claim than our data directly support. As a compromise, we have added clarifying language in the Abstract, Introduction, and Discussion to make explicit that "metabolic buffering" refers specifically to glycogen-mediated buffering in the context of this study, while acknowledging that other metabolic reservoirs may contribute to phenotype stabilization in different contexts.

- All figures should include sample sizes and mention the number of biological replicates that were used. This is in particular important for the immunoblots depicted throughout the manuscript.

We agree that all figures must report sample sizes and numbers of biological replicates, and we apologize for this omission in the original submission. Each western blot shown in the manuscript is representative of 3–5 independent biological replicates. We have systematically reviewed all figure panels and legends throughout the revised manuscript and updated them to explicitly state the exact n for each experiment, the number of biological replicates, and quantification where appropriate. These changes are reflected throughout the revised figures and their legends.

- The authors might want to consult the following publication: PMID: 9648905, Arch Pathol Lab Med. 1998 Apr;122(4):353-60.

We thank the reviewer for drawing our attention to this reference. We have obtained the paper (Nowak et al., 1998, Arch Pathol Lab Med) and confirm that it documents the frequency and patterns of PAS positivity and glycogen-rich histological variants in primary and metastatic melanomas, providing important historical precedent for glycogen accumulation in melanoma tissue. However, it does not examine the relationship between glycogen staining and invasive phenotypes or metastatic behaviour, nor does it provide mechanistic insights relevant to our study. We have cited this work as historical context in the revised Discussion and note that our study extends this descriptive precedent by demonstrating, for the first time, that glycogen content and mobilization capacity are functionally linked to phenotypic state and metastatic progression in melanoma.

Referee #3:

Ramirez-Sanchez and colleagues provide novel insights that in melanoma, the capacity to store and mobilize glycogen plays a critical role in maintaining the proliferative phenotype and preventing transition to invasion. The authors nicely demonstrate that high glycogen levels are characteristic of proliferative melanoma cells, whereas low glycogen levels are associated with an invasive phenotype. The experimental results show that a loss of glycogen storage/metabolism capacity leads to phenotypic instability and a switch to invasiveness. In addition, they demonstrate that low expression of glycogen phosphorylases and phosphoglucomutase 1 is associated with worse overall survival. Overall, this is a very well-written and presented manuscript describing an interesting mechanism of metabolic adaptations of cancer cells. The authors present their findings clearly and describe them accurately within the text using imaging approaches.

Below are comments that aim to further increase the clarity and significance of this exciting study:

1. Can the authors shift the metabolic profile of MITF(low) cells by enforcing glycogen accumulation? Is there a difference in proliferation between MITF(low) and MITF(high) melanoma cells? Do MITF(low) and MITF(high) melanoma cells take up the same level of glucose?

We thank the reviewer for these important questions and address each in turn.

Can the authors shift the metabolic profile of $MITF^{Low}$ cells by enforcing glycogen accumulation?

Yes. We directly addressed this by overexpressing PGM1 in WM793 cells, an invasive $MITF^{Low}$ line characterized by low endogenous PGM1 levels and negligible glycogen accumulation. PGM1 overexpression was sufficient to promote intracellular glycogen deposition, as confirmed by PAS staining, and was accompanied by a trend toward reduced extracellular glucose levels consistent with increased glucose routing toward glycogen synthesis (new Fig. 4G–H and EV4C–D). Strikingly, this metabolic shift was associated with a significant reduction in invasive capacity in Matrigel assays, phenocopying the behaviour of $MITF^{High}$ cells with high endogenous PGM1 expression. These data demonstrate that enforcing glycogen accumulation in $MITF^{Low}$ invasive cells is sufficient to shift both their metabolic state and phenotypic behaviour toward a less invasive program.

In addition, re-expression of MITF in vemurafenib-resistant A375 cells, which acquire a $MITF^{High}$ state during resistance, increased expression of glycogen metabolism enzymes PGM1 and PYGL, promoted glycogen accumulation, and produced a metabolic shift toward greater glucose utilization flexibility as assessed by Seahorse extracellular flux analysis (new Fig. 6I–M). Phenotypically, MITF re-expression reduced invasive capacity compared with parental cells (new Fig. 6L), further supporting the notion that driving a $MITF^{High}$/glycogen-mobilizing program is sufficient to shift both metabolism and phenotype.

Is there a difference in proliferation between MITFLow and MITFHigh melanoma cells?

Yes. $MITF^{Low}$ cells grow more slowly than $MITF^{High}$ cells, consistent with prior reports linking MITF status to proliferative capacity (e.g. Goodall et al., 2008, PMID: 18829533). To enable direct crossline comparisons, we have re-expressed all proliferation curves normalized to the IGR37 line in new Fig. EV3G, which reveals an even larger basal proliferation difference between $MITF^{High}$ and $MITF^{Low}$ lines than previously apparent.

Do MITFLow and MITFHigh melanoma cells take up the same level of glucose?

No. $MITF^{High}$ and $MITF^{Low}$ melanoma cells differ substantially in glucose uptake capacity and glucose transporter expression profiles. Analysis of CCLE RNA-seq data reveals variable GLUT family expression across lines, with SLC2A10 and SLC2A11 (also known as GLUT10 and GLUT11) predominating in $MITF^{High}$ cells (new Fig. EV1C). Functionally, basal glucose uptake measured with the fluorescent probe 2-NBDG showed that $MITF^{High}$ cells take up substantially more glucose than $MITF^{Low}$ cells, with $MITF^{Low}$ lines capturing approximately 60% less glucose (new Fig. EV1E–F). This difference is consistent with the higher glycolytic flux and glycogen synthesis rates observed in $MITF^{High}$ cells and further supports the notion that glucose availability and utilization are tightly coupled to phenotypic state in melanoma.

2. The authors should provide an overview of the signaling pathways that regulate glycogen synthesis in melanoma.

We thank the reviewer for this valuable suggestion. In response, we have included in the Discussion a concise summary of the major signalling pathways that regulate glycogen synthesis and mobilization in the context of melanoma phenotypic states (revised Discussion, lines 572-581), to maintain the flow of the Discussion, while providing a more detailed account here for completeness.

Briefly, growth factor signalling through RTKs activates PI3K–AKT, which inhibits GSK3 and thereby relieves inhibitory phosphorylation of GYS1, promoting glycogen accumulation and glucose uptake. mTORC1 integrates nutrient and growth signals to sustain anabolic fluxes that maintain a proliferative, glycogen-rich state. Conversely, energy stress activates AMPK and the integrated stress response, shifting cells toward catabolism and promoting glycogen mobilization. cAMP–PKA signalling activates phosphorylase kinase and PYG to trigger rapid glycogen breakdown in response to acute hormonal or stress cues. PP1 regulatory subunits stabilize glycogen stores by dephosphorylating and activating GYS1, thereby promoting glycogen synthesis, while simultaneously inhibiting PYG to suppress mobilization. Allosteric regulation provides minute-to-minute tuning independent of transcription: glucose-6-phosphate activates GYS1 to promote glycogen accumulation, while AMP enhances PYGB activity under energy stress to drive glycogen mobilization, and ATP/ADP suppress glycogenolysis. At the enzyme level, AMPK can induce PGM1 expression under glucose deprivation to sustain glycogen mobilization capacity, and PGM1 activity is further modulated by PAK1-mediated phosphorylation on threonine 466 (Kang et al., 2007). PYGB activity is enhanced by AMP, favouring mobilization, and suppressed by ATP, ADP, and glucose-6-phosphate, and may be further influenced by oncogenic PI3K/AKT and MAPK signalling, although direct mechanistic links remain under investigation.

Within this regulatory landscape, MITF adds a previously unrecognized transcriptional layer by directly controlling the expression of PGM1, PYGB, and PYGL, linking phenotype-defining circuitry to glycogen metabolic capacity. We believe this expanded account, together with the mechanistic model depicted in Fig. 8H, provides a sufficient overview of these regulatory relationships.

3. To better assess the metabolic differences between MITF(high) and MITF(low) melanoma cells, the authors should provide direct metabolic measurements of glycogen, G1P, G6P and other glycolytic metabolites.

We thank the reviewer for this suggestion. In response, we have incorporated direct metabolomic measurements that strengthen the mechanistic basis of our conclusions. Steady-state levels of glycogen-related intermediates (G1P, G6P, UDP-glucose) and lactate were quantified across $MITF^{High}$ and $MITF^{Low}$ melanoma lines by capillary electrophoresis mass spectrometry (CE-MS), and are presented in revised Fig. EV2A–D and new Fig. 6E–H.

IGR37 cells, which harbour the highest intracellular glycogen stores, displayed the lowest levels of G6P, G1P, and UDP-glucose, consistent with efficient channelling of glucose-6-phosphate into glycogen synthesis and consequent rapid consumption of upstream intermediates. In contrast, IGR39 cells, which rely predominantly on OXPHOS and do not accumulate glycogen, exhibited more balanced G6P/G1P ratios, reflecting reduced commitment of G6P to pyruvate production and greater availability for conversion to G1P and UDP-glucose despite the absence of net glycogen synthesis. Intracellular lactate levels were broadly similar across lines, suggesting a tight balance between lactate production and export that maintains intracellular pH and redox homeostasis, consistent with the higher extracellular acidification rates observed in $MITF^{High}$ cells by ECAR.

Accordingly, MITF depletion by siRNA produced metabolic changes in 501mel cells consistent with impaired glycogen utilization: elevated G1P, UDP-glucose, and intracellular lactate levels compared with control cells, while the G1P/G6P ratio increased, recapitulating the metabolic profile characteristic of $MITF^{Low}$ cells. These data indicate that MITF loss redirects glucose flux away from glycogen mobilization and toward glycolytic intermediary accumulation, supporting the model that MITF-dependent transcriptional control of glycogen metabolism enzymes shapes the metabolic state of melanoma cells.

Together, these metabolomic measurements provide direct quantitative evidence for the metabolic differences between MITF^{High} and MITF^{Low} cells and support the mechanistic framework proposed in our study.

4. Can the authors provide a mechanistic insight in how MITF(high) melanoma cells mobilize glycogen under glucose-limiting conditions compared to MITF(low) cells lacking this capability. What is regulating the expression of PGM1/PYGB, are these direct target genes of MITF?

We thank the reviewer for this important question. Our data provide mechanistic insight into how MITF^{High} melanoma cells retain the ability to mobilize glycogen under glucose limitation while MITF^{Low} cells lack this capability and clarify the extent to which PGM1 and PYGB are under direct MITF transcriptional control.

MITF as a direct transcriptional regulator of glycogen metabolism enzymes. Analysis of TCGA melanoma samples revealed strong correlations between MITF expression and that of GYS1 ($r = 0.250$; $p = 3.3 \times 10^{-8}$), PYGB ($r = 0.126$; $p = 0.006$) and PYGL ($r = 0.249$; $p = 3.85 \times 10^{-8}$) (new Fig. 6A). MITF ChIP-seq in 501mel cells identified binding peaks at the PGM1, PYGB and PYGL loci, consistent with direct transcriptional regulation (new Fig. 6B). Functional validation confirmed this regulatory link: MITF knockout in B16 cells reduced PYGL protein levels (new Fig. 6C), and MITF depletion by siRNA in 501mel cells significantly reduced intracellular glycogen content as evidenced by PAS staining (new Fig. 6D), indicating that MITF activity is required to maintain glycogen stores in MITF^{High} melanoma cells.

Metabolomic evidence for impaired glycogen flux upon MITF loss. MITF depletion in 501mel cells disrupted glycogen-related metabolite routing: the G1P/G6P ratio decreased, and UDP-glucose accumulated (new Fig. 6E–G), consistent with impaired conversion of glucose-6-phosphate into glycogen and accumulation of upstream precursors. Intracellular lactate was also elevated (new Fig. 6H), suggesting altered glycolytic flux or lactate handling upon MITF loss. These metabolomic changes recapitulate the profile characteristic of MITF^{Low} cells, demonstrating that MITF-dependent transcriptional control directly shapes glucose routing in melanoma cells.

Taken together, these data indicate that MITF regulates PGM1, PYGB, and PYGL, and that effective glycogen mobilization in MITF^{High} cells emerges from the integration of transcriptional, post-transcriptional, and allosteric regulatory inputs. These mechanistic insights are presented in new Fig. 6.

5. Do PGM1 knockdown melanoma cells show differences in the glycogen levels, basal/compensatory glycolysis, glycolytic capacity and reserve? Do PGM1 knockdown melanoma cells take up more glucose?

We thank the reviewer for these questions, which guided us to better characterize the metabolic consequences of PGM1 knockdown.

Glycogen levels. PGM1 knockdown in 501mel and IGR37 cells did not detectably alter glycogen content under basal glucose conditions, as assessed by PAS staining and quantification. However, under low-glucose conditions, PGM1-depleted cells failed to reduce their glycogen stores, in contrast to control cells which mobilized glycogen efficiently (new Fig. EV4A). This is consistent with the role of PGM1 in catalysing the conversion of G1P to G6P during glycogen breakdown, its loss prevents the utilization of stored glycogen as an alternative fuel source when extracellular glucose is limited.

Glycolytic parameters. Seahorse extracellular flux analysis revealed that under basal glucose conditions, PGM1 knockdown produced no consistent change in basal ECAR but showed a trend toward reduced glycolytic reserve that did not reach statistical significance. By contrast, under glucose-depleted conditions, PGM1 knockdown markedly impaired glycolytic capacity (50% reduction) and caused a large, statistically significant decline in glycolytic reserve (new Fig. EV3H–J). These effects closely mirrored those produced by pharmacological inhibition of glycogen phosphorylase, indicating that the most pronounced functional consequences of PGM1 loss are revealed under metabolic stress, consistent with a role for PGM1 in preserving glycolytic flexibility when extracellular glucose is limited.

Glucose uptake. Using a colorimetric glucose consumption assay, we found that both PYGi treatment and PGM1 knockdown reduced residual glucose in the culture medium compared with controls (new Fig. EV4B), indicating increased net glucose consumption when internal glycogen buffering is disabled. This

supports the interpretation that PGM1-depleted cells compensate for impaired glycogen mobilization by increasing their reliance on extracellular glucose, consistent with a compensatory metabolic response to the loss of internal energy reserves.

6. Does the loss of PGM1-mediated glycogen metabolism trigger a transcriptional reprogramming that leads to an invasive phenotype in melanoma cells?

We thank the reviewer for this important question. A full transcriptomic characterization of PGM1 knockdown cells was beyond the scope of the present study. Our functional data demonstrate that PGM1 loss impairs glycogen mobilization and increases invasive capacity under glucose-limiting conditions, consistent with a shift toward an invasive phenotype. Whether this reflects transcriptional reprogramming or is mediated through non-transcriptional mechanisms, including allosteric regulation, altered substrate flux, redox changes, or post-transcriptional modulation, remains an important open question that we identify as a key direction for future work.

7. The increase in invasiveness of the PGM1-depleted 501mel cells should be tested in mice.

We thank the reviewer for this important suggestion and fully agree that in vivo validation of the invasive phenotype is essential. We note that 501mel cells are non-tumorigenic in mice (Ohanna et al., 2011; PMID: 21646373) and are therefore not suitable for direct allograft or spontaneous metastasis assays. However, we have addressed the spirit of this comment through two complementary in vivo approaches that directly test whether impaired glycogen mobilization promotes invasiveness, presented in new Figure 9.

Murine allograft model. We implanted B16-F10 melanoma cells, which, like 501mel, display a proliferative $MITF^{High}$ phenotype and accumulate intracellular glycogen, subcutaneously into athymic nude mice and treated animals with the glycogen phosphorylase inhibitor CP-91441 (PYGi) to pharmacologically block glycogen mobilization. Vehicle-treated mice developed significantly larger primary tumors and overt metastases, whereas PYGi-treated animals showed reduced primary tumor growth and failed to develop metastases. This result can be attributed either to a cell-autonomous effect or to systemic blockade of glycogen phosphorylase activity, which may limit glycogen bioavailability in metastatic target organs such as the liver, rendering these tissues less permissive to tumor colonization or to proliferation of invading. Importantly, histological stratification of untreated primary tumors by metastatic outcome showed that tumors from mice that developed metastases exhibited significantly higher PAS staining and elevated PGM1 and PYGB expression compared with non-metastatic tumors, directly recapitulating the pattern observed in human primary melanoma and confirming that intratumoral glycogen metabolism correlates with metastatic capacity in vivo. Matched lung metastases showed markedly lower PAS staining and reduced enzyme expression compared with their corresponding primary tumors, mirroring the human clinical data (new Fig. 9 and EV5J).

Zebrafish larval transplantation model. To directly visualize early tumor cell invasion in vivo and circumvent the limitations of murine models or 501mel cells, zebrafish larval transplantation system was used. Pharmacological inhibition of glycogen phosphorylase with PYGi (CP 91441) in larvae injected with proliferative ZMEL cells, which accumulate glycogen and phenotypically resemble $MITF^{High}$/501mel cells, was sufficient to increase their invasive capacity to levels comparable to those of untreated invasive ZME1L cells (new Fig. 9H–I and new EV5K). This demonstrates that acute blockade of glycogen mobilization phenocopies the invasive state in vivo at single-cell resolution, providing direct evidence that impaired glycogen mobilization promotes invasiveness independently of genetic manipulation.

Together, these two independent in vivo models provide robust evidence that pharmacological impairment of glycogen mobilization, mimicking the metabolic consequence of PGM1 loss, promotes invasive behaviour across experimental systems.

8. In Figure 1G/H/I the data for the melanoma cell line WM793 is missing.

We thank the reviewer for noting this omission. WM793 data have been added to new Fig. 1G–H–I. As expected, given its $MITF^{Low}$ invasive phenotype, WM793 results mirror those of IGR39, showing lower glycolytic flux and reduced dependence on glucose metabolism compared with $MITF^{High}$ cell lines. The

figure panels and legends have been updated accordingly, statistical comparisons have been revised, and the results are reflected in the main text (lines 136-147).

1st Revision - Editorial Decision 29th Apr 2026

Dear Custodia,

We have now received re-review reports from two referees, which I have included below. As you will see, you have addressed their concerns satisfactorily. Before I can finally accept the manuscript, there are some remaining editorial points which need to be addressed. In this regard would you please:

- acknowledge the following funding in our online submission system: Ludwig Institute for Cancer Research; World Premier International Research Center Initiative (WPI), Human Biology-Microbiome-Quantum Research Center (Bio2Q);
include a "Disclosure and competing interests statement"): employment in a biotech company should be stated here,
- remove the AC/CrediT section from the text,
- ensure that all figure callouts in the manuscript text are sequential,
- remove the 'Reagents and Tools' table from the main manuscript file,
- provide specific specific URLs for GSE137522, GSE137392 datasets in the data availability statement,
- define p values annotated as ****/***/**/*,
- provide exact p values in the legends of figures 1E, F, H, I; 2B, H, K; 3B, C, D, F, G, H, J, K, L; 4C, F, H; 5A-D; 6E, G, H, J, L, M; 9C, I; EV1 D, F; EV2 A-D; EV3 B, F, G, J, K; EV4 B, D, E, F,
- state the statistical test used for data analysis in the legend of figure EV3 A,
- define box plots in terms of minima, maxima, centre, bounds of box and whiskers, and percentile in the legends of figures 7A-H; 8A, C, D, E; EV5 C,
- define n in the legends of figures 7E-H; EV3 A, I,
- define error bars in the legends of figures 1B, G; 3A, E, I; 4, F, H; 5A, 6M, 9E, F, I; EV3 A, I
- define the the measure of centre for error bars in the legends of figures EV2 A-D
- remove the "Highlights" section from thre manuscript, and
- include the "Ethics Statements" in the "Methods" section,

We include a synopsis of the paper (see http://emboj.embopress.org/). Please provide me with a general summary image, a two sentence statement and 3-5 bullet points that capture the key findings of the paper.

I am looking forward to receiving your revised manuscript.

EMBO Press is an editorially independent publishing platform for the development of EMBO scientific publications.

Best wishes,

William

William Teale, PhD
Editor
The EMBO Journal
w.teale@embojournal.org

Instructions for preparing your revised manuscript:

Read our guidance for manuscript revisions and related editorial policies: https://link.springer.com/journal/44318/submission-guidelines#cms-Revised-submissions

Please also check that the title and abstract of the manuscript are brief, yet explicit, even to non-specialists.

When assembling figures, please refer to our figure preparation guideline in order to ensure proper formatting and readability in print as well as on screen:
https://media.springernature.com/original/springer-cms/rest/v1/content/27825798/data/v1

IMPORTANT: When you send the revision we will require
- a point-by-point response to the referees' comments, with a detailed description of the changes made (as a word file).
- a word file of the manuscript text.
- individual production quality figure files (one file per figure)
- a complete author checklist
- Expanded View files (replacing Supplementary Information)
- a Reagents and Tools Table as part of the Methods section

Please remember: Digital image enhancement is acceptable practice, as long as it accurately represents the original data and conforms to community standards. If a figure has been subjected to significant electronic manipulation, this must be noted in the figure legend or in the 'Methods' section. The editors reserve the right to request original versions of figures and the original images that were used to assemble the figure.

We realize that it is difficult to revise to a specific deadline. In the interest of protecting the conceptual advance provided by the work, we recommend a revision within 3 months (28th Jul 2026). Please discuss the revision progress ahead of this time with the editor if you require more time to complete the revisions. Use the link below to submit your revision:

https://emboj.msubmit.net/cgi-bin/main.plex

Referee #2:

The authors provide a substantially revised manuscript containing a considerable amount of new data. They have also addressed most of my comments and suggestions. Overall, the revised version is significantly improved.
A minor concern regarding novelty remains. In addition, the authors may want to consider adjusting the title to use controls rather than suppresses and melanoma instead of cancer.

Referee #3:

This is a revised version of the previously submitted manuscript from Ramirez-Sanchez and colleagues. The authors have performed a thorough revision of the manuscript and included extended series of novel observations and data.

The authors have fully addressed all of my comments, and therefore I recommend this work to be accepted for publication.

All editorial and formatting issues were resolved by the authors.

2nd Revision - Editorial Decision 26th Jun 2026

Dear Dr. Chocarro-Calvo,

I am pleased to inform you that your manuscript has been accepted for publication in the EMBO Journal.

Congratulations to you and all involved!

Your manuscript will be processed for publication by EMBO Press. It will be copy edited and you will receive page proofs prior to publication. Please note that you will be contacted by Springer Nature Author Services to complete licensing and payment information.

You may qualify for financial assistance for your publication charges - either via a Springer Nature fully open access agreement or an EMBO initiative. Check your eligibility: https://link.springer.com/journal/44318/how-to-publish-with-us

Should you be planning a Press Release on your article, please notify the production team via this form in order to coordinate publication and release dates: https://forms.cloud.microsoft/e/eH4A8JVP6g

If you have any questions, please do not hesitate to contact the Editorial Office. Thank you for your contribution to The EMBO Journal.

Yours sincerely,

William Teale

William Teale, PhD
Editor
The EMBO Journal
w.teale@embojournal.org

*****
Please note that it is The EMBO Journal policy for the transcript of the editorial process (containing referee reports and your response letters) to be published as an online supplement to each paper. If you should prefer removal of any referee-only figures included in the point-by-point response(s), e.g. because they may still be used for future publication or because they have been reproduced from published work by others, please do let us know immediately via response email.
More information is available here: https://link.springer.com/partners/embo-press/editorial-policies#Peer%20review

** Click here to be directed to your login page: https://emboj.msubmit.net

EMBO Press Author Checklist

Corresponding Author Name: Ana Chocarro Calvo / Custodia García Jiménez
Journal Submitted to: The EMBO Journal
Manuscript Number: EMBOJ-2025-121479R

USEFUL LINKS FOR COMPLETING THIS FORM
The EMBO Journal - Author Guidelines
EMBO Reports - Author Guidelines
Molecular Systems Biology - Author Guidelines
EMBO Molecular Medicine - Author Guidelines

Reporting Checklist for Life Science Articles (updated January
This checklist is adapted from Materials Design Analysis Reporting (MDAR) Checklist for Authors. MDAR establishes a minimum set of requirements in transparent reporting in the life sciences (see Statement of Task: 10.31222/osf.io/9sm4x). Please follow the journal's guidelines in preparing your
Please note that a copy of this checklist will be published alongside your article.

Abridged guidelines for figures
1. Data
The data shown in figures should satisfy the following conditions:
- the data were obtained and processed according to the field's best practice and are presented to reflect the results of the experiments in an accurate and unbiased manner.
- ideally, figure panels should include only measurements that are directly comparable to each other and obtained with the same assay.
- plots include clearly labeled error bars for independent experiments and sample sizes. Unless justified, error bars should not be shown for technical
- if n<5, the individual data points from each experiment should be plotted. Any statistical test employed should be justified.
- Source Data should be included to report the data underlying figures according to the guidelines set out in the authorship guidelines on Data

2. Captions
Each figure caption should contain the following information, for each panel where they are relevant:
- a specification of the experimental system investigated (eg cell line, species name).
- the assay(s) and method(s) used to carry out the reported observations and measurements.
- an explicit mention of the biological and chemical entity(ies) that are being measured.
- an explicit mention of the biological and chemical entity(ies) that are altered/varied/perturbed in a controlled manner.
- the exact sample size (n) for each experimental group/condition, given as a number, not a range;
- a description of the sample collection allowing the reader to understand whether the samples represent technical or biological replicates (including how many animals, litters, cultures, etc.).
- a statement of how many times the experiment shown was independently replicated in the laboratory.
- definitions of statistical methods and measures:
  - common tests, such as t-test (please specify whether paired vs. unpaired), simple χ2 tests, Wilcoxon and Mann-Whitney tests, can be unambiguously identified by name only, but more complex techniques should be described in the methods section;
  - are tests one-sided or two-sided?
  - are there adjustments for multiple comparisons?
  - exact statistical test results, e.g., P values = x but not P values < x;
  - definition of 'center values' as median or average;
  - definition of error bars as s.d. or s.e.m.

Please complete ALL of the questions below.
Select "Not Applicable" only when the requested information is not relevant for your study.

Materials

Newly Created Materials	Information included in the manuscript?	In which section is the information available? (Reagents and Tools Table, Materials and Methods, Figures, Data Availability Section)
New materials and reagents need to be available; do any restrictions apply?	Not Applicable	

Antibodies	Information included in the manuscript?	In which section is the information available? (Reagents and Tools Table, Materials and Methods, Figures, Data Availability Section)
For **antibodies** provide the following information:<br>- Commercial antibodies: RRID (if possible) or supplier name, catalogue number and or/clone number<br>- Non-commercial: RRID or citation	Yes	Reagents and Tools Table

DNA and RNA sequences	Information included in the manuscript?	In which section is the information available? (Reagents and Tools Table, Materials and Methods, Figures, Data Availability Section)
**Short novel DNA or RNA including primers, probes**: provide the sequences.	Yes	Reagents and Tools Table

Cell materials	Information included in the manuscript?	In which section is the information available? (Reagents and Tools Table, Materials and Methods, Figures, Data Availability Section)
**Cell lines:** Provide species information, strain. Provide accession number in repository **OR** supplier name, catalog number, clone number, and/**OR** RRID.	Yes	Reagents and Tools
**Primary cultures:** Provide species, strain, sex of origin, genetic modification status.	Not Applicable	
Report if the cell lines were recently **authenticated** (e.g., by STR profiling) and tested for mycoplasma contamination.	Yes	Material and methods

Experimental animals	Information included in the manuscript?	In which section is the information available? (Reagents and Tools Table, Materials and Methods, Figures, Data Availability Section)
**Laboratory animals or Model organisms:** Provide species, strain, sex, age, genetic modification status. Provide accession number in repository **OR** supplier name, catalog number, clone number, **OR** RRID.	Yes	Reagents and Tools Table, Material and methods
**Animal observed in or captured from the field:** Provide species, sex, and age where possible.	Not Applicable	
Please detail **housing and husbandry conditions**.	Yes	Material and methods

Plants and microbes	Information included in the manuscript?	In which section is the information available? (Reagents and Tools Table, Materials and Methods, Figures, Data Availability Section)
**Plants:** provide species and strain, ecotype and cultivar where relevant, unique accession number if available, and source (including location for collected wild specimens).	Not Applicable	
**Microbes:** provide species and strain, unique accession number if available, and source.	Not Applicable	

Human research participants	Information included in the manuscript?	In which section is the information available? (Reagents and Tools Table, Materials and Methods, Figures, Data Availability Section)
If collected and within the bounds of privacy constraints report on age, sex and gender or ethnicity for all study participants.	Yes	Table I

Core facilities	Information included in the manuscript?	In which section is the information available? (Reagents and Tools Table, Materials and Methods, Figures, Data Availability Section)
If your work benefited from core facilities, was their service mentioned in the acknowledgments section?	Not Applicable	

Design

Study protocol	Information included in the manuscript?	In which section is the information available? (Reagents and Tools Table, Materials and Methods, Figures, Data Availability Section)
If study protocol has been **pre-registered, provide DOI in the manuscript**. For clinical trials, provide the trial registration number **OR** cite DOI.	Not Applicable	
Report the **clinical trial registration number** (at ClinicalTrials.gov or equivalent), where applicable.	Not Applicable	

Laboratory protocol	Information included in the manuscript?	In which section is the information available? (Reagents and Tools Table, Materials and Methods, Figures, Data Availability Section)
Provide DOI OR other citation details if **external detailed step-by-step protocols** are available.	Not Applicable	

Experimental study design and statistics	Information included in the manuscript?	In which section is the information available? (Reagents and Tools Table, Materials and Methods, Figures, Data Availability Section)
Include a statement about **sample size** estimate even if no statistical methods were used.	Yes	Materials and Methods
Were any steps taken to minimize the effects of subjective bias when allocating animals/samples to treatment (e.g. **randomization procedure**)? If yes, have they been described?	Not Applicable	
Include a statement about **blinding** even if no blinding was done.	Not Applicable	
Describe **inclusion/exclusion criteria** if samples or animals were excluded from the analysis. Were the criteria pre-established? If sample or data points were omitted from analysis, report if this was due to attrition or intentional exclusion and provide justification.	Not Applicable	
For every figure, are **statistical tests** justified as appropriate? Do the data meet the assumptions of the tests (e.g., normal distribution)? Describe any methods used to assess it. Is there an estimate of variation within each group of data? Is the variance similar between the groups that are being statistically compared?	Yes	Material and Methods, Figure legends

Sample definition and in-laboratory replication	Information included in the manuscript?	In which section is the information available? (Reagents and Tools Table, Materials and Methods, Figures, Data Availability Section)
In the figure legends: state number of times the experiment was **replicated** in laboratory.	Yes	Figure legends
In the figure legends: define whether data describe **technical or biological replicates**.	Yes	Figure legends

Ethics

Ethics	Information included in the manuscript?	In which section is the information available? (Reagents and Tools Table, Materials and Methods, Figures, Data Availability Section)
Studies involving **human participants**: State details of **authority granting ethics approval** (IRB or equivalent committee(s), provide reference number for approval.	Yes	Materials and Methods
Studies involving **human participants**: Include a statement confirming that **informed consent** was obtained from all subjects and that the experiments conformed to the principles set out in the WMA Declaration of Helsinki and the Department of Health and Human Services Belmont Report.	Yes	Materials and Methods
Studies involving **human participants:** For publication of **patient photos**, include a statement confirming that consent to publish was obtained.	Not Applicable	
Studies involving experimental **animals**: State details of **authority granting ethics approval** (IRB or equivalent committee(s), provide reference number for approval. Include a statement of compliance with ethical regulations.	Yes	Materials and Methods
Studies involving **specimen and field samples:** State if relevant **permits** obtained, provide details of authority approving study; if none were required, explain why.	Not Applicable	

Dual Use Research of Concern (DURC)	Information included in the manuscript?	In which section is the information available? (Reagents and Tools Table, Materials and Methods, Figures, Data Availability Section)
Could your study fall under dual use research restrictions? Please check biosecurity documents and list of **select agents and toxins** (CDC): https://www.selectagents.gov/sat/list.htm	Not Applicable	
If you used a select agent, is the security level of the lab appropriate and reported in the manuscript?	Not Applicable	
If a study is subject to dual use research of concern regulations, is the name of the **authority granting approval and reference number** for the regulatory approval provided in the manuscript?	Not Applicable	

Reporting

The MDAR framework recommends adoption of discipline-specific guidelines, established and endorsed through community initiatives. Journals have their own policy about requiring specific guidelines and recommendations to complement MDAR.

Adherence to community standards	Information included in the manuscript?	In which section is the information available? (Reagents and Tools Table, Materials and Methods, Figures, Data Availability Section)
State if relevant guidelines or checklists (e.g., **ICMJE, MIBBI, ARRIVE, PRISMA**) have been followed or provided.	Yes	Materials and Methods
For **tumor marker prognostic studies,** we recommend that you follow the **REMARK** reporting guidelines (see link list at top right). See author guidelines, under 'Reporting Guidelines'. Please confirm you have followed these guidelines.	Not Applicable	
For **phase II and III randomized controlled trials**, please refer to the **CONSORT** flow diagram (see link list at top right) and submit the CONSORT checklist (see link list at top right) with your submission. See author guidelines, under 'Reporting Guidelines'. Please confirm you have submitted this list.	Not Applicable	

Data Availability

Data availability	Information included in the manuscript?	In which section is the information available? (Reagents and Tools Table, Materials and Methods, Figures, Data Availability Section)
Have **primary datasets** been deposited according to the journal's guidelines (see 'Data Deposition' section) and the respective accession numbers provided in the Data Availability Section?	Yes	Data availability section
Were **human clinical and genomic datasets** deposited in a public access-controlled repository in accordance to ethical obligations to the patients and to the applicable consent agreement?	Not Applicable	
Are **computational models** that are central and integral to a study available without restrictions in a machine-readable form? Were the relevant accession numbers or links provided?	Not Applicable	
If publicly available data were reused, provide the respective **data citations in the reference list**.	Yes	Data Availability section
